# Mapping the diffusion pattern of ^1^O_2_ along DNA duplex by guanine photooxidation with an appended biphenyl photosensitizer

**DOI:** 10.1038/s41598-023-27526-2

**Published:** 2023-01-23

**Authors:** Takashi Kanamori, Shota Kaneko, Koji Hamamoto, Hideya Yuasa

**Affiliations:** School of Life Science and Technology, Tokyo Institute of Technology, J2-10 4259, Nagatsuta, Midoriku, Yokohama, 226-8501 Japan

**Keywords:** Biochemistry, Chemical biology, Chemistry

## Abstract

To realize nucleic acid-targeting photodynamic therapy, a photosensitizer should be attached at the optimal position on a complementary oligonucleotide, where a guanine photooxidation is maximized. Here we show the photooxidation of 22 DNA duplexes with varied lengths between a ^1^O_2_-generating biphenyl photosensitizer attached at a midchain thymine in a strand and the single guanine reactant in the other strand. The best photooxidation efficiencies are achieved at 9, 10, and 21 base intervals, which coincides with the pitch of 10.5 base pairs per turn in a DNA duplex. The low efficiencies for near and far guanines are due to quenching of the biphenyl by guanine and dilution of ^1^O_2_ by diffusion, respectively. The ^1^O_2_-diffusion mapping along DNA duplex provides clues to the development of efficient and selective photosensitizer agents for nucleic acid-targeting photodynamic therapy, as well as an experimental demonstration of diffusion of a particle along cylindrical surface in molecular level.

## Introduction

There is increasing interest in nucleic acid oxidation because it can cause cancer, aging and many diseases^1–5^. More recent studies have shown that DNA oxidation can trigger epigenetic-like gene regulations^6–8^. Furthermore, photosensitized oxidation of DNA or RNA has been suggested to be one of cytotoxic mechanisms in photodynamic therapy (PDT)^9,10^. Accordingly, understanding of nucleic acid oxidation is becoming more and more important to decipher diseases and develop pharmaceuticals.

In the photooxidation of nucleic acids, guanine (G) is the most reactive part, oxidized by one-electron oxidation (type I)^11–13^ with reactive oxygen species (ROSs) including hydroxyl radical (HO^•^), carbonate radical anion (CO_3_^•-^), and hydrogen peroxide (H_2_O_2_) or by singlet oxygen (^1^O_2_) oxidation (type II)^14,15^, since G has the lowest oxidation potential in a nucleic acid molecule^16,17^.

In type I oxidation of DNA, charge migration through the array of π-stacked bases enables the oxidation of 5’-GG-3’ at a distance of about 20 nm far from the initial one-electron oxidation point^18–21^ and even 1.2-μm charge migration has been recorded for a DNA-mediated charge transport between repair proteins^22^. In addition, H_2_O_2_ produced in type I oxidation is stable enough to diffuse to neighboring cells, in which it can be converted into more cytotoxic HO^•^ or CO_3_^•-^ by metal ions.^3,4,23^ Therefore, type I oxidation of nucleic acid has a concern of indiscriminateness in terms of both base and target cell selectivities, when one aims for PDT.

On the other hand, ^1^O_2_ produced in type II oxidation has a relatively short lifetime^24,25^ in cells (*τ*_*1/2*_ ≈ 3 μs) and thus is mostly trapped in the cell in which it originated^23^. As opposed to type I oxidation, however, there was little information regarding how far and well ^1^O_2_ can reach and react with G along a DNA duplex after departing from a point it is released near the strand. We can only assume that the type II oxidation of G by ^1^O_2_ is much slower than that by type I oxidation^26^ and thus a ^1^O_2_-producing photosensitizer should be located as close to G as possible. In this regard, the conjugates of oligonucleotides (ONs) with photosensitizers have been synthesized and photooxidation of their duplexes or triplexes with DNA examined^27–33^. But in all cases, a relatively large photosensitizer, such as chlorin^29^, was linked to the 3' or 5' end of ON via a relatively long linker, which would let the photosensitizers sway in the bulk water far from the target base, resulting in poor resolution of selectivity in the G photooxidation both intra- and intermolecularly. Another problem is that many photosensitizers claimed as being type II have more or less type-I characteristics^34,35^, making the selectivity data less informative about ^1^O_2_ travelling along DNA. Therefore, neither precise nor detailed information about appropriate distance between G and a photosensitizer in ^1^O_2_ oxidation and ^1^O_2_ trajectory thereof have been obtained.

The authors have developed a type II biphenyl (BP) sensitizer that exclusively produces ^1^O_2_ (*Φ*_Δ_: 0.88) without generating reactive radicals and is much smaller in size than the previous photosensitizers^36,37^. If BP is linked at an appropriate position in ON with a short linker, the small BP molecule would be accommodated in or near the major groove in its duplex with DNA, restricting the departing point of ^1^O_2_ in a relatively small area and thus increasing resolution of G-oxidation selectivity. In this study, we explored the correlation between G-photooxidation efficiency and the G-to-BP distance in the duplexes of the BP-ON conjugates with DNAs. This study would provide the information about the traveling behavior of ^1^O_2_ along DNA duplexes in more detail than ever, thereby offering clues to overcome the low reactivity of type-II photooxidation of ON in a DNA- and/or RNA-targeting PDT by attaching BP at the most appropriate position of ON, where the target G would be most efficiently and selectively oxidized. In addition, the study could be a unique experimental demonstration of the diffusion of a particle along a cylindrical surface in molecular level, which is a challenging subject in diffusion science^38^.

## Results

**Figure 1:**
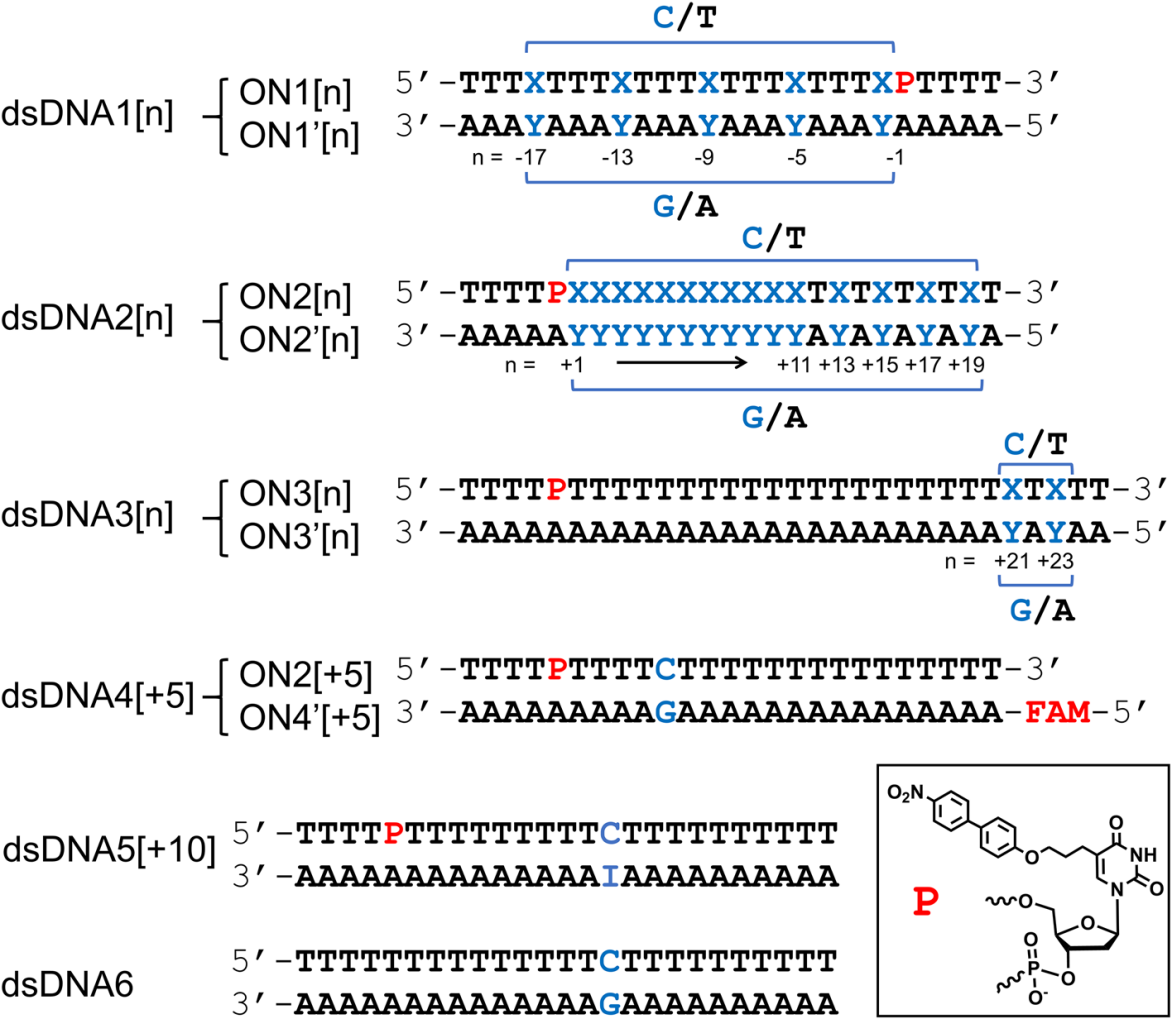
The double strand DNAs (dsDNA1[n], dsDNA2[n], dsDNA3[n], dsDNA4[+ 5]) composed of the synthesized oligonucleosides (ONs) incorporating nitrobipnenyl (BP) photosensitizer at the mononucleotide P (ON1[n], ON2[n], ON3[n]) and the corresponding complementary ONs (ON1’[n], ON2’[n], ON3’[n], ON4’[+ 5]), and the ONs (dsDNA5[+ 10], dsDNA6) synthesized for the intermolecular photooxidation study. X and Y denote C and G, respectively, at the n position and T and A at the other positions. FAM in ON4’[+ 5] is fluorescein amidite incorporated to enable fluorescence visualization in gel electrophoresis. ON1’[− 13], ON1’[− 9], ON1’[− 5], and ON1’[− 1] are identical with ON2’[+ 3], ON2’[+ 7], ON2’[+ 11], and ON2’[+ 15], respectively.

In designing BP-ON conjugates, we decided to attach the BP part at the α-methyl of thymine (T) through a freely rotatable ethylene spacer as depicted as P in Fig. 1, because it is synthetically accessible and the attached BP would be stuck out from the bottom of major groove without perturbing the nearby base-pair formation. The synthesis of P is described in Supplementary (Fig. S1).

P was incorporated in ONs (ON1[n], ON2[n], ON3[n]) so that the extent of separation between P and G covers two pitches of 10.5 base pairs per turn in both axial directions of B-DNA helices (Fig. 1)^39,40^. We defined n as the number of base intervals from P to cytidine (C) in 3’ directions of ON1, ON2, and ON3. Thus ON1[n]s with n = − 1, − 5, − 9, − 13, and − 17, ON2[n]s with n =  + 1 to + 11, + 13, + 15, + 17, and + 19, and ON3[n]s with + 21 and + 23 were synthesized (see Supplementary for details). The synthesized ONs were purified by HPLC and subjected to duplex formation with purchased ONs (ON1’[n], ON2’[n], ON3’[n], ON4’[+ 5]) to give the double strand DNAs (dsDNA1[n], dsDNA2[n], dsDNA3[n], dsDNA4[+ 5]). dsDNA5[+ 10] and dsDNA6 were prepared to study the intermolecular cross-talk photooxidation, in which inosine (I) was incorporated instead of G in dsDNA5[+ 10] to avoid intramolecular photooxidation.

The ^1^O_2_-producing quantum yield (*Φ*_Δ_) of BP-T mono-nucleoside by photoirradiation at 346 nm was determined to be 0.29 in DMF (Supplementary, Fig. S43). The reduced ^1^O_2_-producing efficiency compared with that of 4-methoxy-4’-nitrobiphenyl (*Φ*_Δ_: 0.88)^36^ is probably due to non-radiative quenching by collision with the N–H bond or photoelectron transfer quenching by the N atoms in T. Even so, the ^1^O_2_-producing efficiency was sufficient for the current study.

**Figure 2:**
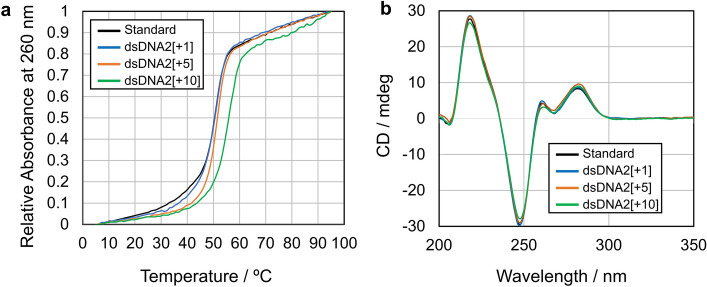
(**a**) Melting curves of dsDNA2[+ 1], dsDNA2[+ 5], and dsDNA2[+ 10] as compared with a standard dsDNA2[+ 1] without the BP photosensitizer. (**b**) CD spectra of dsDNA2[+ 1], dsDNA2[+ 5], and dsDNA2[+ 10] as compared with a standard dsDNA2[+ 1] without the BP photosensitizer.

*T*_*m*_ measurements were performed to verify that the addition of BP had no adverse effects on duplex formation. As a result, the melting values (51–55 °C) were the same as or slightly higher than that of the corresponding natural dsDNA (51 °C) for all the tested dsDNA1[n]s and dsDNA2[n]s (Table 1, Fig. 2a, Fig. S44). We can thus conclude that incorporation of BP in these ONs gives no stress on dsDNA formation thanks to the small photosensitizer and the freely rotatable ethylene linker.

**Table 1:** *T*_m_ values (°C) and the relative amounts of G and ^O^G (%) after photooxidation in H_2_O and D_2_O of dsDNAs.

n^a^	*T*_m_^b^	G in H_2_O	G in D_2_O	^O^G in H_2_O
− 17	55.6	88 ± 7	–	7 ± 2
− 13	54.8	58 ± 3	–	31 ± 4
− 9	54.3	38 ± 3	–	50 ± 3
− 5	55.0	78 ± 10	–	0
− 1	55.5	96 ± 1	–	0
+ 1	51.6	97 ± 3	–	0
+ 2	52.0	97 ± 1	–	0
+ 3	52.5	99 ± 4	–	0
+ 4	51.7	96 ± 1	–	0
+ 5	51.2	95 ± 3	98	0
+ 6	52.7	85 ± 10	–	0
+ 7	53.1	81 ± 3	–	18 ± 8
+ 8	52.2	43 ± 8	–	42 ± 11
+ 9	54.1	39 ± 8	–	34 ± 18
+ 10	52.5	36 ± 4	60	39 ± 15
+ 11	54.5	50 ± 1	–	38 ± 2
+ 13	53.5	49 ± 8	–	29 ± 12
+ 15	53.6	56 ± 7	70	18 ± 9
+ 17	54.5	63 ± 7	–	13 ± 2
+ 19	55.1	85 ± 4	89	0
+ 21	56.5	56 ± 5	–	18 ± 7
+ 23	57.0	66 ± 5	86	0

^a^dsDNA1[n], dsDNA2[n], and dsDNA3[n] were used respectively for n = − 17 to − 1, + 1 to + 19, and + 21 to + 23.

^b^Standard *T*_m_ value for the analog of dsDNA2[+ 1] without P was 51.2 ºC.

To asses if the dsDNA conformation is kept identical in these modified DNAs, circular dichroism (CD) spectra were measured for dsDNA2[+ 1], dsDNA2[+ 5], and dsDNA2[+ 10] as compared with a standard dsDSA2[+ 1] without the BP photosensitizer. The result again ensured the harmlessness of BP incorporation toward the strand structure of poly(dA):poly(dT) as shown in Fig. 2b ^41^.

We carried out the photooxidation of dsDNAs (2 μM) by shining LED light at 365 nm. Salt concentration (100 mM NaCl), pH (7.2 by 10 mM sodium phosphate) and temperature (25 °C) were kept constant during the reaction. The photooxidation products were digested to the mononucleosides with nucleases and they were quantified by HPLC (see Supplementary for details).

**Figure 3:**
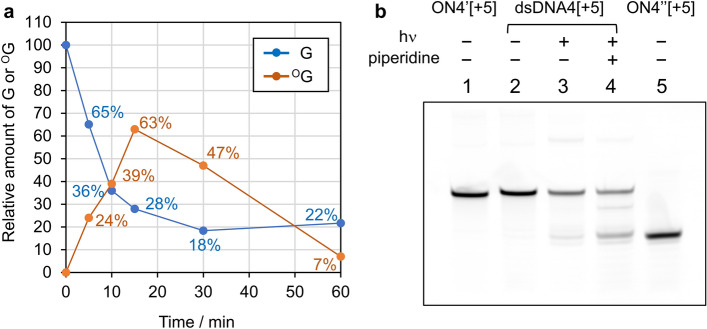
(**a**) Time course of the photooxidation of dsDNA2[+ 10]. (**b**) Gel electrophoresis analysis of the photooxidation products of dsDNA4[+ 5].

As shown in the time course of the photooxidation of dsDNA2[+ 10] (Fig. 3a), 8-oxodeoxyguanosine (^O^G) was mainly produced (39%) in the first 10 min, while 64% of G was consumed, and the degradation of ^O^G followed after 10 min probably because of overoxidation (see Supplementary for details).

In the previous studies on ^1^O_2_ oxidation and nuclease degradation of DNA, ^O^G has been mainly produced and otherwise only 2,2-diamino-4-[(2-deoxy-β-D-ribofuranosyl)amino]-5-(2*H*)-oxazolone (dOz) and *N*-(2-deoxy-β-D-ribofuranosyl)spiroiminodihydantoin (dSp) were obtained as the overoxidation products of ^O^G^15^. Although there might be up to 25% of byproducts at 10 min in our study, our assay system tracking absorption at 260 or 300 nm was incapable of detecting these products. Nevertheless, we were able to estimate the relative ^1^O_2_ concentration near G in dsDNAs by tracing G consumption as demonstrated below.

Since the initial consumption curve of G was linear as shown in Fig. 3a, we can apply the following pseudo-first-order kinetics of the consumption of G for the initial 10-min reaction with the premise that the concentration of ^1^O_2_, [^1^O_2_], at the oxidation site is almost constant during the reaction:

1 $$v = \, - {\text{d}}\left[ {\text{G}} \right]/{\text{d}}t = k_{rG} \left[ {\text{G}} \right] \, = k_{{r{\text{OG}}}} \left[ {^{{1}} {\text{O}}_{{2}} } \right]\left[ {\text{G}} \right]$$

where *v* is the rate of G consumption, *k*_*rG*_ is the pseudo-first-order rate constant, and *k*_*rOG*_ is the second-order rate constant. We can estimate *v* at *t* = 0 s from the concentration decrement of G at 10 min (600 s), Δ[G]_600_ = [G]_600_ – [G]_0_:

2 $$v \approx \, - \Delta \left[ {\text{G}} \right]_{{{6}00}} /{6}00 \, = k_{rOG} \left[ {^{{1}} {\text{O}}_{{2}} } \right]\left[ {\text{G}} \right]_{0}$$

where [G]_0_ is the initial concentration of G with a constant value of 2 μM throughout these experiments. When the concentrations of ^1^O_2_ near G_1_ and G_2_ are denoted as [^1^O_2_]_1_ and [^1^O_2_]_2_, respectively, the concentration ratio of ^1^O_2_ can be deduced as follows from the equation ii:

3 $$\left[ {^{{1}} {\text{O}}_{{2}} } \right]_{{1}} /\left[ {^{{1}} {\text{O}}_{{2}} } \right]_{{2}} \approx \Delta \left[ {{\text{G}}_{{1}} } \right]_{{{6}00}} /\Delta \left[ {{\text{G}}_{{2}} } \right]_{{{6}00}}$$

The equation iii means that we can estimate the relative concentration of ^1^O_2_ near G of interest by quantifying the G consumption within 10-min photoirradiation.

We next checked the site selectivity of the photooxidation without cleaving non-oxidized sites by treating the photooxidation (1-h irradiation at 365 nm) products of dsDNA4[+ 5] with hot piperidine (1 M at 90 °C for 40 min) to cleave only the oxidized sites and analyzing the resultant products by gel electrophoresis (Fig. 3b, Fig. S51). By using fluorescein amidite (FAM)-labeled ON (ON4’[+ 5]) and the truncated standard, 3’-A_15_-FAM-5’ (ON4’’[+ 5]), we were able to demonstrate that the photooxidation of dsDNA4[+ 5] was oxidized only at the target G sites (lane 4), whereas a large amount of non-cleaved ON4’[+ 5] was left intact. This intactness might be due to the low photooxidation reactivity of dsDNA4[+ 5] and also the inertness of some oxidation products toward hot piperidine treatment as reported in some studies^42,43^. It should be also noted that small amount of cleaved product was obtained without a hot piperidine treatment (lane 3). This might be due to the overoxidation products, which were prone to hydrolyze at *N*-ribofuranoside bonds leading to β-elimination of the phosphate groups without hot piperidine treatment.

**Figure 4:**
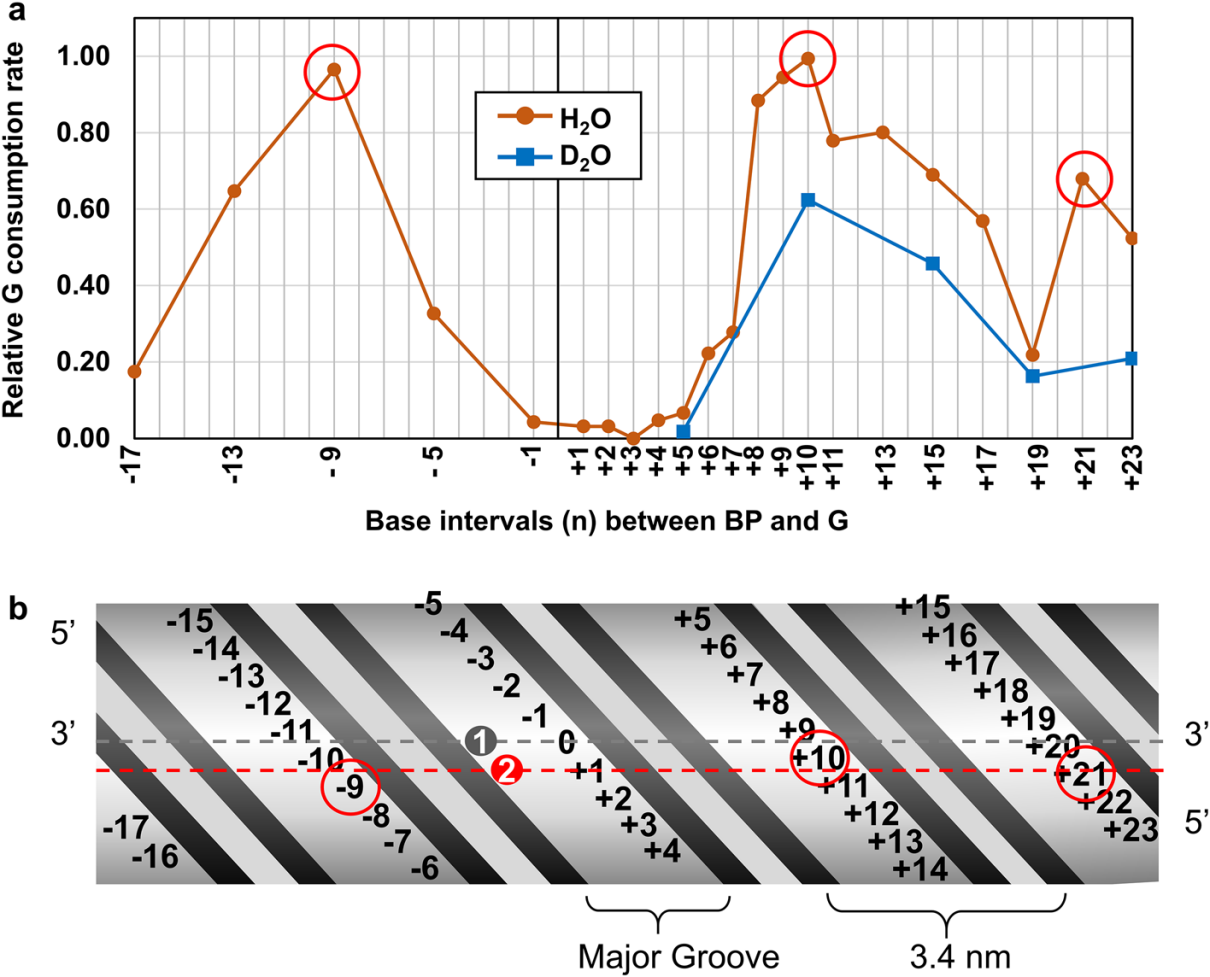
(**a**) The relative G consumption rate profile as the function of base intervals (n) in the photooxidation of dsDNA1[n], dsDNA2[n], and dsDNA3[n] in H_2_O and D_2_O. The red open circles indicate the hot spots of guanine photooxidation, where the oxidation efficiencies were locally maxima. (**b**) A simplified unfolded diagram of the cylindrical surface formed by the exterior of a B-DNA duplex with BP photosensitizer. The top and bottom sides of the diagram are formed by cutting the side opposite to BP in the axial direction of B-DNA cylinder. Grey point 1 and red point 2 are the locations of BP center respectively without and with consideration of the biased dislocation.

The relative G consumption in the 10-min photooxidation for 22 dsDNAs in H_2_O and for five dsDNAs in D_2_O and the corresponding ^O^G production in H_2_O are listed in Table 1. The relative G-consumption rate profile in the photooxidation of these dsDNAs as the function of the base interval n is shown in Fig. 4a. The profile shows three local maxima at n = − 9, + 10, and + 21, which we refer to as hot spots in the following discussions.

In the short distance region (n = − 1 to + 5) of the G consumption profile, almost no reactions were observed despite the close proximities of G and BP, which was also evident from no ^O^G productions in this region (Table 1). The inactivation within the short distance between G and BP could be explained either by quenching of the BP excited state^44,45^ by G, an accelerated collisional quenching of ^1^O_2_^46^ with G relative to the G-photooxidation rate or a steric barrier imposed by BP against the collision of ^1^O_2_ and G.

In the medium distance range (n = − 5 to − 9 and + 6 to + 8), the G consumption rate was drastically increased with increasing base intervals, probably because the inactivation described above is limited for the short range up to n =  ± 5. This drastic disappearance of inactivation effect as the function of distance is ascribable to a quenching by Dexter-type electron transfer from BP to G^47–51^. The absence of overlaps between the fluorescence emission of BP (*λ*_*max*_ = 551 nm) and absorption bands of G (*λ*_*max*_ = 254 nm) excludes the possibility of a Förster-type quenching by energy transfer. However, the other two possible inactivation mechanisms described above cannot be excluded at this point. The issue of inactivation mechanism is addressed later. The base intervals at the tallest peaks (n = − 9 and + 8-to- + 10) of G-consumption profile afforded the best yields of ^O^G (30 ~ 50%).

Since the relatively far away regions (n = − 9 to − 17 and + 10 to + 19) are free from the inactivation effect, the extent of G consumption therein should solely depend on the ^1^O_2_ concentration near G of interest as expressed by equation iii. As such, the gradual decreases in the G consumption rate with increasing G-BP separation should reflect the distance-dependent inactivation or dilution of ^1^O_2_. If the reaction retardation is related to an inactivation of ^1^O_2_, it should be due to quenching by solvent water, in which the half-lifetime of ^1^O_2_ is 3.5 μs^25^. We thus carried out the photooxidation of dsDNA1[n] in D_2_O, in which the half-lifetime of ^1^O_2_ (68 μs) is longer than that in H_2_O. As a result, the G-consumption rates for the photooxidation of dsDNA2(+ 10), dsDNA2(+ 15), and dsDNA3(+ 23) significantly decreased in D_2_O as shown in Fig. 4A. This result indicates that the main cause of decrease in the G consumption rate in the relatively long BP-G distance domain was not quenching but a diffusion-derived dilution of ^1^O_2_. The retardation of the photooxidation by D_2_O will be more deeply discussed later.

**Figure 5:**
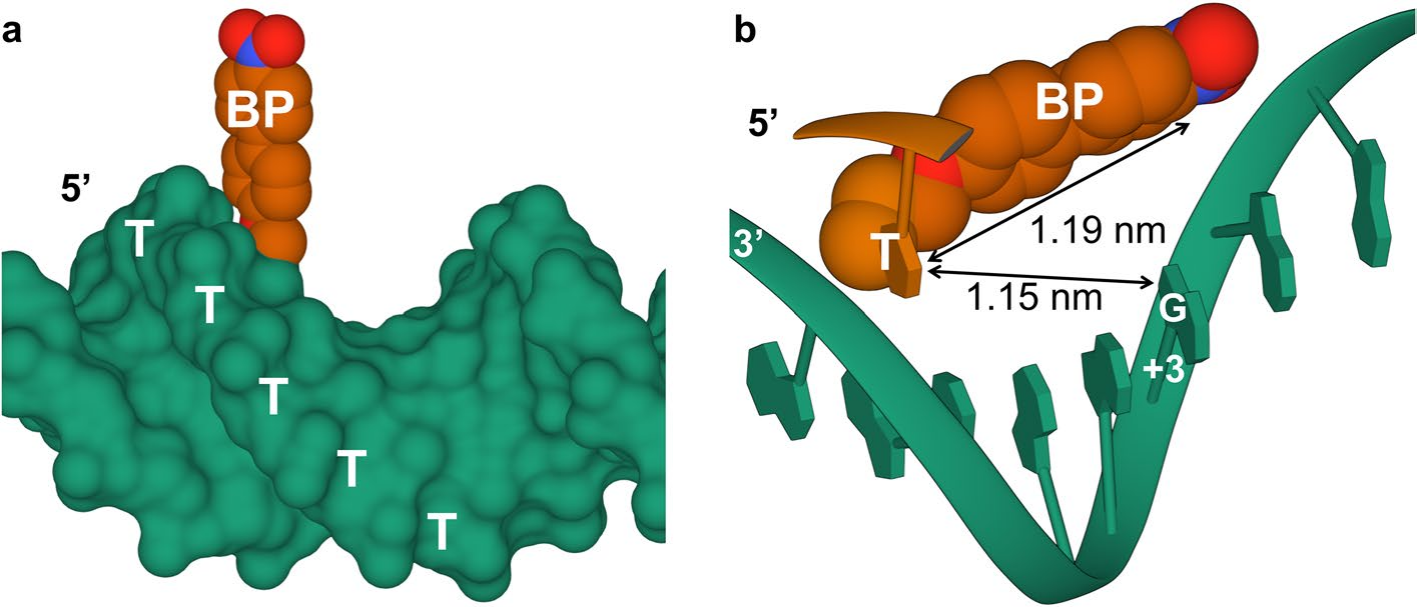
(**a**) Steric blocking of BP movement by the neighboring T wall at 5’ side. (**b**) A DNA duplex model highlighting the most accessible G (n =  + 3) for collision with the appended BP. The images of DNA models were created by Mol* Plugin 3.28.0 (https://molstar.org/).

Although the profile looks roughly M-shaped (Fig. 4a), it actually is asymmetric between the positive and negative regions of n, as exemplified by the difference of relative G consumption rate between n = − 5 (0.33) and + 5 (0.07). This asymmetry is probably because of a biased dislocation of BP toward 3’-end of attached ON owing to a wall-like blocking by the adjacent T at 5’-side as shown in Fig. 5a (the images of DNA models were created by Mol*^52^). This biased dislocation of BP might be reflected in the G consumption profile, in which the dislocated BP position (red point 2) is closer to the hot spots than the BP-appended base position (grey point 1) as indicated by broken lines (Fig. 4b). However, we cannot completely rule out the possibility of conformational distortion or fluctuation of the DNA duplexes as the cause of the asymmetry. The attenuated G consumption at n =  + 5 relative to n = − 5 is explainable by the enhanced inactivation by the closer BP.

Based on the above results, additional experiments were conducted to dispel related concerns, i.e., intermolecular crosstalk photooxidation and radical photooxidation by type I mechanism.

The intermolecular photooxidation was studied with dsDNA5[+ 10] and dsDNA6, in which the photosensitizer BP and reactant G were incorporated independently in dsDNA5[+ 10] and dsDNA6, respectively. The photoirradiation of the mixture of dsDNA5[+ 10] and dsDNA6 under the same conditions as those for the above photooxidation resulted in the consumption of G in dsDNA6 by 7% (Supplementary, Fig. S49). The extent of cross-talk was small and does not affect the interpretation of the above intramolecular photooxidations.

Nonetheless, it still is intriguing that the intermolecular photooxidation is slightly more efficient than the intramolecular ones for the dsDNAs with n = − 1 to + 4. This exceptional nonreactivity at the short G-BP distances is ascribable to the quenching of BP by G through a Dexter-type electron transfer as described above, but we cannot completely abandon the possibilities of accelerated ^1^O_2_ quenching by a physical contact with G^46^ and steric hindrance by BP. We thus devised to quantify the leaked ^1^O_2_ during photooxidation of dsDNA2[n] by using 100 μM of furfuryl alcohol (FA), a ^1^O_2_-trapping agent^53,54^, which is 50-times larger in concentration than dsDNA. The remained FA was analyzed by HPLC with absorbance at 216 nm (see Supplementary for details).

**Figure 6:**
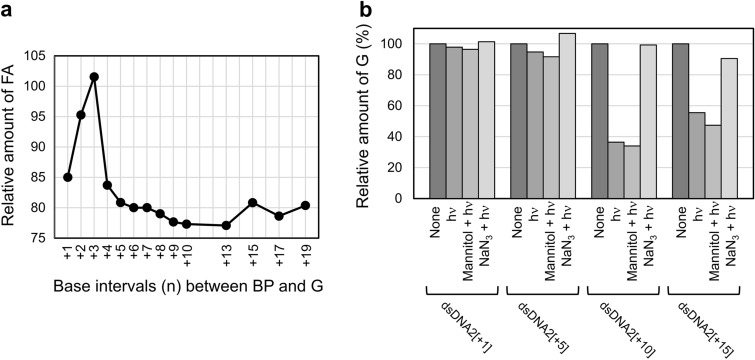
(**a**) Analysis of the amount of leaked ^1^O_2_ in the photooxidation of dsDNA2[n] by quantifying the amount of remaining furfuryl alcohol (FA), a ^1^O_2_ scavenger. (**b**) The effects of mannitol and sodium azide on the photooxidation of dsDNA2[n]s.

As shown in Fig. 6a (Table S3), we observed a sharp rise of the remained FA from n =  + 1 to + 3 and then a drop followed by a long tail indicating 15–23%-consumption of FA between n =  + 4 and + 19. The consumption of about 20% is surprisingly high in consideration of the presence of only 0.02 equivalent of the BP photosensitizer. This result indicates that BP has a substantial turnover number as a photosensitizer with negligible photobleaching. This efficient photooxidation of FA by ^1^O_2_ can be explained by its 20-times higher rate constant (*k*_*rOF*_ = 1 × 10^8^ M^− 1^ s^− 1^)^54^ over that for total quenching rate of ^1^O_2_ by G (*k*_*tOG*_ = 5 × 10^6^ M^− 1^ s^− 1^)^55^. The significantly large *k*_*rOF*_/*k*_*tOG*_ value (20-fold) does not match a hypothesis that almost all the ^1^O_2_ molecules produced from the short interval dsDNA (n =  + 3) were quenched by G and thus could not react with FA. BP's steric hindrance against ^1^O_2_-G collision, if any, would give an inversed FA residue profile, because ^1^O_2_ would be most abundant at n =  + 3 owing to no consumption by G. It is thus more adequate to explain the non-reactivity at n =  + 3 by direct collisional quenching of BP. At n =  + 3, the distance between G and BP-appended T is about 1.15 nm, which is very close to the length (1.19 nm) between the end of propyl linker and the carbon atom next to the nitro group in BP (Fig. 5b). Hence, the Dexter-type quenching by collision between BP and G should have culminated at this position, giving the peak in the FA residue profile.

In order to investigate a possibility that type I mechanism might partially contribute to the photooxidation of G by BP, the photooxidation of dsDNA2[n] was conducted in the presence of either a type I inhibitor, mannitol, or a type II inhibitor, sodium azide (Fig. 6b, Fig. S50)^56–58^. As a result, photooxidation of G was inhibited significantly by sodium azide, but not at all by mannitol as obviously observed for the photooxidation of dsDNA2[+ 10]. These results indicate that the photooxidation of G by BP proceeds exclusively through type II mechanism, which is consistent with its high quantum yield of ^1^O_2_ production (*Φ*_Δ_: 0.88) for a BP derivative^36,37^. The type-II exclusiveness is suitable for a selective photooxidation of ONs, since type I oxidation would be accompanied by remote G oxidation as described above.

## Discussion

For the purpose of pursuing good efficiency of G photooxidation by the BP-ON conjugates, we can conclude that the optimum BP positions should be 8 to 10 bases away from the target G on the complementary DNA. The optimum lengths between the BP and G in the duplexes elucidated in this study will be indispensable in designing photooxidation-based oligonucleotide therapeutics.

In the nearer positions with 1 to 5 base intervals, the reaction efficiencies were near zero, which was attributed to a quenching of BP by G through the Dexter mechanism^47–51^. The retardation of the photooxidation rate in the further region with more than 11 bases separation is believed to be due to dilution of ^1^O_2_. There could have two mechanisms to interpret the ^1^O_2_ dilution at the distant reaction points: ^1^O_2_ quenching by H_2_O and/or diffusion-derived dilution. Here, we delve into the extent of the contributions of these two possible mechanisms.

First of all, the reaction rate of ^1^O_2_ with G is extremely small compared to the rate of its quenching by water. According to the literatures, the first-order quenching rate constant (*k*_*qW*_) of ^1^O_2_ with water is 2.4–3.2 × 10^5^ (s^-1^)^59^ and the second-order total quenching rate constant (*k*_*tOG*_) of ^1^O_2_ with G is 5 × 10^6^ (M^-1^ s^-1^)^55^. Considering the initial concentration of G ([G]_0_ = 2 × 10^− 6^ M) used in this study, the apparent first-order decay constant (*k*_*qG*_) for the quenching of ^1^O_2_ by G is *k*_*qG*_ = *k*_*tOG*_[G] ≤ 10 (s^-1^), which is overwhelmingly smaller than *k*_*qW*_ (*k*_*qG*_/*k*_*qW*_ < 3.6 × 10^− 5^). Furthermore, in a study with a G derivative, the total ^1^O_2_ quenching rate (*k*_*tOG*_) by G was classified into those by physical collision (*k*_*qOG*_) and chemical reaction (*k*_*rOG*_), the quenching rate of the former being about 45 times that of the latter (*k*_*qOG*_/*k*_*rOG*_ ≈ 45)^46^. This means that the probability of the reaction of ^1^O_2_ with G occurring is 0.85 ppm compared to the quenching by H_2_O. Even with such a low probability, we were able to observe the progress of G photooxidation thanks to the relatively long reaction period of 10 min.

Even with the apparently high quenching rate with H_2_O, the average travelling length of ^1^O_2_ in H_2_O within its half-lifetime (3.5 μs)^24^ is calculated as 205 nm from Einstein’s diffusion theory^60,61^, which is much larger than 7 nm, the furthest BP-G length for a significant photooxidation occurrence (Fig. 4b). This means that the poor ^1^O_2_-oxidation reactivities of dsDNAs with a BP-G length of about 7 nm at n =  + 19 cannot be explained only by the quenching of ^1^O_2_ by H_2_O. Therefore, we suggest that it simply depends on the three-dimensional diffusion-derived dilution of ^1^O_2_. Implication of three-dimensional diffusion in the distance-dependent reaction rates in a ^1^O_2_ oxidation of a reactant has been pointed out by the other studies^62,63^.

The diffusion-derived dilution of ^1^O_2_ as the main cause of the retarded G photooxidation at 7-nm BP-G distance is also supported by D_2_O solvent effect as described above (Fig. 4a). Here we discuss how D_2_O retarded the G photooxidation. The rate-determining step for the ^1^O_2_-oxidation of G has been suggested to be the nucleophilic attack of ^1^O_2_ at C8 of G, which requires no protonation or deprotonation^64^. Hence, it is unlikely that solvent isotope effect was in play for the retardation of G oxidation in D_2_O. On the other hand, the oxidation of G is suggested to be a non-diffusion-controlled process as is the case for that of many biomolecules, in which the reaction is much slower than the diffusion of substaretes^65^. In this case, the apparent reaction rates would be hardly influenced by viscosity of D_2_O^66^. As to this solvent effect issue, it is intriguing to note that the hydration water near DNA surface has been found to diffuse as rapidly as the bulk water^67^. This phenomenon is peculiar to DNA duplexes and has never been observed for the other biomolecules such as proteins and lipid membranes, in which their surfaces are more strongly hydrated than bulk water and enthalpically protected from binding by the other molecules. As such, ^1^O_2_ would be able to travel just above the DNA surface as easily as in the bulk water. There is a possibility that D_2_O does not have this special weak hydration phenomenon, thus having inhibited the ^1^O_2_ diffusion along DNA. The stronger hydration of biomolecules^68,69^ and DNA^70^ by D_2_O than H_2_O has been pointed out in previous studies.

From the efficient turnover of the photooxidation with BP, it is presumed that ^1^O_2_ was generated at a nearly constant rate during 10-min light irradiation and [^1^O_2_] is kept constant at each G reaction site. This is supported by the observed pseudo-first order kinetics with [^1^O_2_] kept constant, in which the rates of disappearance of G and production of ^O^G were linear within 10 min. In other words, the diffusion of ^1^O_2_ is in a steady state and [^1^O_2_] should be constant at a certain place and depends only on the distance from BP. In a spherical coordinate model for a steady-state diffusion, molecules of interest are continuously generated at a point and the concentration of the solute at a certain point (*r*) away from the generation point is always proportional to 1/*r*^71^. Unlike such an ideal space, this study deals with a ^1^O_2_ solute whose generating point, BP, is attached on the side of a cylindrical matter, dsDNA. Therefore, G located on the back side of the cylinder across from BP is most likely accessible only by going around the cylinder despite the shorter direct distance in the spherical coordinates. This wrapping diffusion of ^1^O_2_ around a cylindrical DNA surface seems to be realized in the fluctuation cycle of G consumption profile that coincides with the pitch of 10.5 base pairs per turn in B-DNA (Fig. 4a).

It should be noted here that poly(dA):poly(dT) double strands with typical CD spectra shown in Fig. 2B have been reported to have a smaller pitch of 10 base pairs per turn in a so-called B'-DNA^72,73^. The pitch difference from B-DNA is small but the B'-conformation fail to explain the relatively low G photooxidation efficiencies at the second cycle positions (n = − 17 and + 19). One possible explanation for this anomality if we assume the B'-conformation is that the TA base pairs at the both ends might be flaying, thereby the Gs near the ends shifting the locations from the canonical conformation.

The fluctuated G consumption profile also suggests that penetration through the major groove is not at least the main travelling route of ^1^O_2_. The major groove water has been reported to have about twice smaller diffusion coefficient than that of the bulk water^74,75^, probably owing to its constrained environment. Therefore, a retarded diffusion and accelerated quenching owing to a constrained H_2_O in the major groove might have worked against ^1^O_2_ taking this path as the main diffusion route.

Diffusion of ^1^O_2_ along a DNA duplex can be generalized as the diffusion of particles on the surface of a cylinder. Diffusion of particles on a cylindrical surface has been described to go through the combination of adsorption, crawling, desorption, bulk diffusion, and rebinding^38^. The desorbed particles have to travel a much longer distance before rebinding than that can be reached by the crawling particles in the same timescale. If we assume 3.5 μs for an average travelling time (205 nm in length) of the desorbed ^1^O_2_ molecules in the bulk water, half of them would be inactivated into ^3^O_2_ when rebound to the DNA surface. In this study, we demonstrated that quenching of ^1^O_2_ by H_2_O was negligible in the G consumption profile, which suggests that the desorbed ^1^O_2_ molecules had little effect on the ^1^O_2_ concentration mapping along DNA. The characteristic BP-G length dependence of G photooxidation in Fig. 4a is thus suggested to be the result of average ^1^O_2_ diffusion paths mainly via surface crawling and/or flying just above the surface by taking as short a distance as possible before inactivation into ^3^O_2_. However, care must be taken for the diffusion model because DNA structure is constantly fluctuating and thus may not be an ideal cylinder model. Since we have not directly observed ^1^O_2_ for surface diffusion, we must examine the diffusion mechanism more deeply, for example, by studying the effects of salt and temperature.

In conclusion, it was found that the optimal attaching sites of the BP photosensitizer in an oligonucleotide (ON) to photooxidize G in the complementary strand was a position separated from G by 8–10 bases. This information will be very important in the development of nucleic acid-targeting PDT. For application, the BP-appended ONs should contain inosine instead of G to avoid self-photooxidation. Also, nuclease-resistant ON analogues with bioisostere linkages such as peptide nucleic acid (PNA) should be employed. PNA could be used to target dsDNA. dsDNA could also be targeted by binding BP to a triplex-forming oligonucleotide that fits in the major groove of dsDNA. Apart from application, this study evaluated the distribution of ^1^O_2_ concentration along DNA due to diffusion from continuous sources of ^1^O_2_. This information can be valuable data that traces how molecules emanating from the surface of a nano-size cylinder diffuse to its back side.

## Supplementary Information

Supplementary Information.

## Data Availability

The datasets used or analysed during the current study available from the corresponding author on reasonable request.
