# Supplementary material for: Mapping the diffusion pattern of 1O2 along DNA duplex by guanine photooxidation with an appended biphenyl photosensitizer
Source: Sci Rep. 2023 Jan 23;13:288. doi: 10.1038/s41598-023-27526-2 (PMC9871026; doi:10.1038/s41598-023-27526-2)
Supplement: Supplementary file 1 — Supplementary Information. [file 41598_2023_27526_MOESM1_ESM.pdf]

Supplementary Information

Mapping the diffusion pattern of $^1O_2$ along DNA duplex by guanine photooxidation with an appended biphenyl photosensitizer

Takashi Kanamori*, Shota Kaneko, Koji Hamamoto, Hideya Yuasa*

School of Life Science and Technology, Tokyo Institute of Technology, J2-10 4259 Nagatsuta, Midoriku, Yokohama 226-8501, Japan

Table of contents

1. Synthesis -- S3~S48

General methods -- S3

Synthetic protocols, ^{1}H NMR, ^{13}C NMR, and ESI-TOF-MS spectra -------- S4~S21

Oligonucleotide synthesis, purification, and sequence list ---------------------- S22~S23

RP-HPLC charts, and ESI-TOF-MS spectra ------------------------------------ S24~48

2. Measurement of singlet oxygen generation ability of BP-T------------------------------ S49

3. UV-melting temperatures measurements --S50~S51

4. CD spectra measurements -- S52

5. Photooxidation, enzymatic digestion, and HPLC analysis of ONs -------------------- S52~S57

6. Gel mobility shift analysis of photooxidized ONs --- S58

7. $^{1}O_2$ production analysis of BP-modified ONs by furfuryl alcohol --------------------- S58

8. Gel electrophoresis analysis of the photooxidation products --------------------------- S59

9. Photooxidation of ONs in the presence of NaN_3 or mannitol -------------------------- S60~S61

10. References -- S61

1. Synthesis

General methods: All reagents and starting materials were purchased from Wako Pure Chemical Industries, Ltd.; Tokyo Chemical Industry Co., Ltd.; Kanto Chemical Co., Inc.; Sigma Aldrich Co., Nacalai Tesque, Inc. Water used for the organic synthesis was purified by Milli-, Merck Millipore. For the spectroscopic measurement, spectroscopic analysis grade of solvents from Wako Pure Chemical Industries, Ltd. were used. Thin-layer chromatography (TLC) was performed on pre-coated silica gel Merck 60-F_{254} plates and visualized by the UV light (AS ONE SLUV-6, 254 or 365 nm) or charring after immersing in a solution of 1% $Ce(SO_4)_2$·1.5% $(NH_4)_6Mo_7O_{24}\cdot 4H_2O$ in 10% H_2SO_4. Column chromatography was performed on Wako gel C-200, Kanto silica gel 60N (spherical, neutral) or Biotage Sfär HC D, or Fuji Silysia NH silica gel (NH-DM1020) with the solvent system specified. ^{1}H NMR spectra were recorded at 400 MHz (JEOL JNM-ECZL400S) or 500 MHz (Varian Unity INOVA 500 or Bruker AVANCEIII HD 500). The tetramethylsilane peak (δ = 0.00 ppm) or a solvent peak was used as a standard in $CDCl_3$ (δ = 7.26 ppm), CD_3OD (δ = 3.31 ppm) or $(CD_3)_2SO$ (δ = 2.50 ppm). Chemical shifts are expressed in ppm referenced to the standards. The multiplicity of the signals is abbreviated as follows: s = singlet, d = doublet, dd = doublet of doublets, t = triplet, quin = quintet, br = broad signal, and m = multiplet. ^{13}C NMR spectra were recorded at 126 MHz (Varian Unity INOVA 500 or Bruker AVANCEIII HD 500) and a solvent peak of $CDCl_3$ (δ = 77.16 ppm), CD_3OD (δ = 49.00 ppm) or $(CD_3)_2SO$ (δ = 39.52 ppm) was used as a standard. High-resolution mass spectra (HRMS) were recorded on a Bruker micrOTOF II ESI-TOF MS.

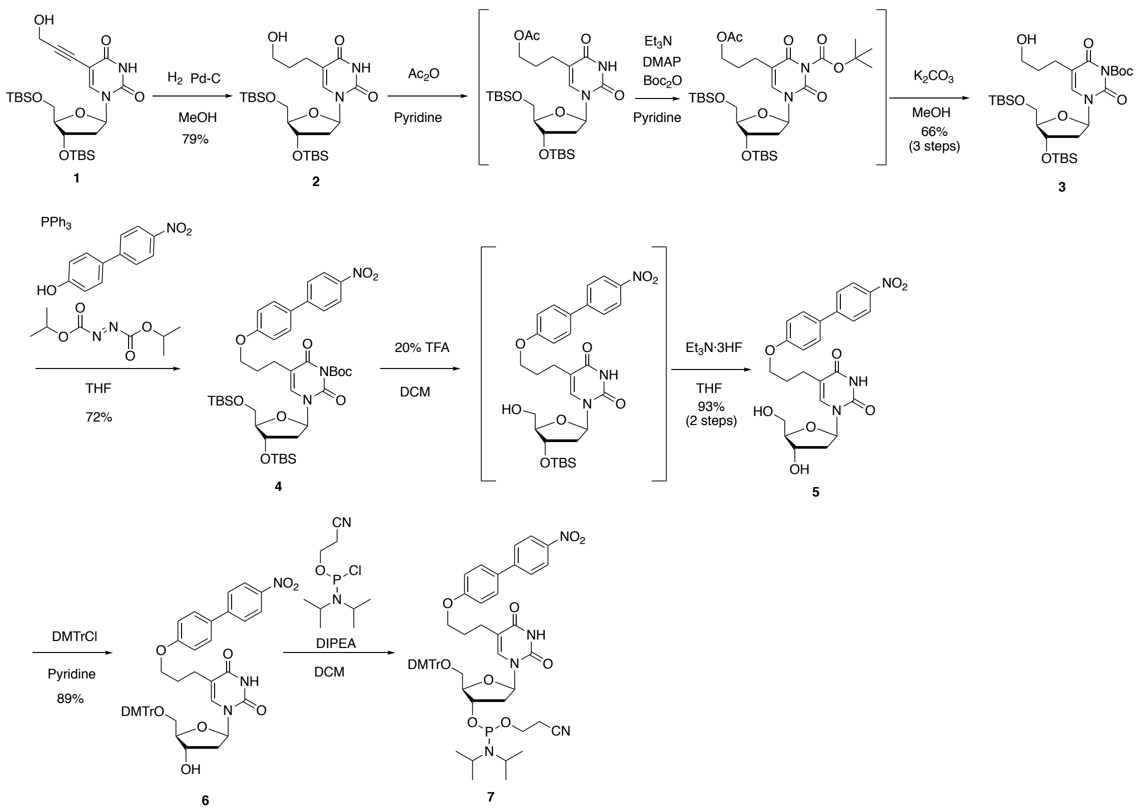

Fig. S1 Synthetic scheme for phosphoramidite unit (**7**) of the photosensitizer-appended thymidine.

5-(3-Hydroxypropane-1-yl)-3',5'-*O*-bis(*tert*-butyldimethylsilyl)-2'-deoxyuridine (2)

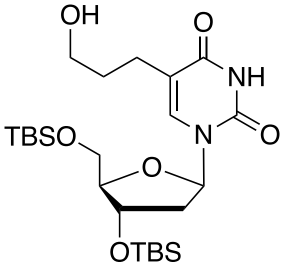

To a solution of compound **1**[1] (1.95 g, 3.82 mmol) in methanol (100 mL), was added 10% Pd-C (898 mg) under nitrogen atmosphere. Then, the nitrogen gas was replaced with hydrogen gas. The reaction mixture was stirred vigorously at room temperature for 22.5 h. The reaction mixture was filtered by celite and the filtrate was evaporated. The residue was purified by column chromatography (60N gel, hexane-EtOAc, 3:2, v/v) to give compound **2** (1.54 g, 79%) as syrup. R_f 0.44 (hexane/EtOAc (1:1 v/v)); ^{1}H NMR (500 MHz, $CDCl_3$) δ 9.09 (s, 1H, NH), 7.49 (s, 1H, H-6), 6.32 (dd, J = 5.6 Hz, J = 8.2 Hz, 1H, H-1'), 4.39 (br, 1H, H-3'), 3.93 (d, J = 2.6 Hz, 1H, H-4'), 3.84 (dd, J = 2.9 Hz, J = 11.4 Hz, 1H, H-5'), 3.75 (dd, J = 2.8 Hz, J = 11.4 Hz, 1H, H-5'), 3.59 (br, 2H, -$CH_2CH_2\underline{H_2}$-O-), 2.65 (br, 1H), 2.36-2.51 (m, 2H, -$C\underline{H_2}CH_2CH_2$-O-), 2.25 (dd, J = 3.3 Hz, J = 13.2 Hz, 1H, H-2'), 1.94–2.03 (m, 1H, H-2'), 1.66-1.77 (m, 2H, -$CH_2C\underline{H_2}CH_2$-O-), 0.92 (s, 9H, *t*-Bu-Si), 0.89 (s, 9H, *t*-Bu-Si), 0.10 (s, 6H, Me-Si x2), 0.074 (s, 3H, Me-Si), 0.067 (s, 3H, Me-Si); ^{13}C NMR (126 MHz, $CDCl_3$) δ 164.55, 150.32, 136.65, 114.57, 87.98, 85.05, 72.41, 63.18, 61.05, 41.44, 32.65, 26.06, 25.87, 23.14, 18.52, 18.12, -4.53, -4.72, -5.27, -5.30; ESI-TOF-MS calcd for $C_{24}H_{46}N_2NaO_6Si_2^+$ [M+Na]$^+$ 537.2787; found 537.2798.

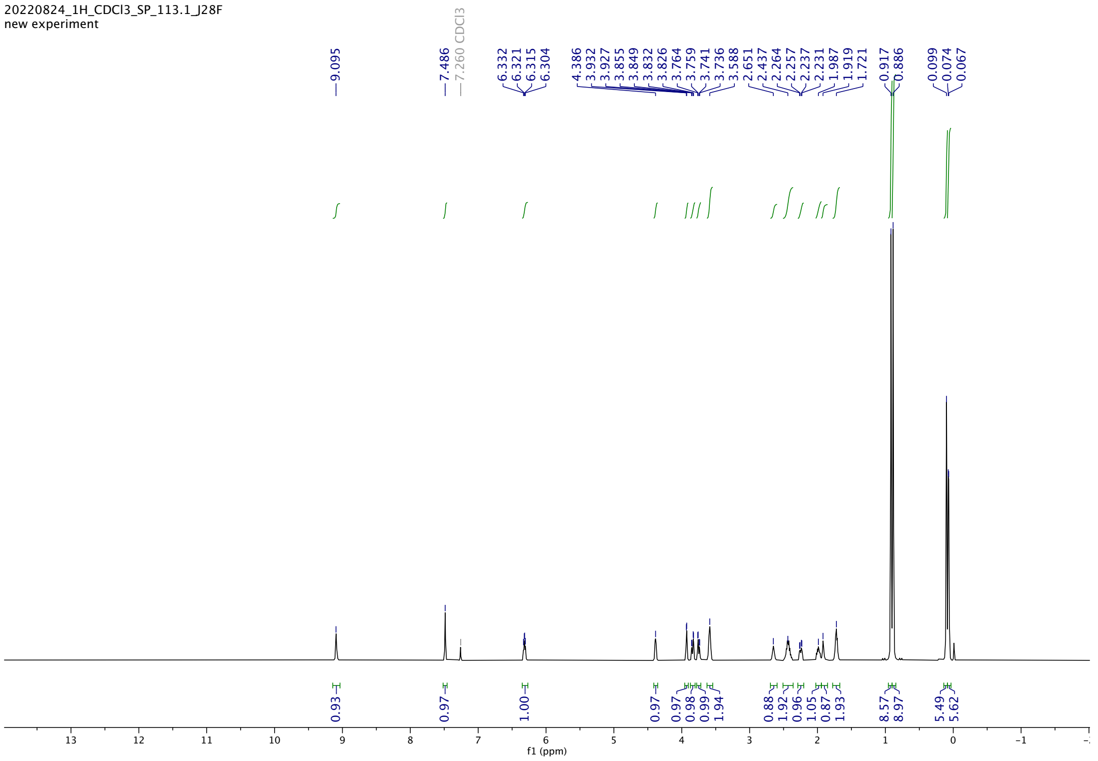

Fig. S2 ^{1}H-NMR spectrum of compound **2**.

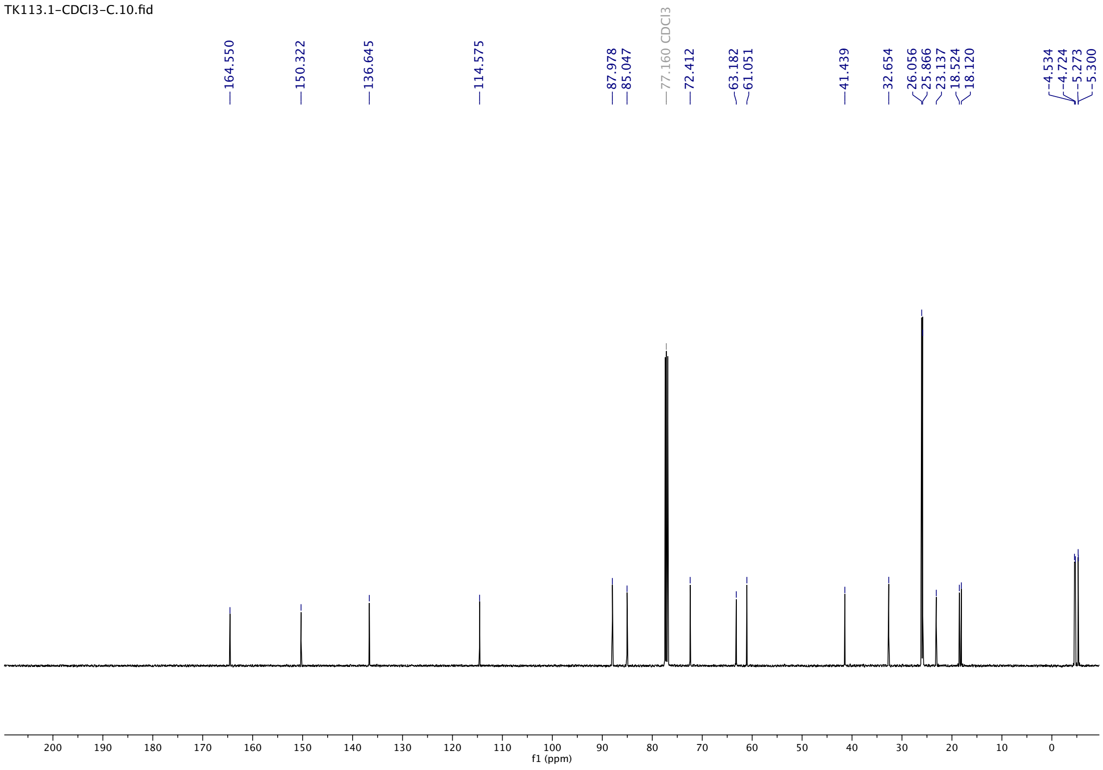

Fig. S2 ^{13}C-NMR spectrum of compound **2**.

TDCMAS ESI-TOF

Analysis Info

Analysis Name	D:\Data\yuasa_lab\kanamori\211214\SP113-1-000001.d	Acquisition Date	2021/12/13 13:08:50
Method	esi_posi_low.m	Operator	BDAL@DE
Sample Name	SP113-1-	Instrument / Ser#	micrOTOF 213750.10321
Comment			

Acquisition Parameter

Source Type	ESI	Ion Polarity	Positive	Set Nebulizer	0.3 Bar
Focus	Not active			Set Dry Heater	180 ‹C
Scan Begin	50 m/z	Set Capillary	4500 V	Set Dry Gas	4.0 l/min
Scan End	1400 m/z	Set End Plate Offset	-500 V	Set Divert Valve	Waste

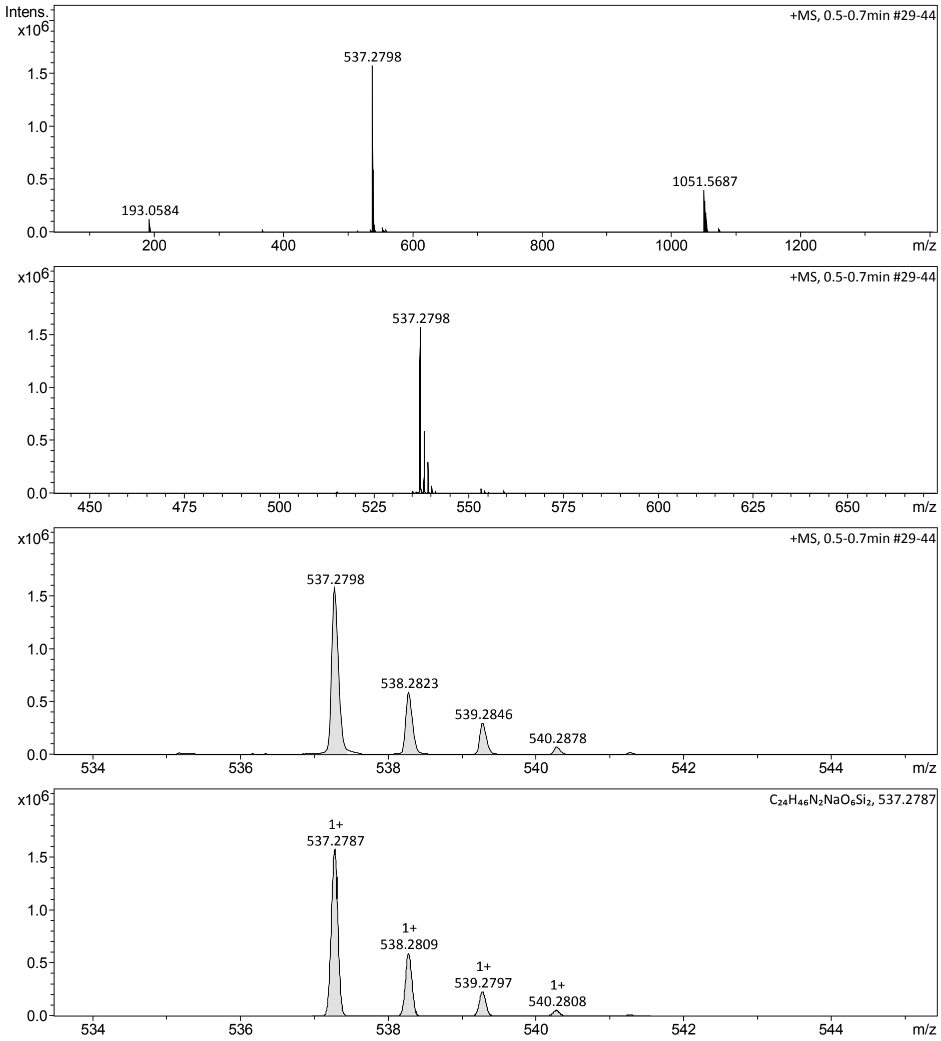

Fig. S4 ESI-TOF-MS spectra of compound **2**.

3-*N*-*tert*-Butoxycarbonyl-5-(3-hydroxypropane-1-yl)-3',5'-*O*-bis(*tert*-butyldimethylsilyl)-2'-deoxyuridine (3)

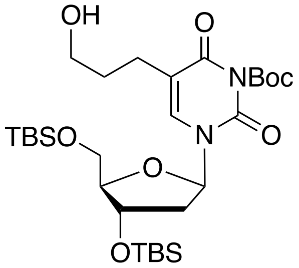

To a solution of compound **2** (1.04 g, 2.02 mmol) in pyridine (10 mL), was added Ac_2O (10 mL, 105.8 mmol). The reaction mixture was stirred at room temperature for 14.5 h. Then, it was evaporated and the residue was co-evaporated with toluene. To this residue, were added dry pyridine (10 mL), Boc_2O (882 mg, 4.04 mmol), Et_3N (844 µL, 6.06 mmol), and DMAP (123 mg, 1.01 mmol). The reaction mixture was stirred at room temperature for 4 h. Then the reaction mixture was diluted with MeOH and evaporated. The residue was dissolved in MeOH (10 mL). Then, to the solution was added K_2CO_3 (334 mg, 2.42 mmol). The reaction mixture was stirred at room temperature for 2 h. Then it was evaporated and the residue was purified by column chromatography (60N gel, hexane-EtOAc, 4:1, v/v) to give compound **3** (818 mg, 66%) as white amorphous solid. R_f 0.23 (hexane/EtOAc (3:1 v/v)); ^{1}H NMR (500 MHz, $CDCl_3$) δ 7.49 (s, 1H, H-6), 6.29 (dd, J = 5.6 Hz, J = 8.2 Hz, 1H, H-1'), 4.35–4.39 (m, 1H, H-3'), 3.92-3.96 (m, 1H, H-4'), 3.83 (dd, J = 2.9 Hz, J = 11.3 Hz, 1H, H-5'), 3.75 (dd, J = 2.7 Hz, J = 11.3 Hz, 1H, H-5'), 3.56–3.64 (br, 2H, -$CH_2CH_2C\underline{H_2}$-O-), 2.36–2.50 (m, 2H, -$C\underline{H_2}CH_2CH_2$-O-), 2.26 (ddd, J = 2.4 Hz, J = 5.6 Hz, J = 13.1 Hz, 1H, H-2'), 2.13–2.22 (br, 1H, OH), 1.94–2.02 (m, 1H, H-2'), 1.67–1.80 (m, 2H, -$CH_2C\underline{H_2}CH_2$-O-), 1.60 (s, 9H, *t*-Bu- (Boc)), 0.92 (s, 9H, *t*-Bu-Si), 0.88 (s, 9H, *t*-Bu-Si), 0.102 (s, 3H, Me-Si), 0.099 (s, 3H, Me-Si), 0.065 (s, 3H, Me-Si), 0.060 (s, 3H, Me-Si); ^{13}C NMR (126 MHz, $CDCl_3$) δ 161.84, 148.45, 147.97, 135.86, 114.20, 88.16, 86.96, 85.49, 72.51, 63.25, 61.30, 41.55, 32.49, 27.55, 26.07, 25.85, 23.50, 18.54, 18.10, -4.53, -4.73, -5.26, -5.28; ESI-TOF-MS calcd for $C_{29}H_{54}N_2NaO_8Si_2^+$ $[M+Na]^+$ 637.3311; found 637.3319.

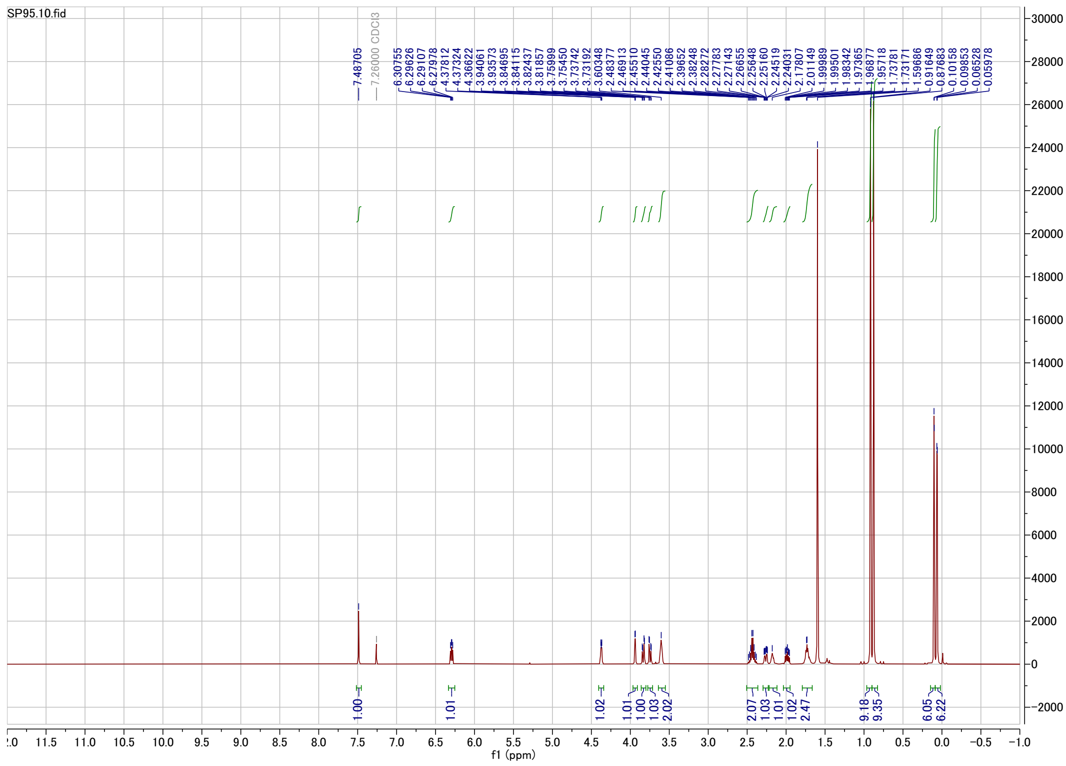

Fig. S5 ^{1}H-NMR spectrum of compound **3**.

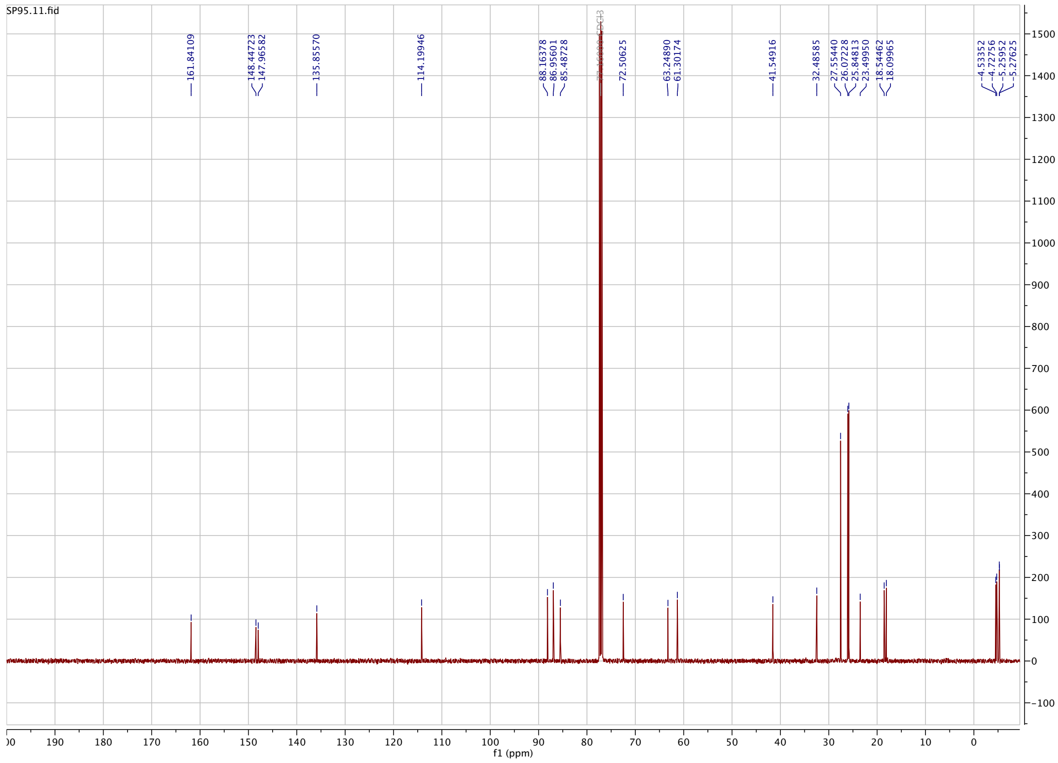

Fig. S6 ^{13}C-NMR spectrum of compound **3**.

TDCMAS ESI-TOF

Analysis Info

Analysis Name	D:\Data\yuasa_lab\kanamori\211214\SP95-000001.d	Acquisition Date	2021/12/13 13:21:40	
Method	esi_posi_low.m	Operator	BDAL@DE	
Sample Name	SP95-	Instrument / Ser#	micrOTOF	213750.10321
Comment				

Acquisition Parameter

Source Type	ESI	Ion Polarity	Positive	Set Nebulizer	0.3 Bar
Focus	Not active			Set Dry Heater	180 ‹C
Scan Begin	50 m/z	Set Capillary	4500 V	Set Dry Gas	4.0 l/min
Scan End	1500 m/z	Set End Plate Offset	-500 V	Set Divert Valve	Waste

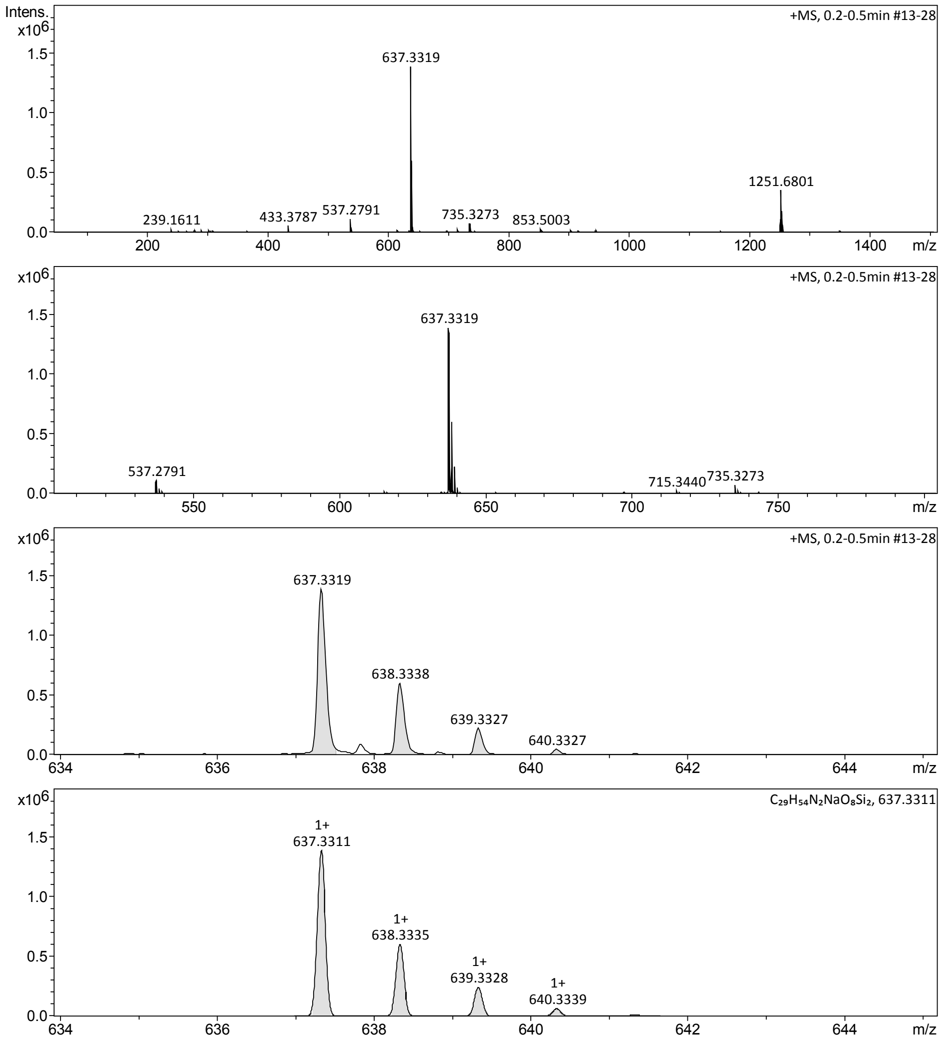

Bruker Compass DataAnalysis 4.2 printed: 2021/12/13 13:25:19 Page 1 of 2

Fig. S7 ESI-TOF-MS spectra of compound **3**.

3-*N*-*tert*-Butoxycarbonyl-5-(3-[(4'-Nitro[1,1'-biphenyl]-4-yl)oxy]propane-1-yl)-3',5'-*O*-bis(*tert*-butyldimethylsilyl)-2'-deoxyuridine (**4**)

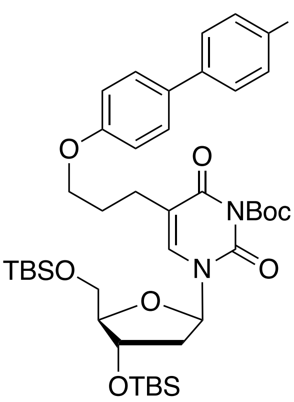

Compound **3** (500 mg, 0.813 mmol) was co-evaporated with dry toluene. To a solution of compound **3** in dry THF (4 mL), was added 4-(4-nitrophenyl)phenol (192 mg, 0.892 mmol) and PPh_3 (320 mg, 1.22 mmol). Then, to the reaction mixture was added DIAD (238 µL, 1.22 mmol) dropwise. The reaction mixture was stirred at room temperature for 2 h. It was diluted with CH_2Cl_2 and washed with water. The organic layer was dried over Na_2SO_4 and evaporated. The residue was purified by column chromatography (NH silica gel, hexane-EtOAc, 17:3, v/v) to give compound **4** (474 mg, 72%) as yellow amorphous solid. R_f 0.52 (hexane/EtOAc (3:1 v/v)); ^{1}H NMR (500 MHz, $CDCl_3$) δ 8.27 (d, J = 8.9 Hz, 2H, biphenyl), 7.69 (d, J = 8.9 Hz, 2H, biphenyl), 7.56 (d, J = 8.8 Hz, 2H, biphenyl), 7.49 (s, 1H, H-6), 6.99 (d, J = 8.8 Hz, 2H, biphenyl), 6.29 (dd, J = 5.6 Hz, J = 8.2 Hz, 1H, H-1'), 4.34–4.37 (m, 1H, H-3'), 4.04 (t, J = 6.0 Hz, 2H, -$CH_2CH_2C\underline{H_2}$-O-), 3.92–3.94 (m, 1H, H-4'), 3.80 (dd, J = 2.9 Hz, J = 11.4 Hz, 1H, H-5'), 3.73 (dd, J = 2.6 Hz, 11.4 Hz, 1H, H-5'), 2.48–2.59 (m, 2H, -$C\underline{H_2}CH_2CH_2$-O-), 2.24 (ddd, J = 2.1 Hz, J = 5.6 Hz, J = 13.1 Hz, 1H, H-2'), 2.02–2.10 (m, 2H, -$CH_2C\underline{H_2}CH_2$-O-), 1.90–1.97 (m, 1H, H-2'), 1.62 (s, 9H, *t*-Bu- (Boc)), 0.93 (s, 9H, *t*-Bu-Si), 0.87 (s, 9H, *t*-Bu-Si), 0.12 (s, 3H, Me-Si), 0.11 (s, 3H, Me-Si), 0.05 (s, 6H, Me-Si ×2); ^{13}C NMR (126 MHz, $CDCl_3$) δ 161.13, 160.00, 148.53, 148.11, 147.34, 146.68, 135.41, 131.15, 128.69, 127.17, 124.29, 115.25, 113.95, 88.17, 86.95, 85.51, 72.59, 67.55, 63.25, 41.51, 28.42, 27.58, 26.12, 25.85, 24.97, 18.56, 18.10, -4.54, -4.73, -5.16, -5.26; ESI-TOF-MS calcd for $C_{41}H_{61}N_3NaO_{10}Si_2^+$ $[M+Na]^+$ 834.3788; found 834.3783.

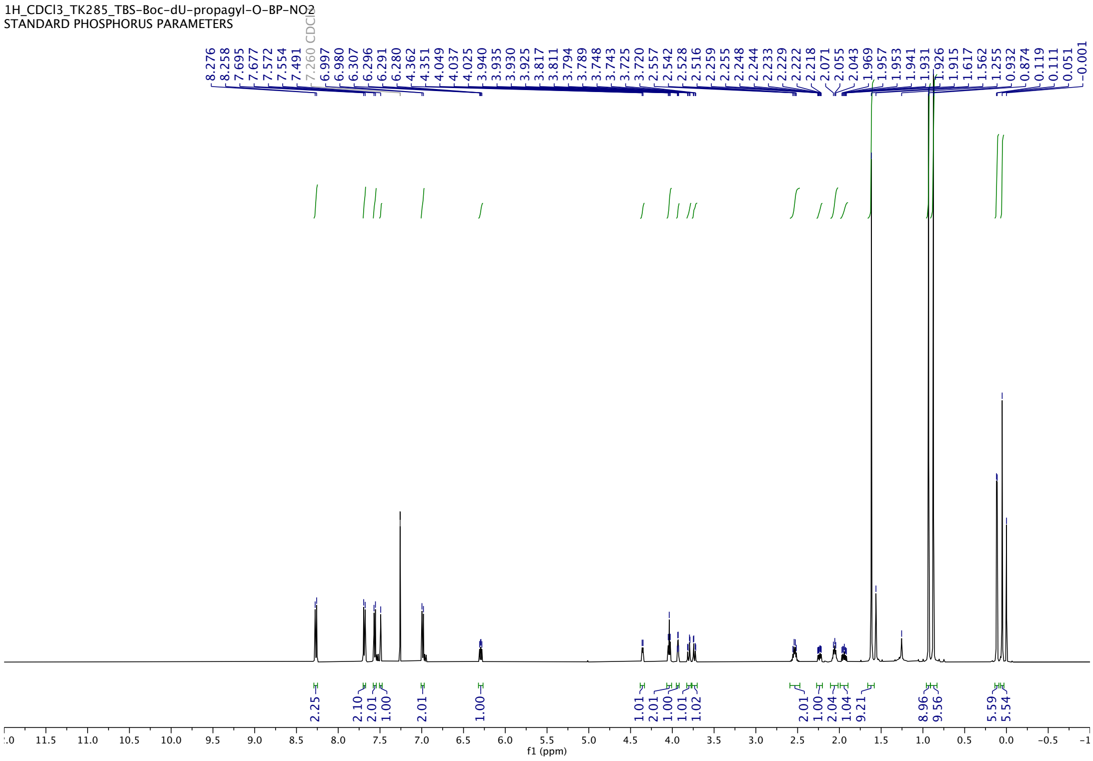

Fig. S8 ^{1}H-NMR spectrum of compound **4**.

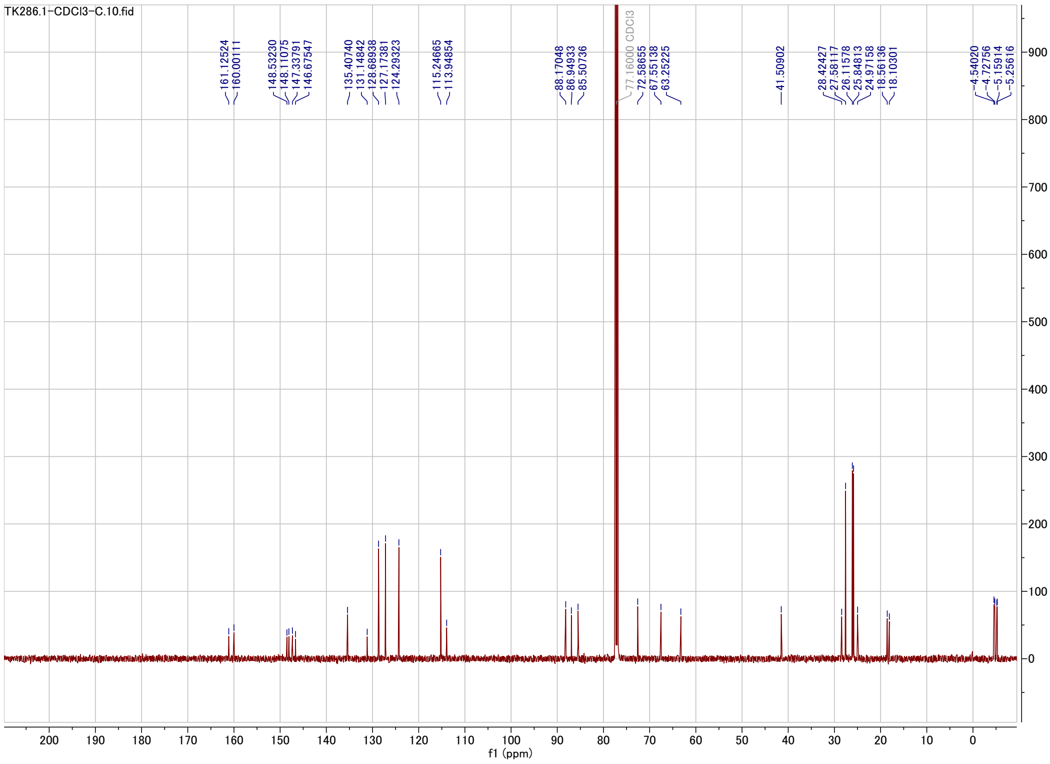

Fig. S9 ^{13}C-NMR spectrum of compound **4**.

TDCMAS ESI-TOF

Analysis Info

Analysis Name	D:\Data\yuasa_lab\kanamori\200115\TK286.1-000002.d	Acquisition Date	2020/01/14 11:49:58
Method	esi_posi_low.m	Operator	BDAL@DE
Sample Name	TK286.1-	Instrument / Ser#	micrOTOF 213750.10321
Comment			

Acquisition Parameter

Source Type	ESI	Ion Polarity	Positive	Set Nebulizer	0.3 Bar
Focus	Not active			Set Dry Heater	180 ‹C
Scan Begin	50 m/z	Set Capillary	4500 V	Set Dry Gas	4.0 l/min
Scan End	1900 m/z	Set End Plate Offset	-500 V	Set Divert Valve	Waste

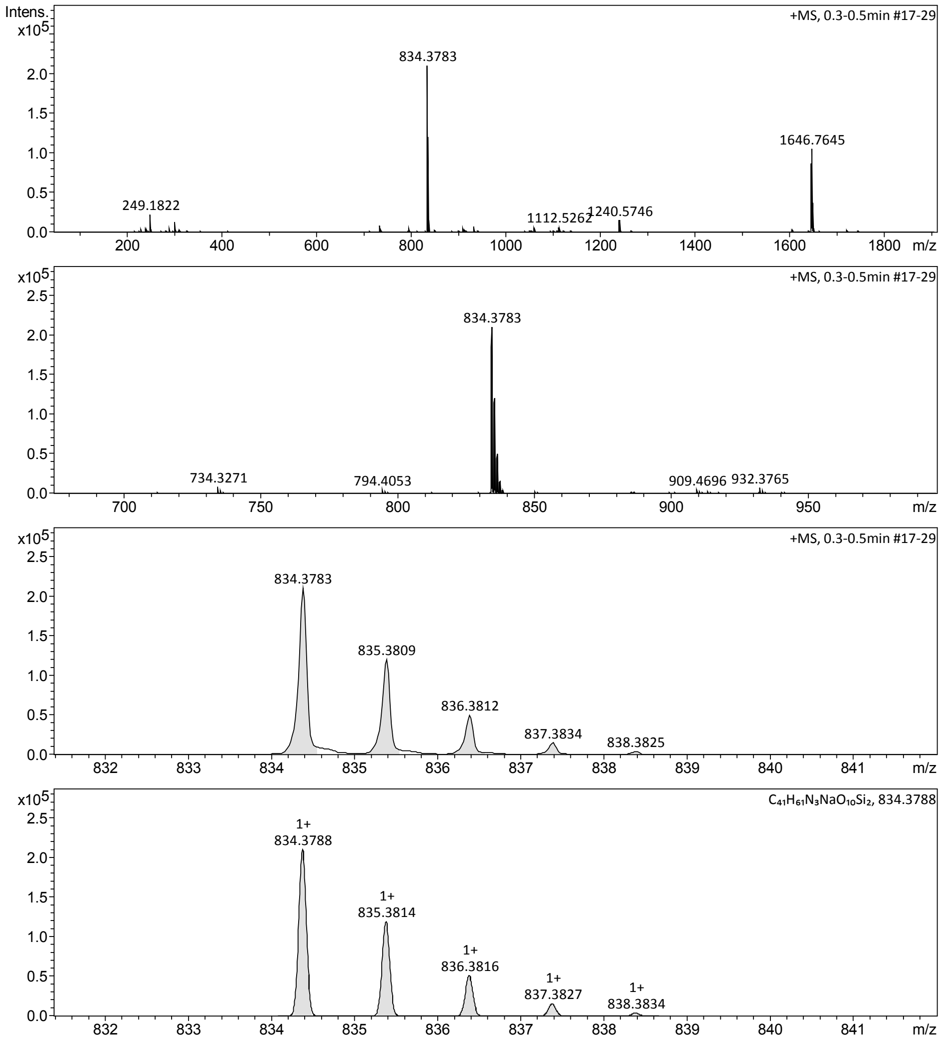

Fig. S10 ESI-TOF-MS spectra of compound **4**.

5-(3-[(4'-Nitro[1,1'-biphenyl]-4-yl)oxy]propane-1-yl)-2'-deoxyuridine (**5**)

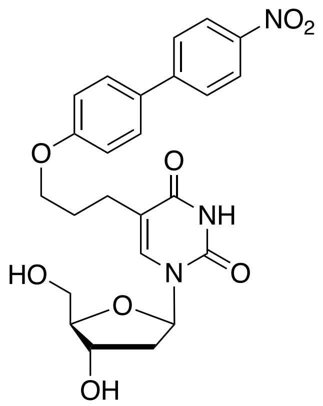

Compound **4** (350 mg, 0.431 mmol) was dissolved in 20% TFA in CH_2Cl_2 (20 mL) and stirred at room temperature for 1 h. The reaction mixture was evaporated and co-evaporated with toluene. To the solution of the residue in dry THF (5 mL), was added $Et_3N{\cdot}3HF$ (703 µL, 4.31 mmol). It was stirred at room temperature for 19 h. Then the reaction mixture was evaporated and co-evaporated with toluene 3 times. The residue was purified by column chromatography (60N gel, CH_2Cl_2-MeOH, 49:1, v/v). Then, to the solution of target compound in methanol (~1 mL), was added mix solvent (hexane-EtOAc, (2:1 v/v)) to give pure precipitate. It was dried to give compound **5** (194 mg, 93%) as yellow amorphous solid. R_f 0.29 (EtOAc); ^{1}H NMR (500 MHz, DMSO-d_6) δ 11.30 (s, 1H, N-H), 8.27 (d, J = 9.0 Hz, 2H, biphenyl), 7.93 (d, J = 9.0 Hz, 2H, biphenyl), 7.76 (d, J = 8.8 Hz, 2H, biphenyl), 7.73 (s, 1H, H-6), 7.08 (d, J = 8.8 Hz, 2H, biphenyl), 6.17 (t, J = 6.8 Hz, 1H, H-1'), 5.19-5.26 (br, 1H, OH), 5.02–5.08 br, 1H, OH), 4.20–4.24 (br, 1H, H-3'), 4.05 (t, J = 6.4 Hz, 2H, -$CH_2CH_2C\underline{H_2}$-O-), 3.74–3.78 (m, 1H, H-4'), 3.50–3.61 (m, 2H, H-5' x2), 2.34–2.44 (m, 2H, -$C\underline{H_2}CH_2CH_2$-O-), 2.02–2.08 (m, 2H, H2' x2), 1.93 (quin, J = 7.0 Hz, 2H, -$CH_2C\underline{H_2}CH_2$-O-); ^{13}C NMR (126 MHz, DMSO-d_6) δ 163.42, 159.58, 150.35, 146.33, 146.00, 136.39, 129.88, 128.58, 127.00, 124.11, 115.19, 112.79, 87.31, 83.90, 70.41, 67.14, 61.27, 27.29, 23.23; ESI-TOF-MS calcd for $C_{24}H_{25}N_3NaO_8^+$ $[M+Na]^+$ 506.1534; found 506.1522.

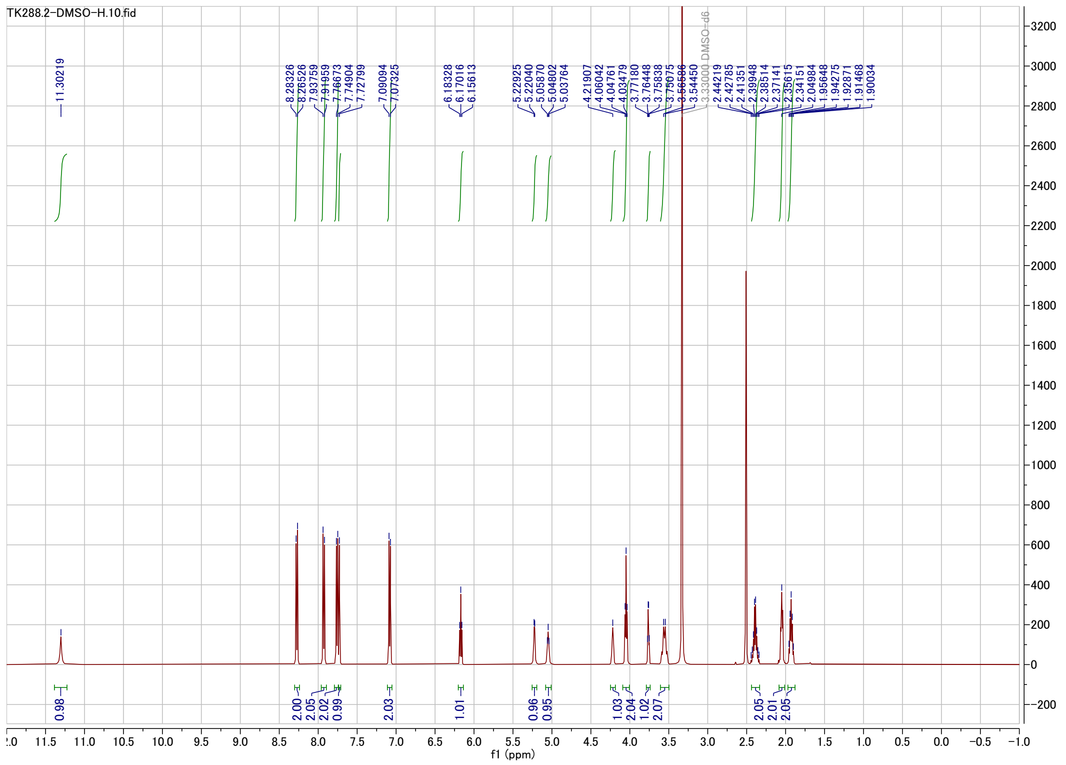

Fig. S11 ^{1}H-NMR spectrum of compound **5**.

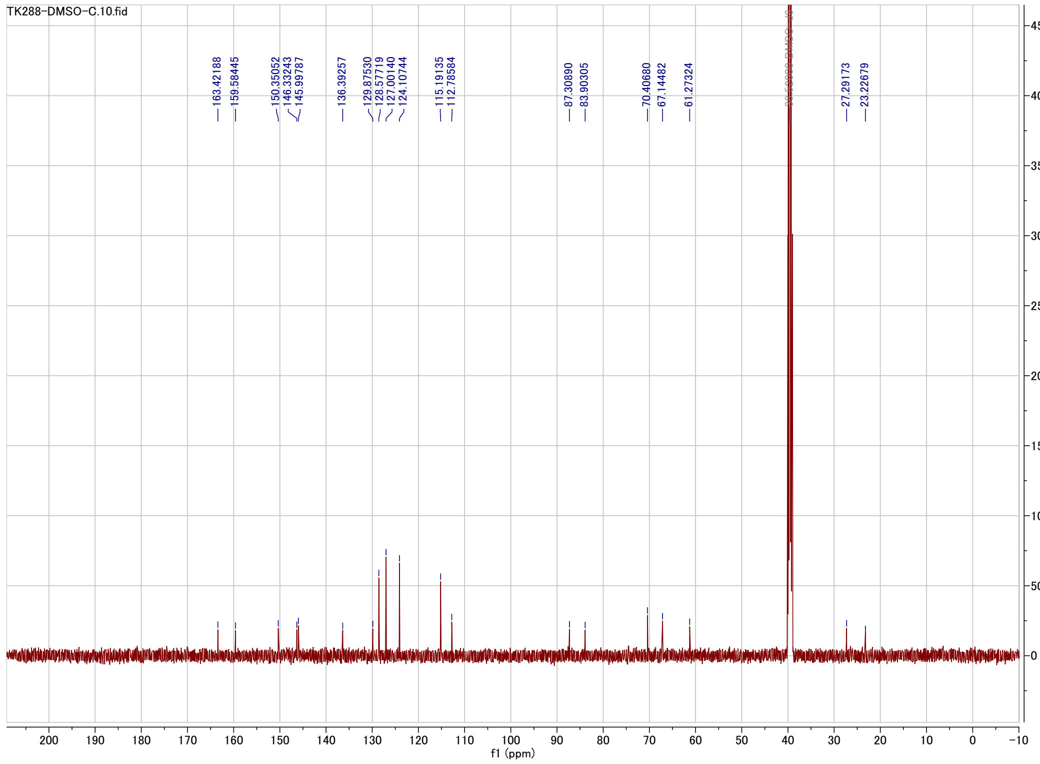

Fig. S12 ^{13}C-NMR spectrum of compound **5**.

TDCMAS ESI-TOF

Analysis Info

Analysis Name	D:\Data\yuasa_lab\kanamori\200115\TK288.2-000004.d	Acquisition Date	2020/01/14 11:39:26
Method	esi_posi_low.m	Operator	BDAL@DE
Sample Name	TK288.2-	Instrument / Ser#	micrOTOF 213750.10321
Comment			

Acquisition Parameter

Source Type	ESI	Ion Polarity	Positive	Set Nebulizer	0.3 Bar
Focus	Not active			Set Dry Heater	180 ‹C
Scan Begin	50 m/z	Set Capillary	4500 V	Set Dry Gas	4.0 l/min
Scan End	1200 m/z	Set End Plate Offset	-500 V	Set Divert Valve	Waste

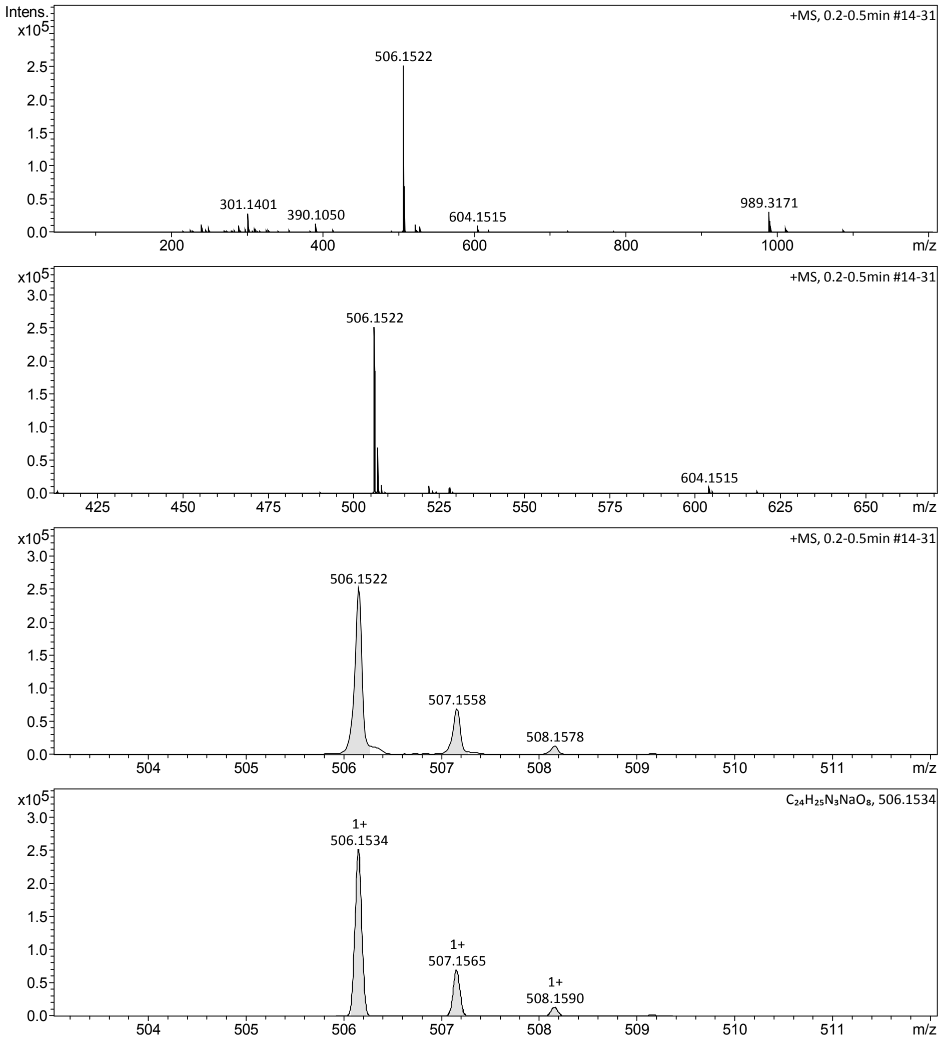

Fig. S13 ESI-TOF-MS spectra of compound **5**.

5-(3-[(4'-Nitro[1,1'-biphenyl]-4-yl)oxy]propane-1-yl)-5’-*O*-(4,4’-dimethoxytrityl)-2’-deoxyuridine (6)

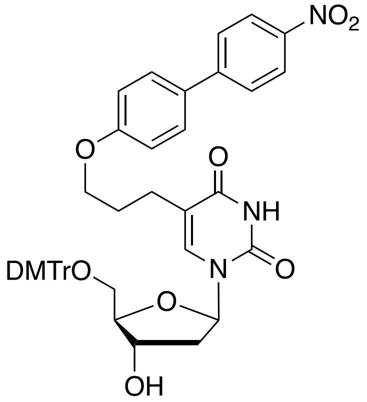

Compound **5** (117 mg, 0.242 mmol) was co-evaporated with dry pyridine. Then, to the solution of compound **5** in dry pyridine (1.2 mL) was added DMTrCl (98 mg, 0.289 mmol) under nitrogen atmosphere. The reaction mixture was stirred at room temperature for 5.5 h. Then, to the reaction mixture was added MeOH (1 mL). The solution was evaporated and diluted with CH_2Cl_2 and washed with water. The organic layer was evaporated and the residue was purified by column chromatography (60N gel, hexane-EtOAc, 1:1, v:v) to give compound **6** (170 mg, 89%) as yellow amorphous solid. R_f 0.32 (CH_2Cl_2/MeOH (19:1 v/v)); ^{1}H NMR (400 MHz, $CDCl_3$) δ 8.37 (s, 1H, N-H), 8.26 (d, J = 9.1 Hz, 2H, biphenyl), 7.67 (d, J = 9.1 Hz, 2H, biphenyl), 7.55 (s, 1H, H-6), 7.50 (d, J = 8.8 Hz, 2H, biphenyl), 7.38–7.43 (m, 2H, DMTr-), 7.20–7.33 (m, 7H, DMTr-), 6.80–6.87 (m, 6H, DMTr (4H), biphenyl (2H)), 6.39 (dd, J = 5.8 Hz, J = 7.8 Hz, 1H, H-1’), 4.48–4.56 (m, 1H, H-3’), 4.03 (dd, J = 3.3 Hz, J = 6.4 Hz, 1H, H-4’), 3.72–3.81 (m, 8H, -$CH_2CH_2C\underline{H_2}$-O-, -PhOMe ×2), 3.48 (dd, J = 3.5 Hz, J = 10.5 Hz, 1H, H-5’), 3.37 (dd, J = 3.1 Hz, 10.5 Hz, 1H, H-5’), 2.38 (ddd, J = 3.1 Hz, 6.0 Hz, 13.6 Hz, 1H, H-2’), 2.20–2.30 (m, 1H, H-2’), 2.12–2.20 (m, 1H, -$C\underline{H}HCH_2CH_2$-O-), 1.96–2.07 (m, 2H, OH, -$CH\underline{H}CH_2CH_2$-O-), 1.68–1.87 (m, 2H, -$CH_2C\underline{H_2}CH_2$-O-); ^{13}C NMR (126 MHz, $CDCl_3$) δ 163.4, 159.9, 158.9, 150.4, 147.3, 146.6, 144.4, 136.1, 135.5, 131.0, 130.2, 128.6, 128.3, 128.2, 127.3, 127.1, 124.3, 115.2, 114.8, 113.4, 87.0, 86.2, 84.8, 72.6, 67.5, 63.6, 55.4, 41.0, 28.4, 24.0; ESI-TOF-MS calcd for $C_{45}H_{43}N_3NaO_{10}^+$ $[M+Na]^+$ 808.2841; found 808.2861.

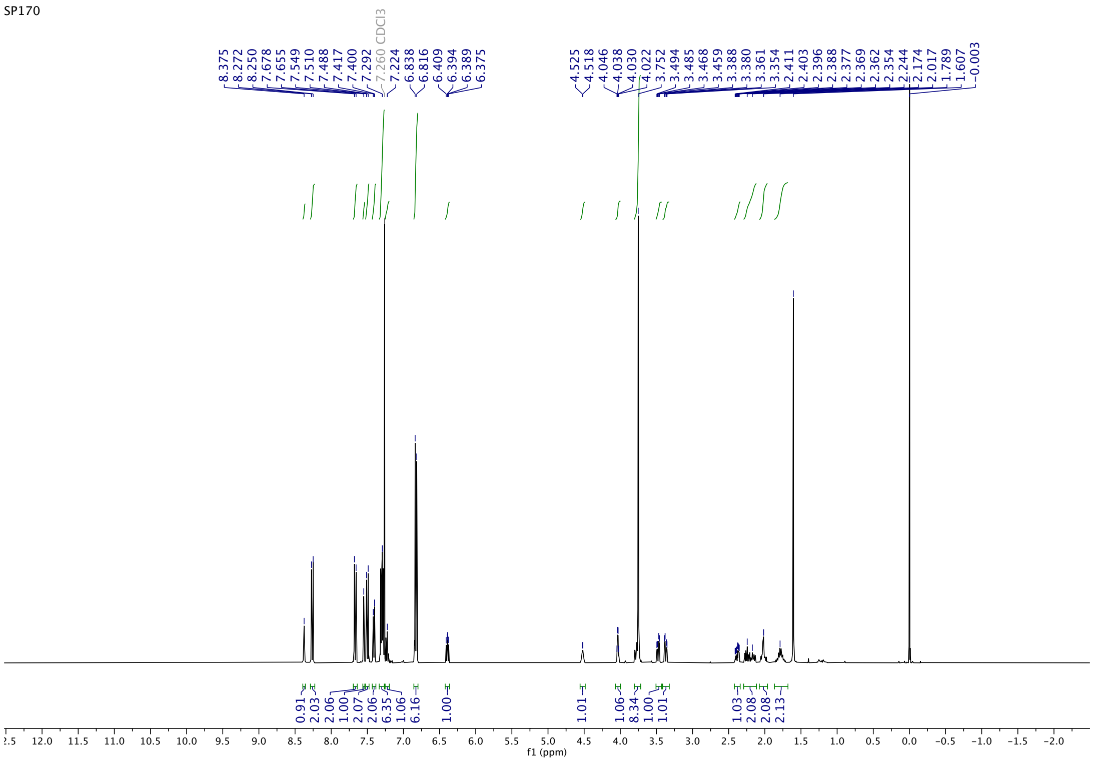

Fig. S14 ^{1}H-NMR spectrum of compound **6**.

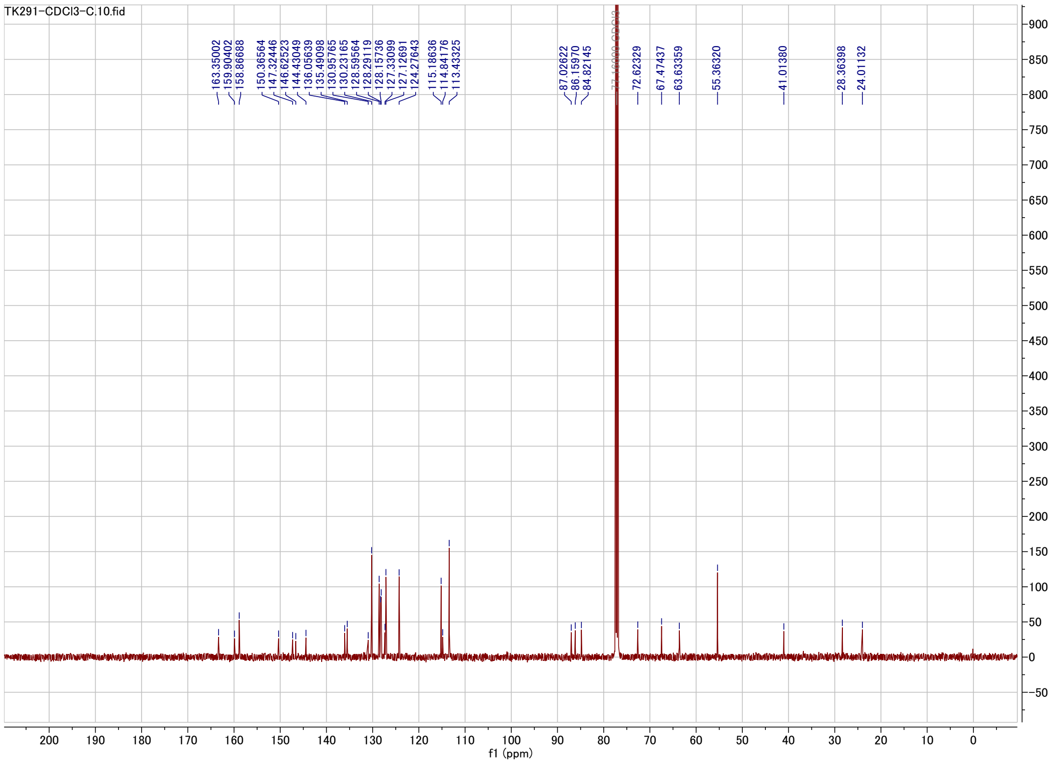

Fig. S15 ^{13}C-NMR spectrum of compound **6**.

TDCMAS ESI-TOF

Analysis Info

Analysis Name	D:\Data\yuasa_lab\kanamori\211214\SP291-000001.d	Acquisition Date	2021/12/13 13:33:49
Method	esi_posi_low.m	Operator	BDAL@DE
Sample Name	SP291-	Instrument / Ser#	micrOTOF 213750.10321
Comment			

Acquisition Parameter

Source Type	ESI	Ion Polarity	Positive	Set Nebulizer	0.3 Bar
Focus	Not active			Set Dry Heater	180 ‹C
Scan Begin	50 m/z	Set Capillary	4500 V	Set Dry Gas	4.0 l/min
Scan End	1800 m/z	Set End Plate Offset	-500 V	Set Divert Valve	Waste

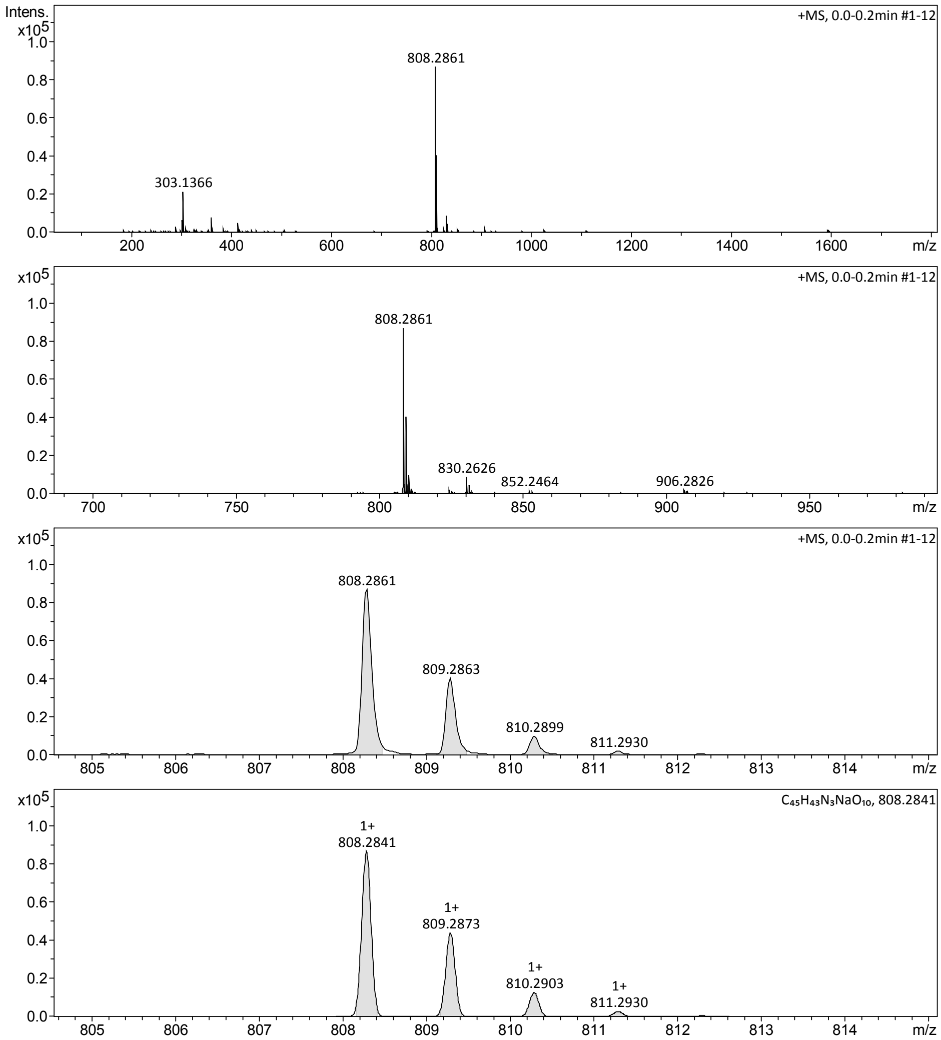

Fig. S16 ESI-TOF-MS spectra of compound **6**.

5-(3-[(4'-Nitro[1,1'-biphenyl]-4-yl)oxy]propane-1-yl)-5'-*O*-(4,4'-dimethoxytrityl)-2'-deoxyuridine-3'-*O*-[(2-cyanoethyl)-(*N*,*N*-diisopropyl)]phosphoramidite (7)

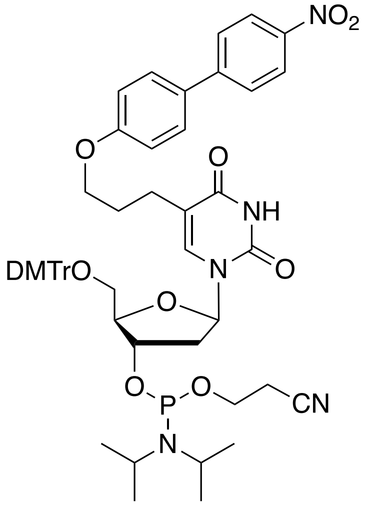

Compound **6** (96 mg, 0.122 mmol) was co-evaporated with dry pyridine and dry CH_2Cl_2. Then, to the solution of compound **6** in dry CH_2Cl_2 (1.2 mL) was added DIEA (68 μL, 0.390 mmol), 2-cyanoethyl-*N*,*N*-diisopropylchlorophosphoramidite (41 μL, 0.184 mmol). The reaction mixture was stirred at room temperature for 1 h. Then, it was diluted with ethyl acetate and washed with sat. $NaHCO_3$ aq. The organic layer was dried over Na_2SO_4 and evaporated. The residue was purified by column chromatography (C200 silica gel, hexane-EtOAc, 3:7, v/v, with 0.5% pyridine) to give crude diastereomeric mixture of compound **7** (93 mg) as yellow amorphous slid. R_f 0.69, 0.74 (hexane/EtOAc (1:4 v/v)). The crude mixture was directly used for DNA oligomer synthesis without further purification. ESI-TOF-MS calcd for $C_{54}H_{60}N_5NaO_{11}P^+$ $[M+Na]^+$ 1008.3919; found 1008.3933.

TDCMAS ESI-TOF

Analysis Info

Analysis Name	D:\Data\yuasa_lab\kanamori\211214\SP171-000001.d	Acquisition Date	2021/12/13 13:47:48
Method	esi_posi_low.m	Operator	BDAL@DE
Sample Name	SP171-	Instrument / Ser#	micrOTOF 213750.10321
Comment			

Acquisition Parameter

Source Type	ESI	Ion Polarity	Positive	Set Nebulizer	0.3 Bar
Focus	Not active			Set Dry Heater	180 ‹C
Scan Begin	50 m/z	Set Capillary	4500 V	Set Dry Gas	4.0 l/min
Scan End	2200 m/z	Set End Plate Offset	-500 V	Set Divert Valve	Waste

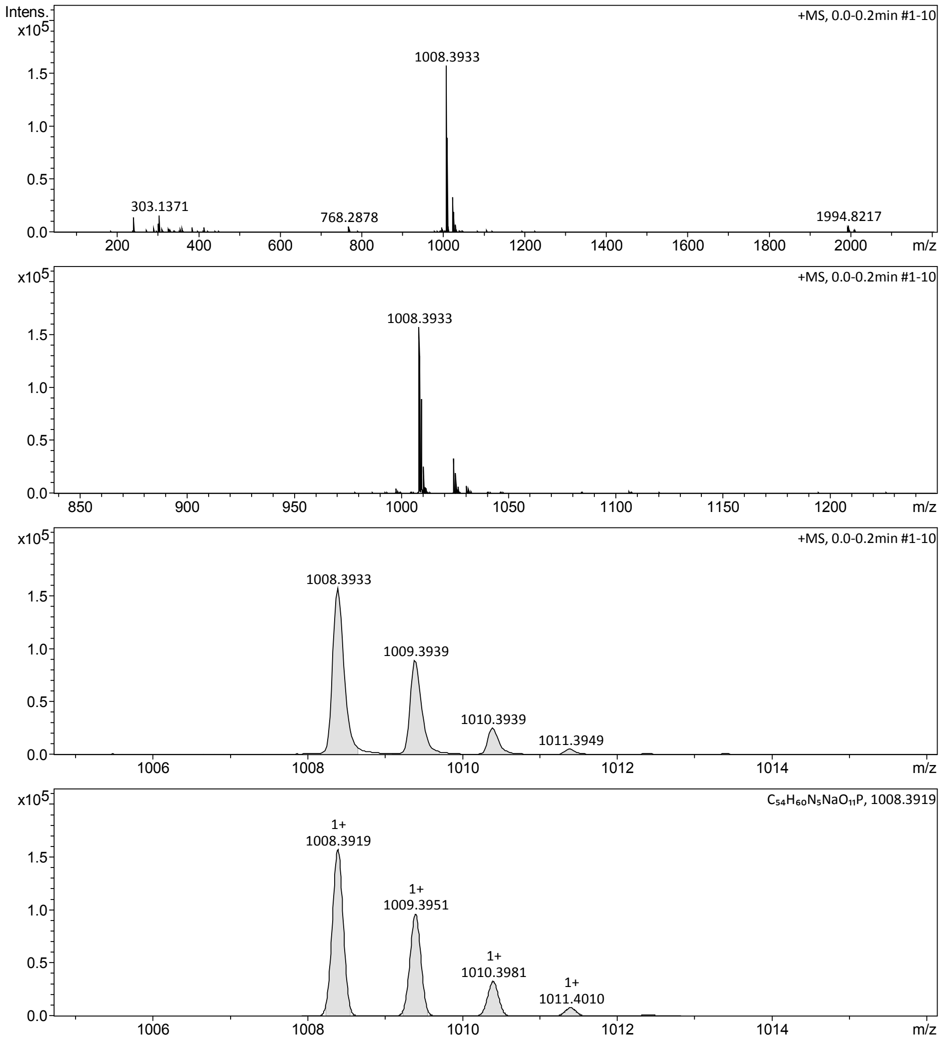

Fig. S17 ESI-TOF-MS spectra of compound **7**.

Oligonucleotide synthesis and purification

Modified oligonucleotides (ONs) used in this study were synthesized on a DNA/RNA synthesizer ns-8 II (GeneDesign) with the standard protocol for phosphoramidite chemistry. For the incorporation of photosensitizer-appended thymidine unit (P), phosphoramidite unit of P (compound **7**) was coupled by manual synthesis. After the synthesis, ONs were cleaved from the CPG support and deprotected with 55% aq. ammonia at room temperature for 8~12 h. Then were performed DMTr-on mode Sep-Pak purification and reverse phase (RP) HPLC purification on a preparative HPLC system (Shimadzu) equipped with Waters X-bridge column (prep C18 5 μm, 10 x 250 mm) and eluted with 30 mM ammonia acetate buffer/MeCN solvent system. Obtained ONs were desalted with Sep-Pak cartridge. Then, the purified ONs were analyzed on an RP-HPLC system (Waters Alliance 2695) equipped with Waters X-bridge column (C18 5 μm, 4.6 x 150 mm) and eluted with 30mM ammonia acetate buffer/MeCN solvent system.

ON0, **ON0'**, **ON1'[n] (n = -17 ~ -1)**, **ON2'[n] (n = +1 ~ +19)**, **ON3'[n] (n = +21, +23)**, **ON4'[+5]**, **ON4''[+5]**, **ON5'[+10]**, **ON6[+10]**, and **ON6'[+10]** were purchased from Integrated DNA Technologies. For the concentration determination, the following molar absorption coefficients at 260 nm (ε_{260}) were calcutlated[6] with the assumption that ε_{260} of **BP-T** unit was equal to that of dT and that of deoxyinosine (dI) was equal to that of dG.

Table S1 Molar absorption coefficients at 260 nm (ε_{260}) for ONs used in this study.

ONs	ε_{260}
**ON0, ON1[n] (n = -17 ~ -1), ON2[n] (n = +1 ~ +19), ON2[+5], ON2[+10], ON6**	202200
**ON3[n] (n = +21, +23)**	242700
**ON0', ON1'[n] (n = -17 ~ -1), ON2'[n](n = +1 ~ +19), ON4'[+5], ON5'[+10], ON6'**	302700
**ON3'[n] (n = +21, +23)**	362700
**ON4''[+5]**	183400

Name	ON	Sequence	Position
Standard	ON0	5’-TTTTTCTTTTTTTTTTTTTTTTTTT-3’	
	ON0’	3’-AAAAAGAAAAAAAAAAAAAAAAAAA-5’	
dsDNA1[-17]	ON1[-17]	5’-TTTCTTTTTTTTTTTTTTTTPTTTT-3’	
	ON1’[-17]	3’-AAAGAAAAAAAAAAAAAAAAAAAAA-5’	-17
dsDNA1[-13]	ON1[-13]	5’-TTTTTTTCTTTTTTTTTTTTPTTTT-3’	
	ON1’[-13]	3’-AAAAAAAGAAAAAAAAAAAAAAAAA-5’	-13
dsDNA1[-9]	ON1[-9]	5’-TTTTTTTTTTTCTTTTTTTTPTTTT-3’	
	ON1’ [-9]	3’-AAAAAAAAAAAGAAAAAAAAAAAAA-5’	-9
dsDNA1[-5]	ON1[-5]	5’-TTTTTTTTTTTTTTTCTTTTPTTTT-3’	
	ON1’ [-5]	3’-AAAAAAAAAAAAAAAGAAAAAAAAA-5’	-5
dsDNA1[-1]	ON1[-1]	5’-TTTTTTTTTTTTTTTTTTTCPTTTT-3’	
	ON1’ [-1]	3’-AAAAAAAAAAAAAAAAAAAGAAAAA-5’	-1
dsDNA2[+1]	ON2[+1]	5’-TTTTPCTTTTTTTTTTTTTTTTTTT-3’	
	ON2’ [+1]	3’-AAAAAGAAAAAAAAAAAAAAAAAAA-5’	1
dsDNA2[+2]	ON2[+2]	5’-TTTTPTCTTTTTTTTTTTTTTTTTT-3’	
	ON2’ [+2]	3’-AAAAAAGAAAAAAAAAAAAAAAAAA-5’	2
dsDNA2[+3]	ON2[+3]	5’-TTTTPTTCTTTTTTTTTTTTTTTTT-3’	
	ON2’ [+3]	3’-AAAAAAAGAAAAAAAAAAAAAAAAA-5’	3
dsDNA2[+4]	ON2[+4]	5’-TTTTPTTTCTTTTTTTTTTTTTTTT-3’	
	ON2’ [+4]	3’-AAAAAAAAGAAAAAAAAAAAAAAAA-5’	4
dsDNA2[+5]	ON2[+5]	5’-TTTTPTTTTCTTTTTTTTTTTTTTT-3’	
	ON2’ [+5]	3’-AAAAAAAAAGAAAAAAAAAAAAAAA-5’	5
dsDNA2[+6]	ON2[+6]	5’-TTTTPTTTTTCTTTTTTTTTTTTTT-3’	
	ON2’ [+6]	3’-AAAAAAAAAAGAAAAAAAAAAAAAA-5’	6
dsDNA2[+7]	ON2[+7]	5’-TTTTPTTTTTTCTTTTTTTTTTTTT-3’	
	ON2’ [+7]	3’-AAAAAAAAAAAGAAAAAAAAAAAAA-5’	7
dsDNA2[+8]	ON2[+8]	5’-TTTTPTTTTTTTCTTTTTTTTTTTT-3’	
	ON2’ [+8]	3’-AAAAAAAAAAAAGAAAAAAAAAAAA-5’	8
dsDNA2[+9]	ON2[+9]	5’-TTTTPTTTTTTTTCTTTTTTTTTTT-3’	
	ON2’ [+9]	3’-AAAAAAAAAAAAAGAAAAAAAAAAA-5’	9
dsDNA2[+10]	ON2[+10]	5’-TTTTPTTTTTTTTTCTTTTTTTTTT-3’	
	ON2’ [+10]	3’-AAAAAAAAAAAAAAGAAAAAAAAAA-5’	10
dsDNA2[+11]	ON2[+11]	5’-TTTTPTTTTTTTTTTCTTTTTTTTT-3’	
	ON2’ [+11]	3’-AAAAAAAAAAAAAAAGAAAAAAAAA-5’	11
dsDNA2[+13]	ON2[+13]	5’-TTTTPTTTTTTTTTTTTCTTTTTTT-3’	
	ON2’ [+13]	3’-AAAAAAAAAAAAAAAAAGAAAAAAA-5’	13
dsDNA2[+15]	ON2[+15]	5’-TTTTPTTTTTTTTTTTTTTCTTTTT-3’	
	ON2’ [+15]	3’-AAAAAAAAAAAAAAAAAAAGAAAAA-5’	15
dsDNA2[+17]	ON2[+17]	5’-TTTTPTTTTTTTTTTTTTTTTCTTT-3’	
	ON2’ [+17]	3’-AAAAAAAAAAAAAAAAAAAAAGAAA-5’	17
dsDNA2[+19]	ON2[+19]	5’-TTTTPTTTTTTTTTTTTTTTTTTCT-3’	
	ON2’ [+19]	3’-AAAAAAAAAAAAAAAAAAAAAAAGA-5’	19
dsDNA3[+21]	ON3[+21]	5’-TTTTPTTTTTTTTTTTTTTTTTTTTCTTTT-3’	
	ON3’ [+21]	3’-AAAAAAAAAAAAAAAAAAAAAAAAAGAAAA-5’	21
dsDNA3[+23]	ON3[+23]	5’-TTTTPTTTTTTTTTTTTTTTTTTTTTTCTT-3’	
	ON3’ [+23]	3’-AAAAAAAAAAAAAAAAAAAAAAAAAAAGAA-5’	23
dsDNA4[+5]	ON2[+5]	5’-TTTTPTTTTCTTTTTTTTTTTTTTT-3’	
	ON4’ [+5]	3’-AAAAAAAAAGAAAAAAAAAAAAAAA-FAM-5'	5
	ON4” [+5]	3’-pAAAAAAAAAAAAAAA-FAM-5' (p = phosphate)	
dsDNA5[+10]	ON2[+10]	5’-TTTTPTTTTTTTTTCTTTTTTTTTT-3’	
	ON5’ [+10]	3’-AAAAAAAAAAAAAAIAAAAAAAAAA-5’	10
dsDNA6	ON6	5’-TTTTTTTTTTTTTTCTTTTTTTTTT-3’	
	ON2’ [+10]	3’-AAAAAAAAAAAAAAGAAAAAAAAAA-5’	

Fig. S18 Sequence list of ONs used in this study. Note that **ON1’[-13]**, **ON1’[-9]**, **ON1’[-5]**, and **ON1’[-1]** were identical to **ON2[+3]**, **ON2[+7]**, **ON2[+11]**, and **ON2[+15]**, respectively.

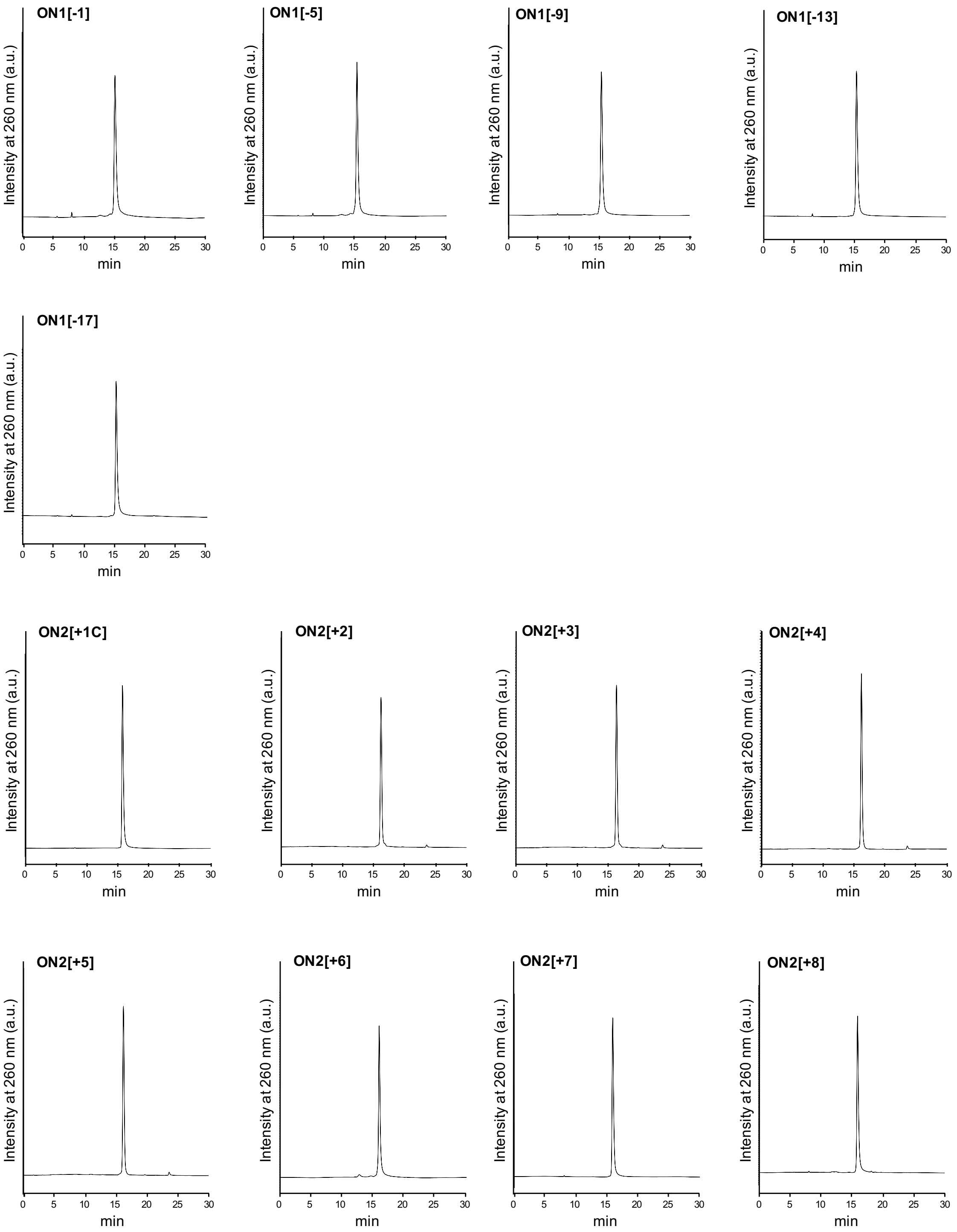

Fig. S19 Reversed-phase HPLC chromatographs of **ON1[n] (n = -1 ~ -17)** and **ON2[n] (n = +1 ~ +8)** after RP-HPLC purification. Conditions: 30 °C with a linear gradient of 0-30% acetonitrile in 30 mM NH_4OAc buffer at a flow rate of 1.0 mL/min for 30 min.

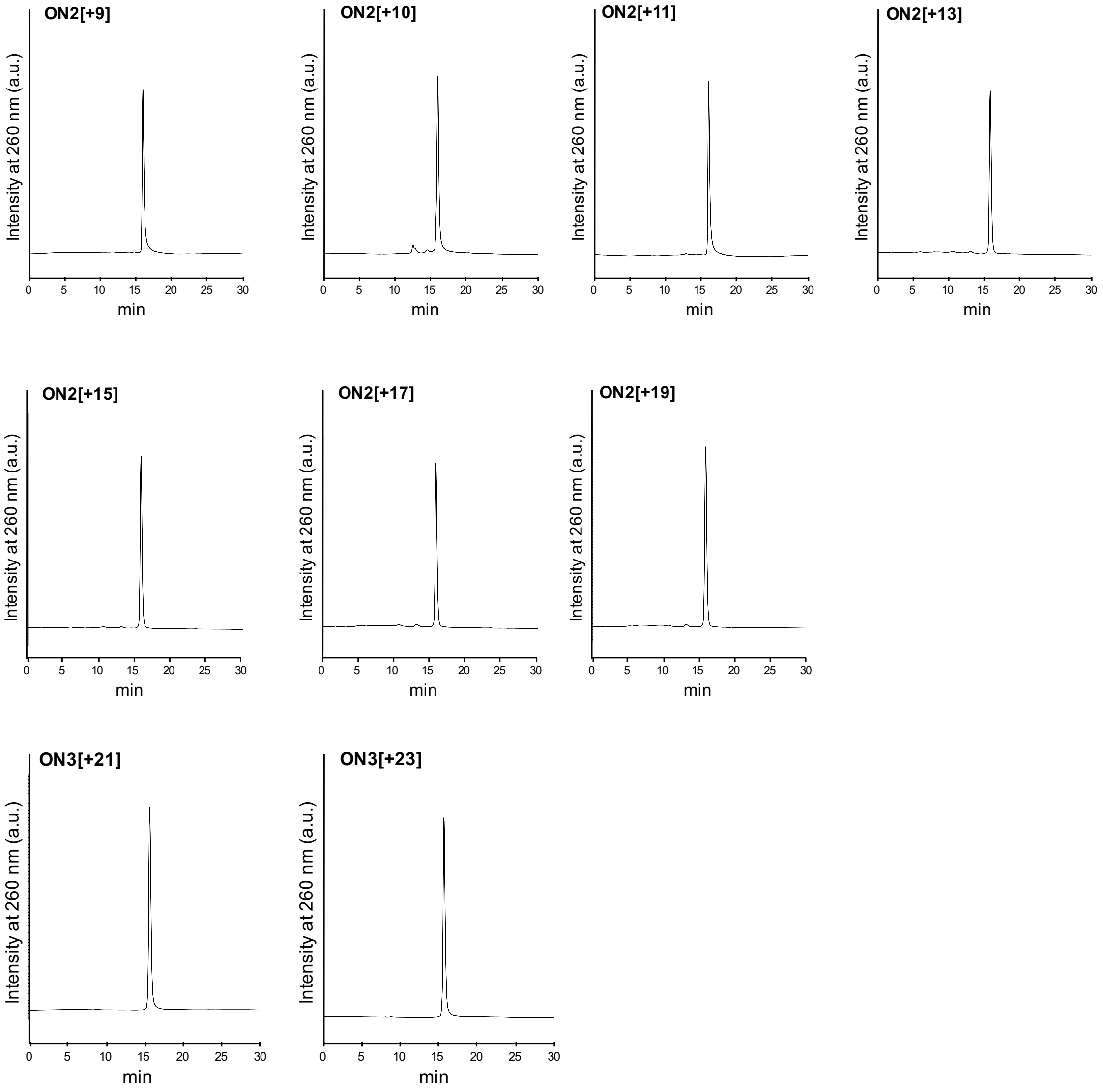

Fig. S20 Reversed-phase HPLC chromatographs of **ON2[n] (n = +9 ~ +19)** and **ON3[n] (n = +21, +23)** after RP-HPLC purification. Conditions: 30 °C with a linear gradient of 0-30% acetonitrile in 30 mM NH_4OAc buffer at a flow rate of 1.0 mL/min for 30 min.

Table S2 ESI-TOF-MS data of ONs synthesized in this study.

ONs	calcd MW for [M-H]$^-$	fould MW for [M-H]$^-$ (deconvoluted)
**ON1[-17]**	7768.1	7767.6
**ON1[-13]**	7768.1	7767.6
**ON1[-9]**	7768.1	7767.6
**ON1[-5]**	7768.1	7767.6
**ON1[-1]**	7768.1	7767.7
**ON2[+1]**	7768.1	7767.8
**ON2[+2]**	7768.1	7767.6
**ON2[+3]**	7768.1	7767.5
**ON2[+4]**	7768.1	7767.6
**ON2[+5]**	7768.1	7767.6
**ON2[+6]**	7768.1	7767.6
**ON2[+7]**	7768.1	7767.5
**ON2[+8]**	7768.1	7767.4
**ON2[+9]**	7768.1	7767.6
**ON2[+10]**	7768.1	7767.5
**ON2[+11]**	7768.1	7767.6
**ON2[+13]**	7768.1	7767.7
**ON2[+15]**	7768.1	7768.0
**ON2[+17]**	7768.1	7768.1
**ON2[+19]**	7768.1	7768.0
**ON3[+21]**	9289.1	9288.7
**ON3[+23]**	9289.1	9288.8

Display Report

Analysis Info

Analysis Name	D:\Data\ofcbunseki\irai\2022\yuasa_lab\kanamori\220825\ODN-NBP+17C-000001.d	Acquisition Date	2022/08/24 11:35:58
Method	220825.m	Operator	BDAL@DE
Sample Name	ODN-NBP+17C-	Instrument	micrOTOF 213750.10321
Comment			

Acquisition Parameter

Source Type	ESI	Ion Polarity	Negative	Set Nebulizer	0.3 Bar
Focus	Not active			Set Dry Heater	180 ‹C
Scan Begin	400 m/z	Set Capillary	3000 V	Set Dry Gas	4.0 l/min
Scan End	3000 m/z	Set End Plate Offset	-500 V	Set Divert Valve	Waste

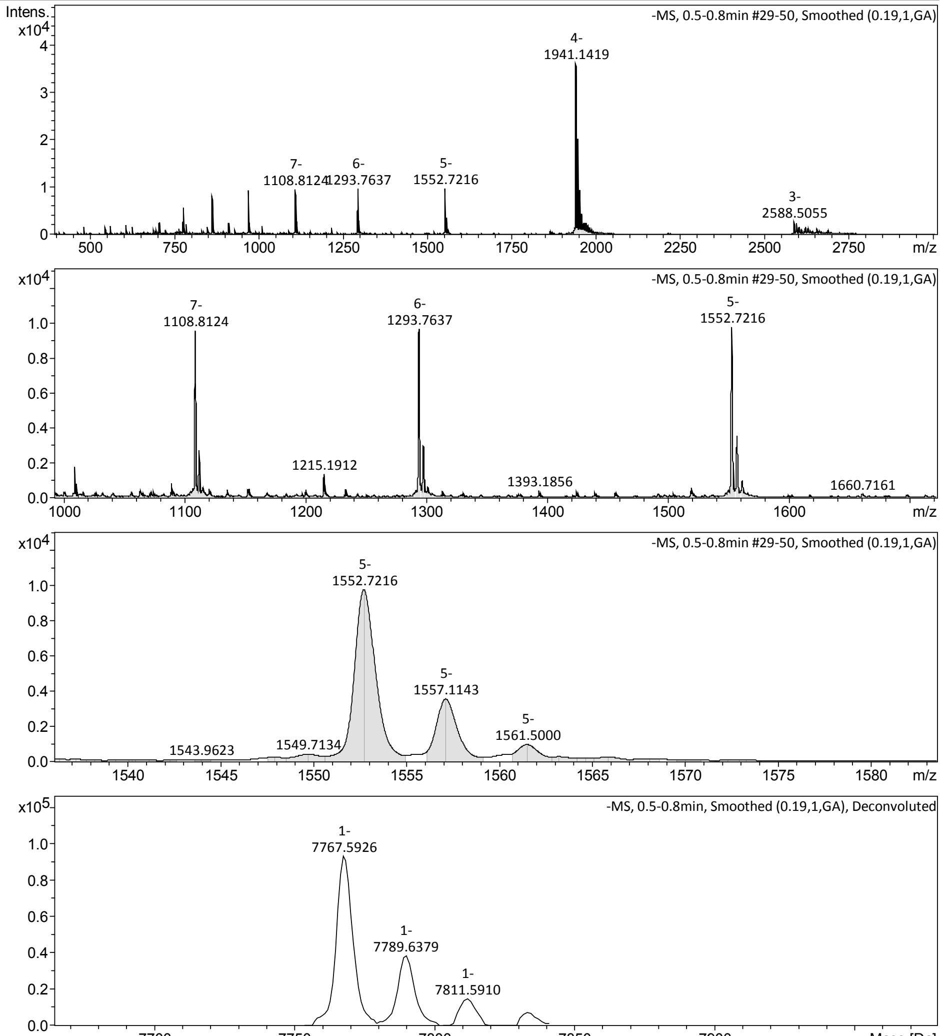

Fig. S21 ESI-TOF-MS spectra of **ON1[-17]**.

Display Report

Analysis Info

Analysis Name	D:\Data\ofcbunseki\irai\2022\yuasa_lab\kanamori\220825\ODN-NBP+13C-000001.d	Acquisition Date	2022/08/24 11:28:16
Method	220825.m	Operator	BDAL@DE
Sample Name	ODN-NBP+13C-	Instrument	micrOTOF 213750.10321
Comment			

Acquisition Parameter

Source Type	ESI	Ion Polarity	Negative	Set Nebulizer	0.3 Bar
Focus	Not active			Set Dry Heater	180 ‹C
Scan Begin	400 m/z	Set Capillary	3000 V	Set Dry Gas	4.0 l/min
Scan End	3000 m/z	Set End Plate Offset	-500 V	Set Divert Valve	Waste

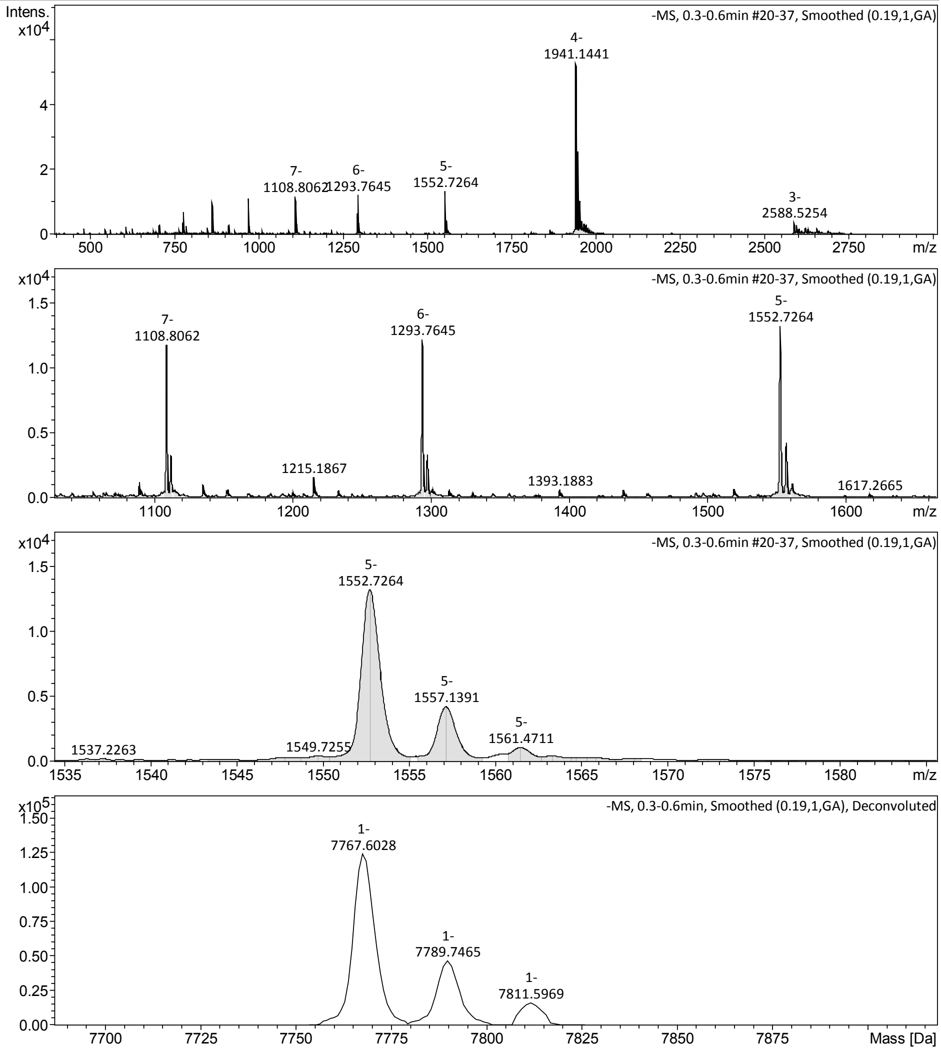

Fig. S22 ESI-TOF-MS spectra of **ON1[-13]**.

Display Report

Analysis Info

Analysis Name	D:\Data\ofcbunseki\irai\2022\yuasa_lab\kanamori\220825\ODN-NBP+9C-000001.d	Acquisition Date	2022/08/24 11:20:52
Method	220825.m	Operator	BDAL@DE
Sample Name	ODN-NBP+9C-	Instrument	micrOTOF 213750.10321
Comment			

Acquisition Parameter

Source Type	ESI	Ion Polarity	Negative	Set Nebulizer	0.3 Bar
Focus	Not active			Set Dry Heater	180 ‹C
Scan Begin	400 m/z	Set Capillary	3000 V	Set Dry Gas	4.0 l/min
Scan End	3000 m/z	Set End Plate Offset	-500 V	Set Divert Valve	Waste

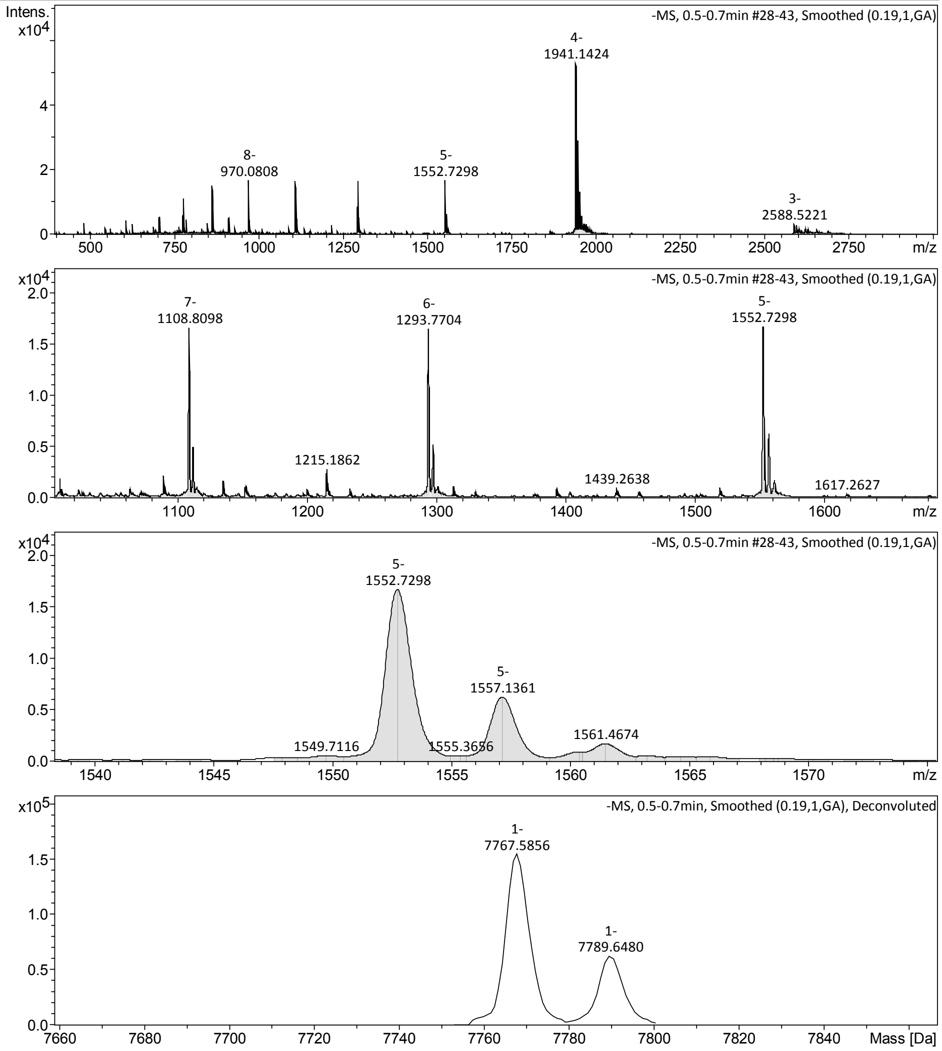

Fig. S23 ESI-TOF-MS spectra of **ON1[-9]**.

Display Report

Analysis Info

Acquisition Date 2022/08/24 11:12:05

Analysis Name D:\Data\ofcbunseki\irai\2022\yuasa_lab\kanamori\220825\ODN-NBP+5C-000001.d

Method 220825.m

Operator BDAL@DE

Sample Name ODN-NBP+5C-

Instrument micrOTOF 213750.10321

Comment

Acquisition Parameter

Source Type	ESI	Ion Polarity	Negative	Set Nebulizer	0.3 Bar
Focus	Not active			Set Dry Heater	180 ‹C
Scan Begin	400 m/z	Set Capillary	3000 V	Set Dry Gas	4.0 l/min
Scan End	3000 m/z	Set End Plate Offset	-500 V	Set Divert Valve	Waste

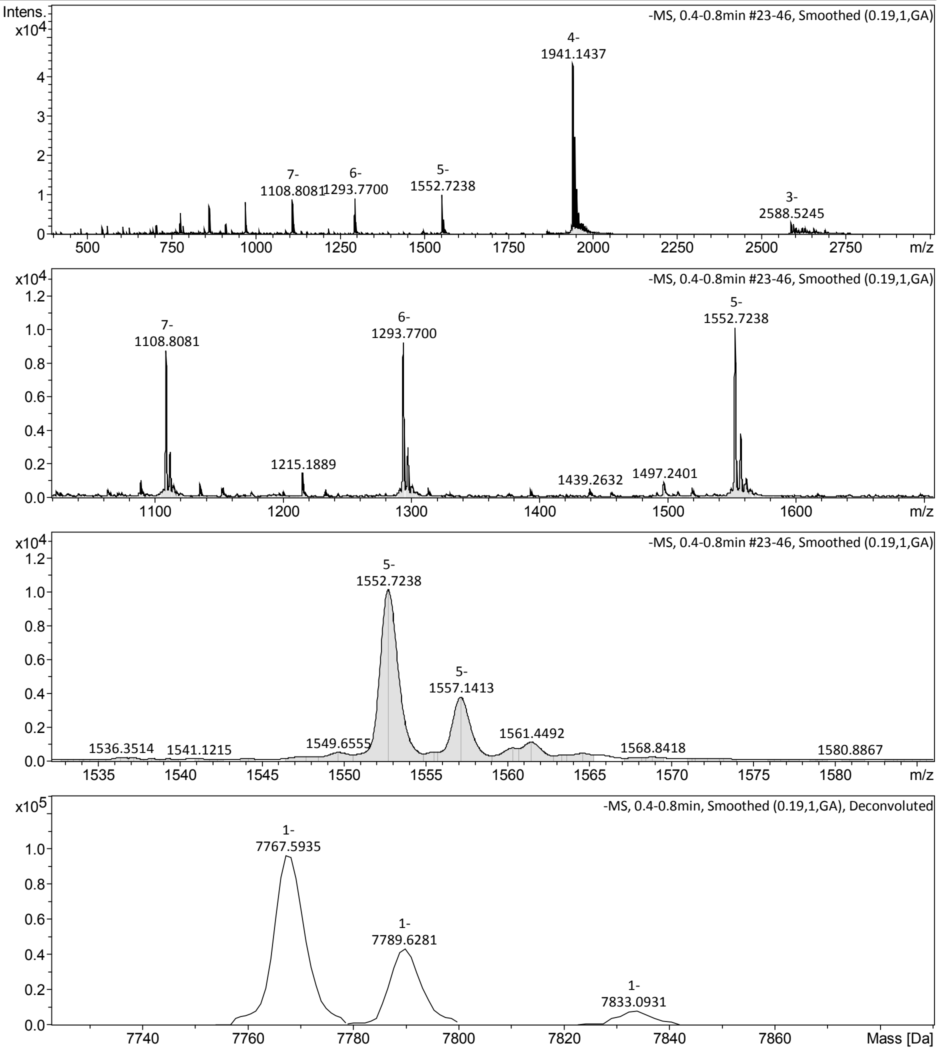

Fig. S24 ESI-TOF-MS spectra of **ON1[-5]**.

Display Report

Analysis Info

Analysis Name	D:\Data\ofcbunseki\irai\2022\yuasa_lab\kanamori\220825\ODN-NBP+1C-000001.d	Acquisition Date	2022/08/24 11:02:26
Method	220825.m	Operator	BDAL@DE
Sample Name	ODN-NBP+1C-	Instrument	micrOTOF 213750.10321
Comment			

Acquisition Parameter

Source Type	ESI	Ion Polarity	Negative	Set Nebulizer	0.3 Bar
Focus	Not active			Set Dry Heater	180 ‹C
Scan Begin	400 m/z	Set Capillary	3000 V	Set Dry Gas	4.0 l/min
Scan End	3000 m/z	Set End Plate Offset	-500 V	Set Divert Valve	Waste

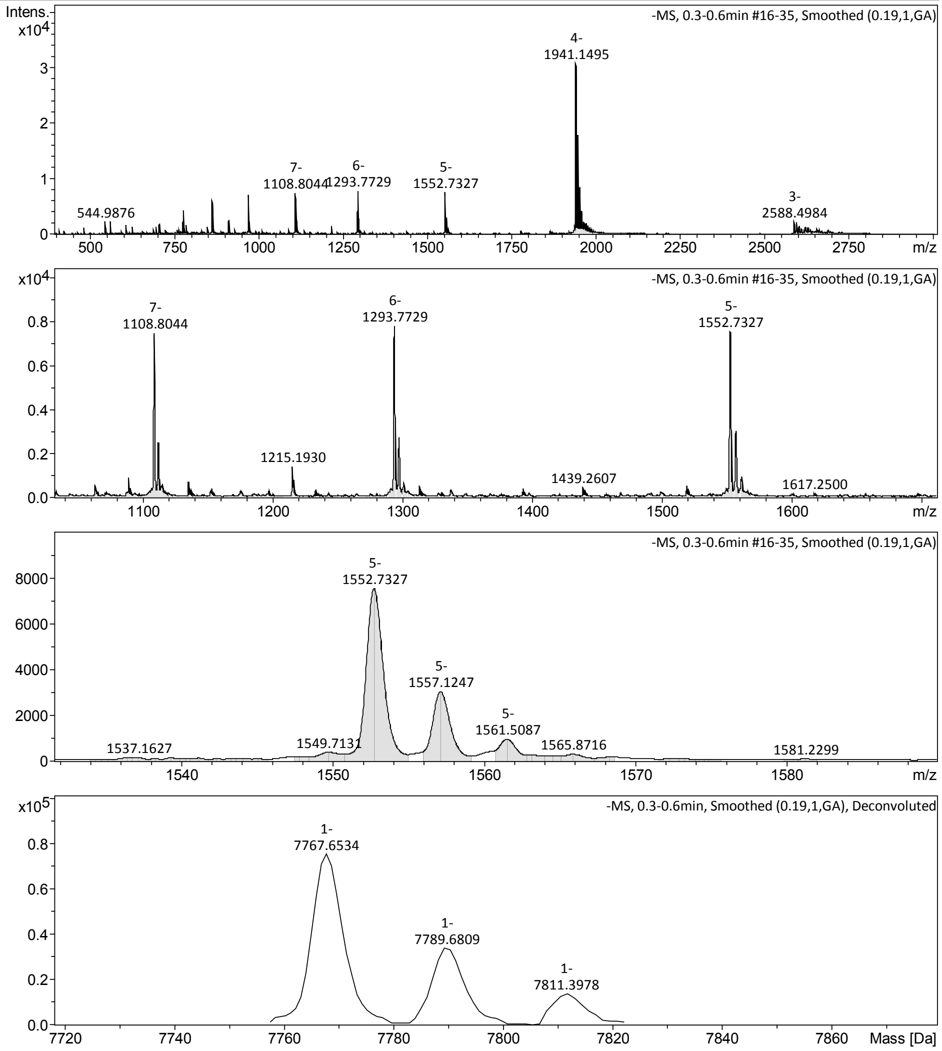

Bruker Compass DataAnalysis 4.2 printed: 2022/08/25 9:58:59 by: BDAL@DE 1 of 1

Fig. S25 ESI-TOF-MS spectra of **ON1[-1]**.

Display Report

Analysis Info

Analysis Name	D:\Data\yuasa_lab\kanamori\200115\ODN-5U-000001.d	Acquisition Date	2020/01/15 9:30:15
Method	esi_nega_wide.m	Operator	BDAL@DE
Sample Name	ODN-5U-	Instrument	micrOTOF 213750.10321
Comment			

Acquisition Parameter

Source Type	ESI	Ion Polarity	Negative	Set Nebulizer	0.3 Bar
Focus	Not active			Set Dry Heater	180 ‹C
Scan Begin	50 m/z	Set Capillary	3000 V	Set Dry Gas	4.0 l/min
Scan End	3000 m/z	Set End Plate Offset	-500 V	Set Divert Valve	Waste

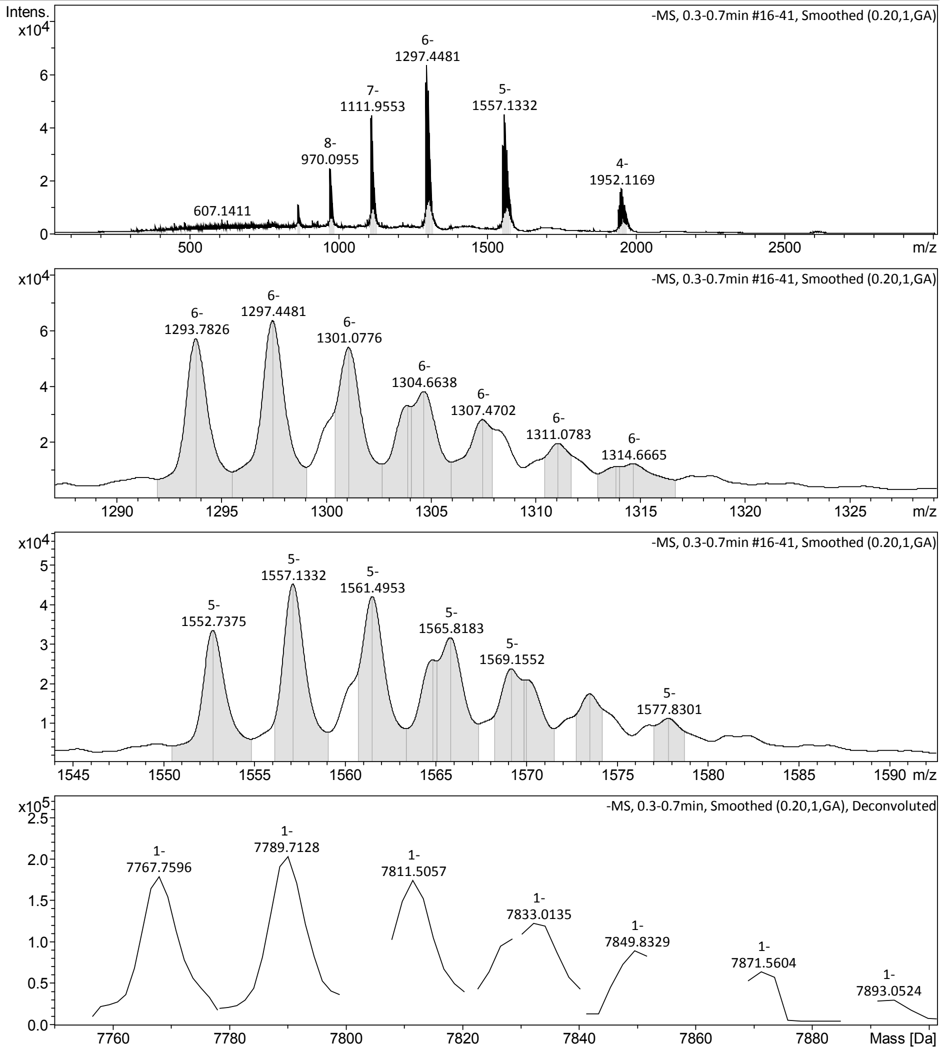

Fig. S26 ESI-TOF-MS spectra of **ON2[+1]**.

Display Report

Analysis Info

		Acquisition Date	2022/08/24 10:42:44
Analysis Name	D:\Data\ofcbunseki\irai\2022\yuasa_lab\kanamori\220825\ODN-NBP-2C-000002.d		
Method	220825.m	Operator	BDAL@DE
Sample Name	ODN-NBP-2C-	Instrument	micrOTOF 213750.10321
Comment			

Acquisition Parameter

Source Type	ESI	Ion Polarity	Negative	Set Nebulizer	0.3 Bar
Focus	Not active			Set Dry Heater	180 ‹C
Scan Begin	400 m/z	Set Capillary	3000 V	Set Dry Gas	4.0 l/min
Scan End	3000 m/z	Set End Plate Offset	-500 V	Set Divert Valve	Waste

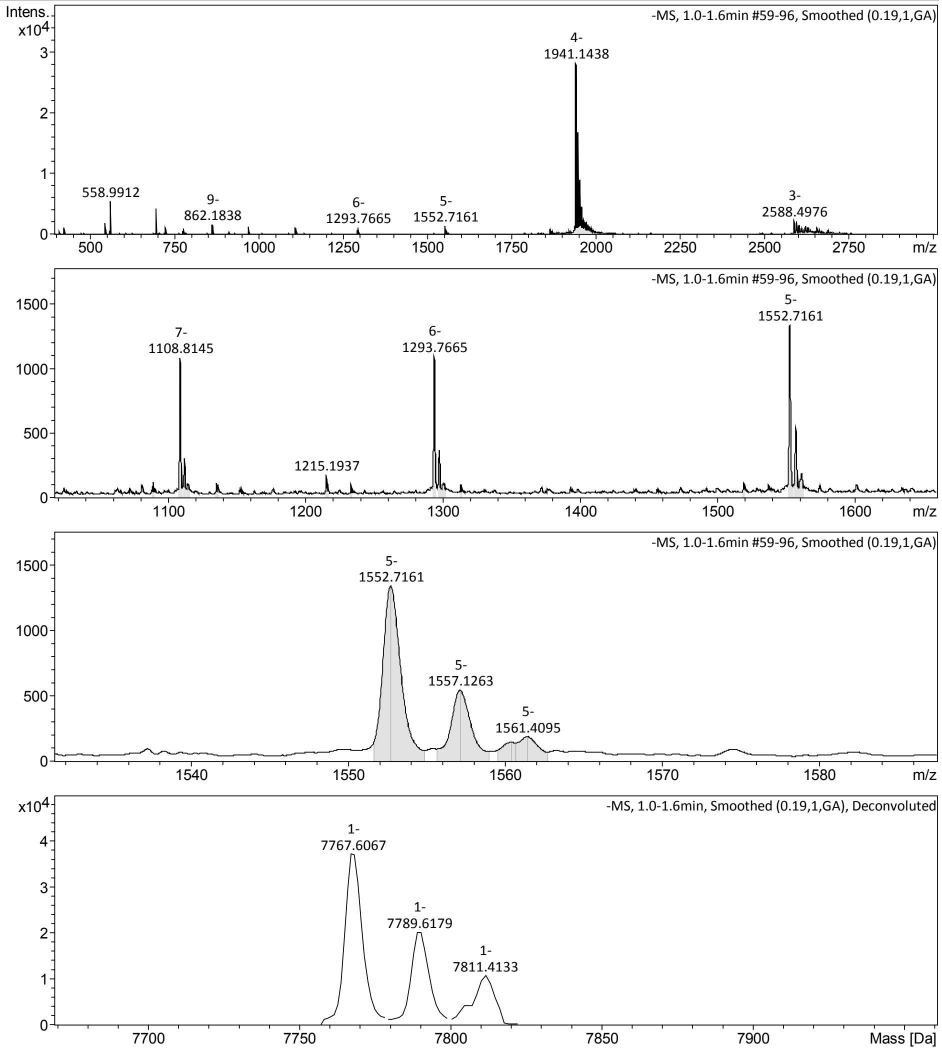

Fig. S27 ESI-TOF-MS spectra of **ON2[+2]**.

Display Report

Analysis Info

Analysis Name	D:\Data\yuasa_lab\kanamori\220114\ODN-NBC-3C-000003.d	Acquisition Date	2022/01/14 8:52:30
Method	esi_nega_wide.m	Operator	BDAL@DE
Sample Name	ODN-NBC-3C-	Instrument	micrOTOF 213750.10321
Comment			

Acquisition Parameter

Source Type	ESI	Ion Polarity	Negative	Set Nebulizer	0.3 Bar
Focus	Not active			Set Dry Heater	180 ‹C
Scan Begin	50 m/z	Set Capillary	1800 V	Set Dry Gas	4.0 l/min
Scan End	3000 m/z	Set End Plate Offset	-500 V	Set Divert Valve	Waste

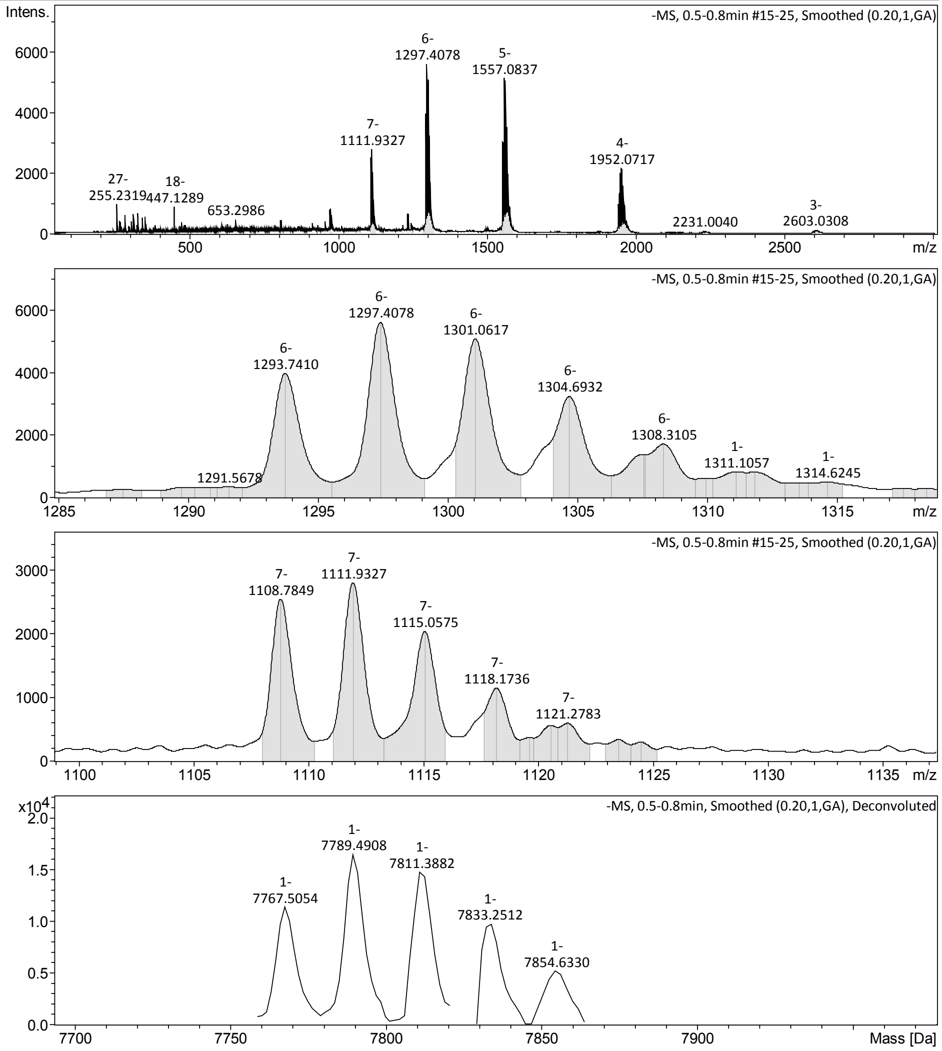

Fig. S28 ESI-TOF-MS spectra of **ON2[+3]**.

Display Report

Analysis Info

Analysis Name	D:\Data\yuasa_lab\kanamori\220118\ODN-NBC-4C-000001.d	Acquisition Date	2022/01/18 9:16:52
Method	esi_nega_wide.m	Operator	BDAL@DE
Sample Name	ODN-NBC-4C-	Instrument	micrOTOF 213750.10321
Comment			

Acquisition Parameter

Source Type	ESI	Ion Polarity	Negative	Set Nebulizer	0.3 Bar
Focus	Not active			Set Dry Heater	180 ‹C
Scan Begin	50 m/z	Set Capillary	1800 V	Set Dry Gas	4.0 l/min
Scan End	3000 m/z	Set End Plate Offset	-500 V	Set Divert Valve	Waste

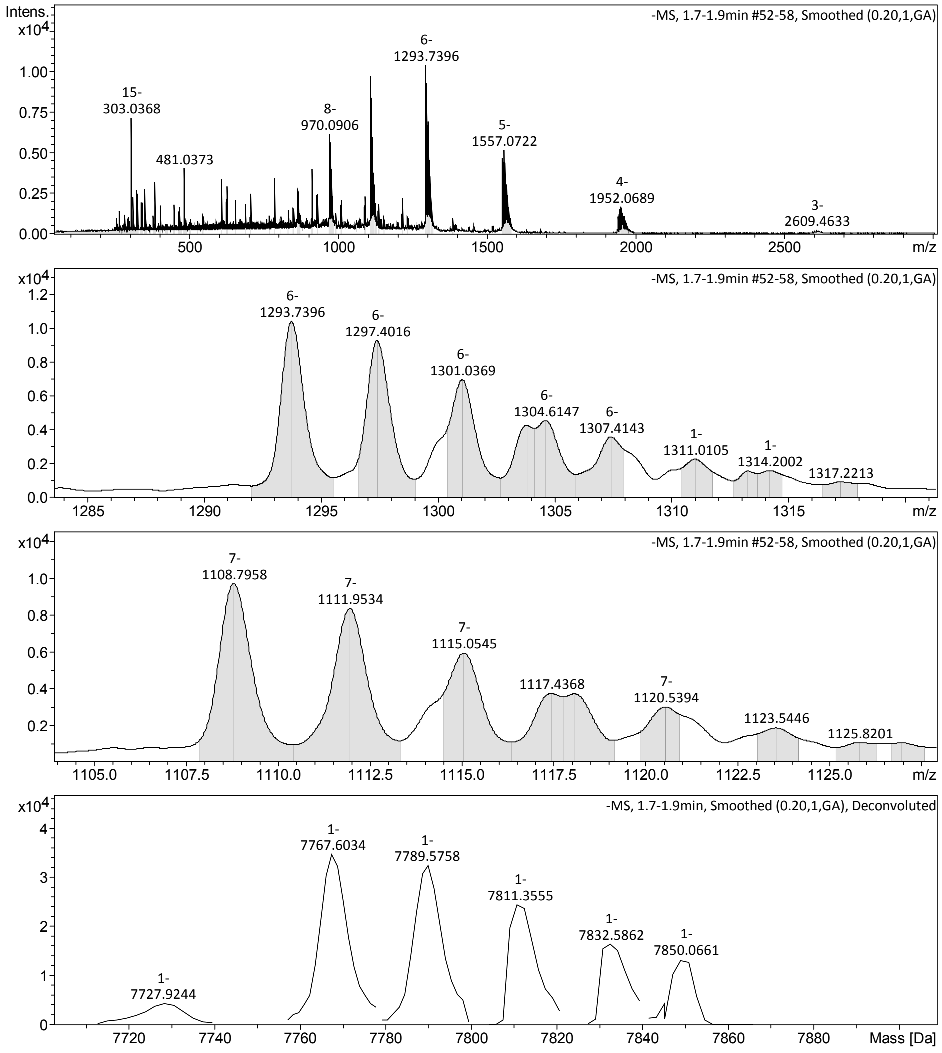

Fig. S29 ESI-TOF-MS spectra of **ON2[+4]**.

Display Report

Analysis Info

Acquisition Date 2022/01/14 9:25:12

Analysis Name	D:\Data\yuasa_lab\kanamori\220114\ODN-NBC-5C-000002.d		
Method	esi_nega_wide.m	Operator	BDAL@DE
Sample Name	ODN-NBC-5C-	Instrument	micrOTOF 213750.10321
Comment			

Acquisition Parameter

Source Type	ESI	Ion Polarity	Negative	Set Nebulizer	0.3 Bar
Focus	Not active			Set Dry Heater	180 ‹C
Scan Begin	50 m/z	Set Capillary	1800 V	Set Dry Gas	4.0 l/min
Scan End	3000 m/z	Set End Plate Offset	-500 V	Set Divert Valve	Waste

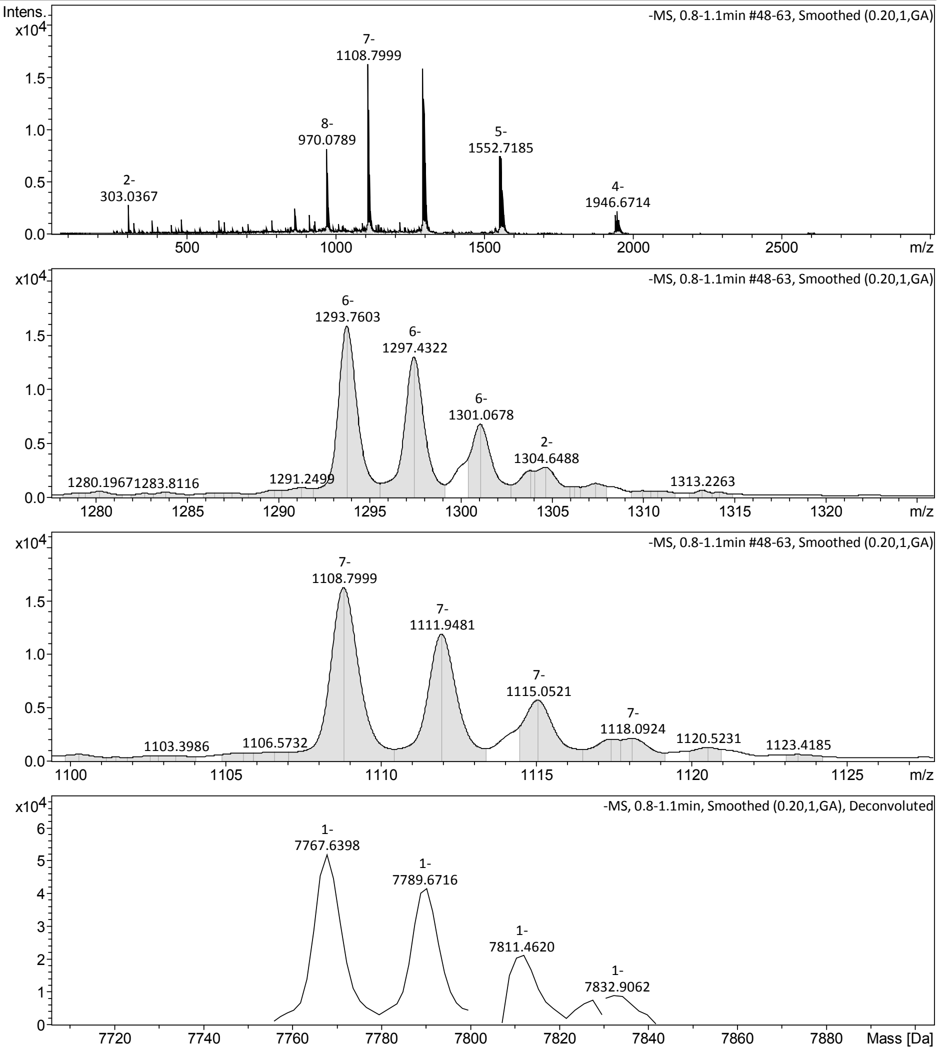

Fig. S30 ESI-TOF-MS spectra of **ON2[+5]**.

Display Report

Analysis Info

		Acquisition Date	2022/01/14 9:38:02
Analysis Name	D:\Data\yuasa_lab\kanamori\220114\ODN-NBC-6C-000002.d		
Method	esi_nega_wide.m	Operator	BDAL@DE
Sample Name	ODN-NBC-6C-	Instrument	micrOTOF 213750.10321
Comment			

Acquisition Parameter

Source Type	ESI	Ion Polarity	Negative	Set Nebulizer	0.3 Bar
Focus	Not active			Set Dry Heater	180 ‹C
Scan Begin	50 m/z	Set Capillary	1800 V	Set Dry Gas	4.0 l/min
Scan End	3000 m/z	Set End Plate Offset	-500 V	Set Divert Valve	Waste

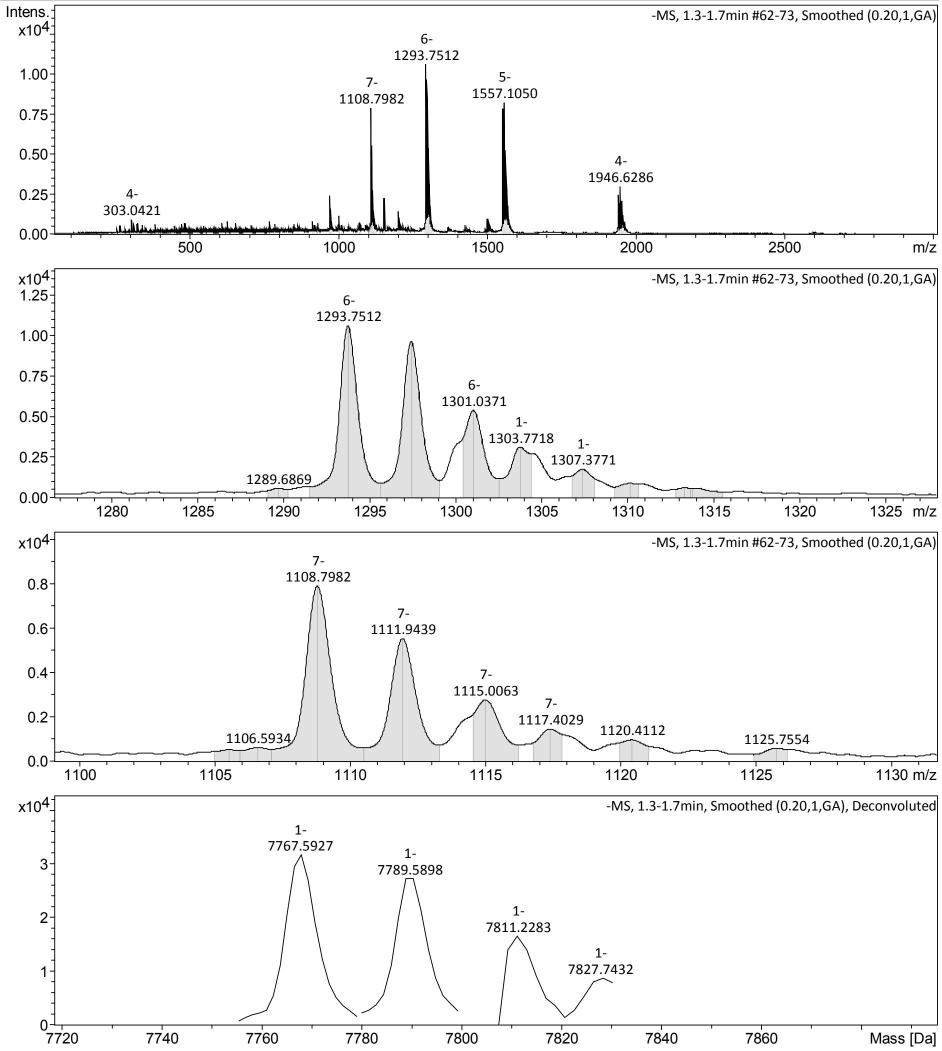

Fig. S31 ESI-TOF-MS spectra of **ON2[+6]**.

Display Report

Analysis Info Acquisition Date 2022/01/14 9:49:36

Analysis Name	D:\Data\yuasa_lab\kanamori\220114\ODN-NBC-7C-000001.d		
Method	esi_nega_wide.m	Operator	BDAL@DE
Sample Name	ODN-NBC-7C-	Instrument	micrOTOF 213750.10321
Comment			

Acquisition Parameter

Source Type	ESI	Ion Polarity	Negative	Set Nebulizer	0.3 Bar
Focus	Not active			Set Dry Heater	180 ‹C
Scan Begin	50 m/z	Set Capillary	1800 V	Set Dry Gas	4.0 l/min
Scan End	3000 m/z	Set End Plate Offset	-500 V	Set Divert Valve	Waste

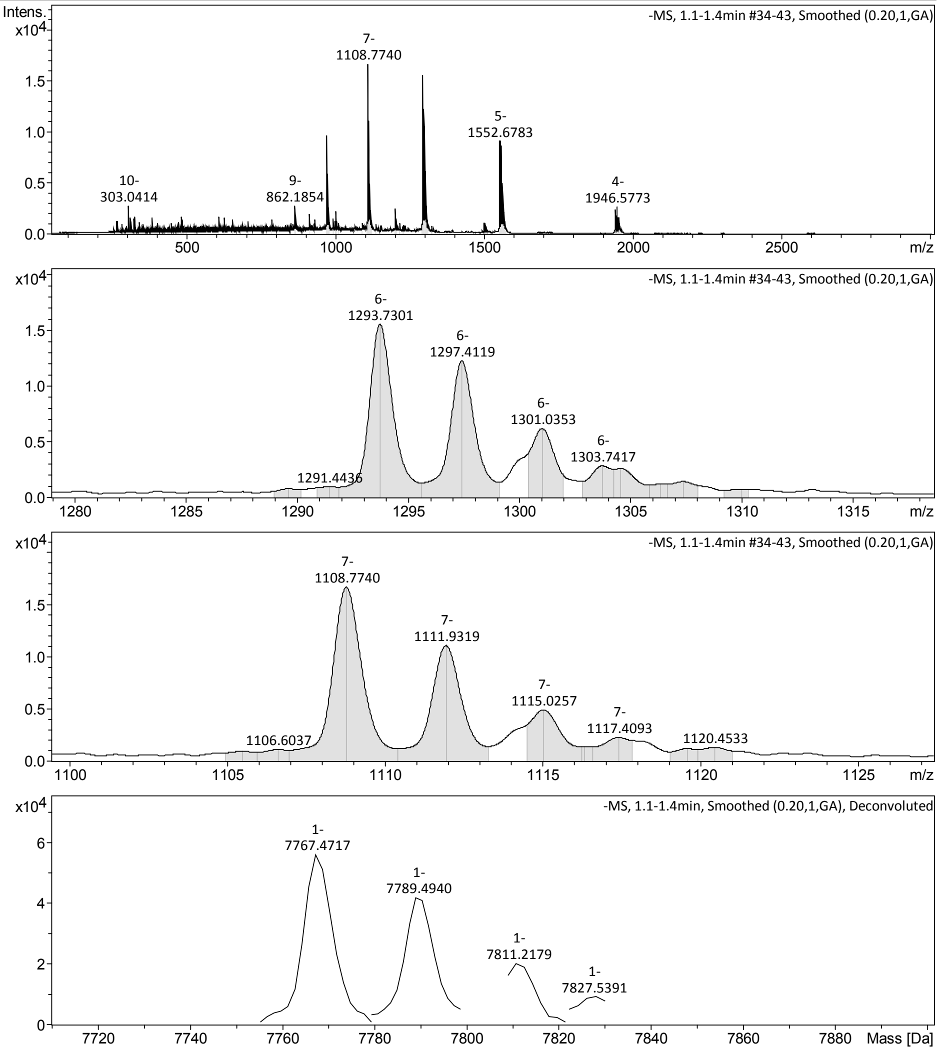

Fig. S32 ESI-TOF-MS spectra of **ON2[+7]**.

Display Report

Analysis Info

Acquisition Date 2022/01/14 10:00:57

Analysis Name D:\Data\yuasa_lab\kanamori\220114\ODN-NBC-8C-000001.d

Method esi_nega_wide.m

Operator BDAL@DE

Sample Name ODN-NBC-8C-

Instrument micrOTOF 213750.10321

Comment

Acquisition Parameter

Source Type	ESI	Ion Polarity	Negative	Set Nebulizer	0.3 Bar
Focus	Not active			Set Dry Heater	180 ‹C
Scan Begin	50 m/z	Set Capillary	1800 V	Set Dry Gas	4.0 l/min
Scan End	3000 m/z	Set End Plate Offset	-500 V	Set Divert Valve	Waste

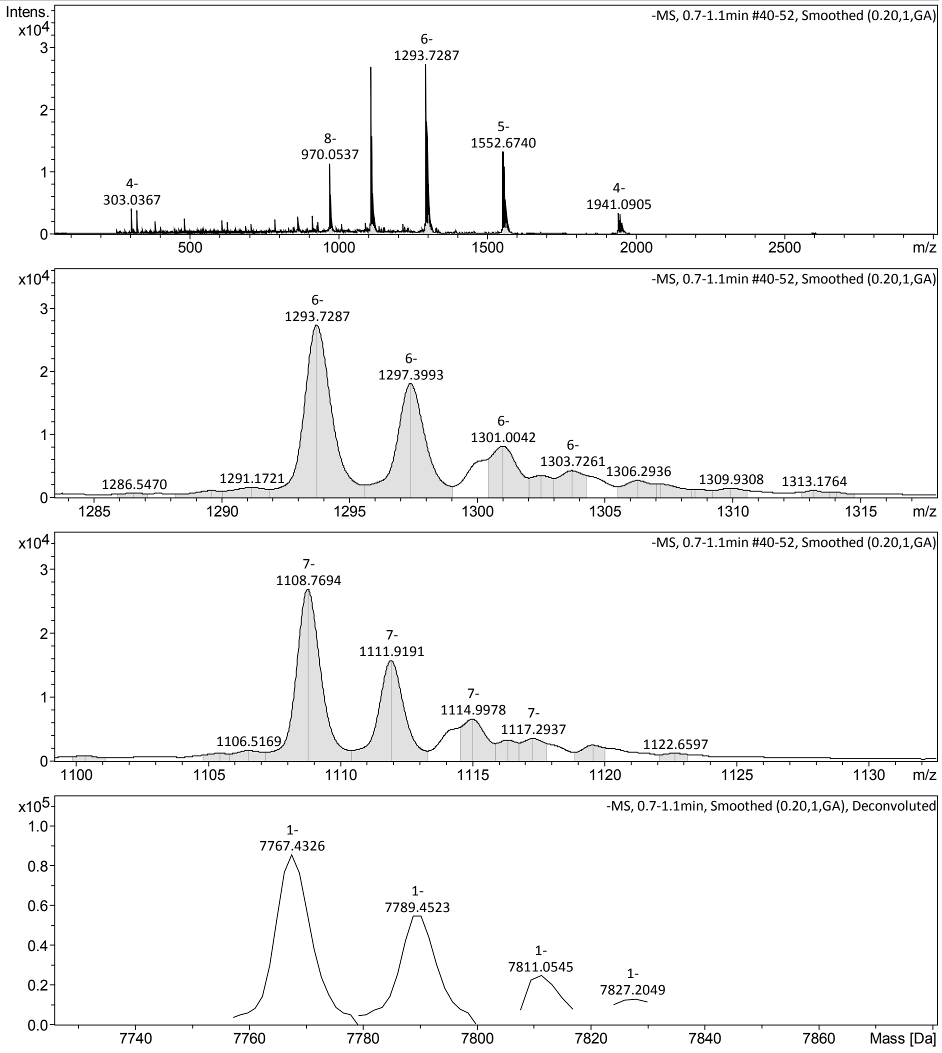

Bruker Compass DataAnalysis 4.2 printed: 2022/01/18 8:34:56 by: BDAL@DE 1 of 1

Fig. S33 ESI-TOF-MS spectra of **ON2[+8]**.

Display Report

Analysis Info

Analysis Name	D:\Data\yuasa_lab\kanamori\220118\ODN-NBC-9C-000001.d	Acquisition Date	2022/01/18 9:37:51
Method	esi_nega_wide.m	Operator	BDAL@DE
Sample Name	ODN-NBC-9C-	Instrument	micrOTOF 213750.10321
Comment			

Acquisition Parameter

Source Type	ESI	Ion Polarity	Negative	Set Nebulizer	0.3 Bar
Focus	Not active			Set Dry Heater	180 ‹C
Scan Begin	50 m/z	Set Capillary	1800 V	Set Dry Gas	4.0 l/min
Scan End	3000 m/z	Set End Plate Offset	-500 V	Set Divert Valve	Waste

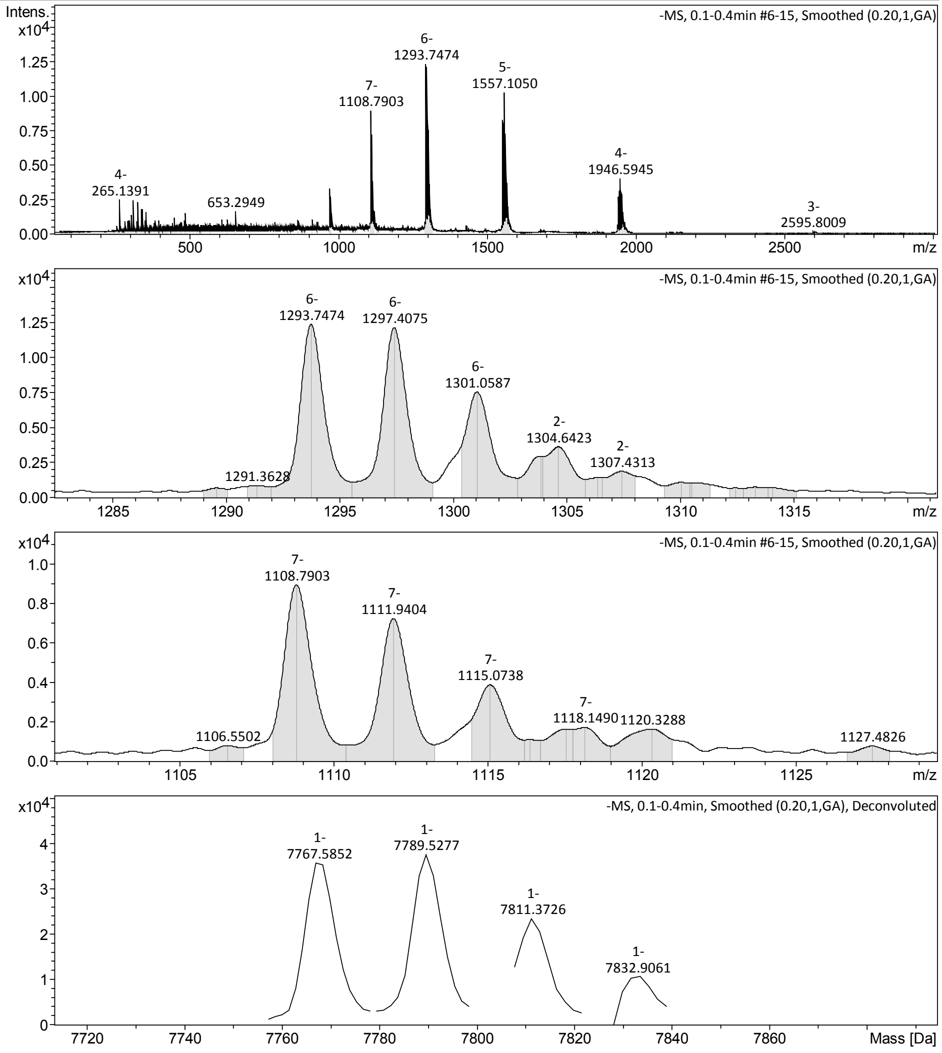

Fig. S34 ESI-TOF-MS spectra of **ON2[+9]**.

Display Report

Analysis Info

Analysis Name	D:\Data\yuasa_lab\kanamori\220114\ODN-NBC-10C-000001.d	Acquisition Date	2022/01/14 10:27:22
Method	esi_nega_wide.m	Operator	BDAL@DE
Sample Name	ODN-NBC-10C-	Instrument	micrOTOF 213750.10321
Comment			

Acquisition Parameter

Source Type	ESI	Ion Polarity	Negative	Set Nebulizer	0.3 Bar
Focus	Not active			Set Dry Heater	180 ‹C
Scan Begin	50 m/z	Set Capillary	1800 V	Set Dry Gas	4.0 l/min
Scan End	3000 m/z	Set End Plate Offset	-500 V	Set Divert Valve	Waste

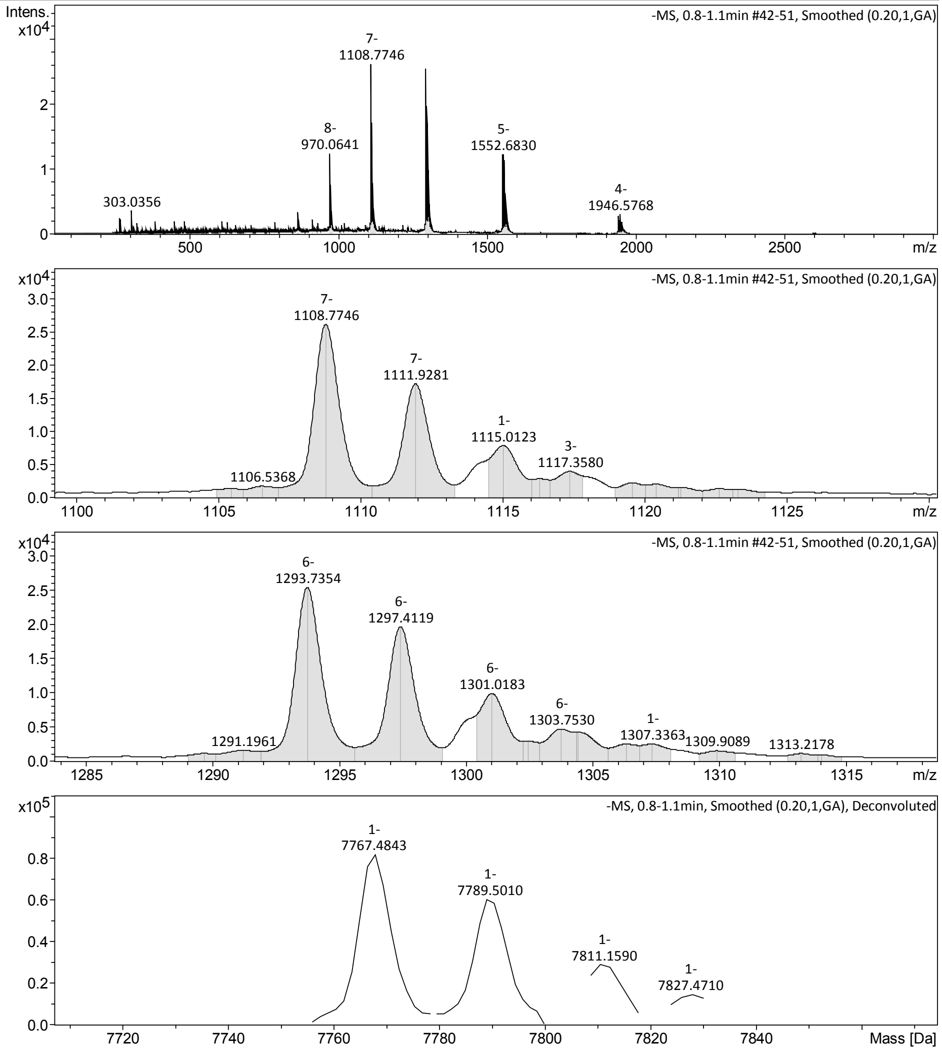

Fig. S35 ESI-TOF-MS spectra of **ON2[+10]**.

Display Report

Analysis Info

Analysis Name	D:\Data\yuasa_lab\kanamori\220118\ODN-NBC-11C-000002.d	Acquisition Date	2022/01/18 10:06:28
Method	esi_nega_wide.m	Operator	BDAL@DE
Sample Name	ODN-NBC-11C-	Instrument	micrOTOF 213750.10321
Comment			

Acquisition Parameter

Source Type	ESI	Ion Polarity	Negative	Set Nebulizer	0.3 Bar
Focus	Not active			Set Dry Heater	180 ‹C
Scan Begin	50 m/z	Set Capillary	1800 V	Set Dry Gas	4.0 l/min
Scan End	3000 m/z	Set End Plate Offset	-500 V	Set Divert Valve	Waste

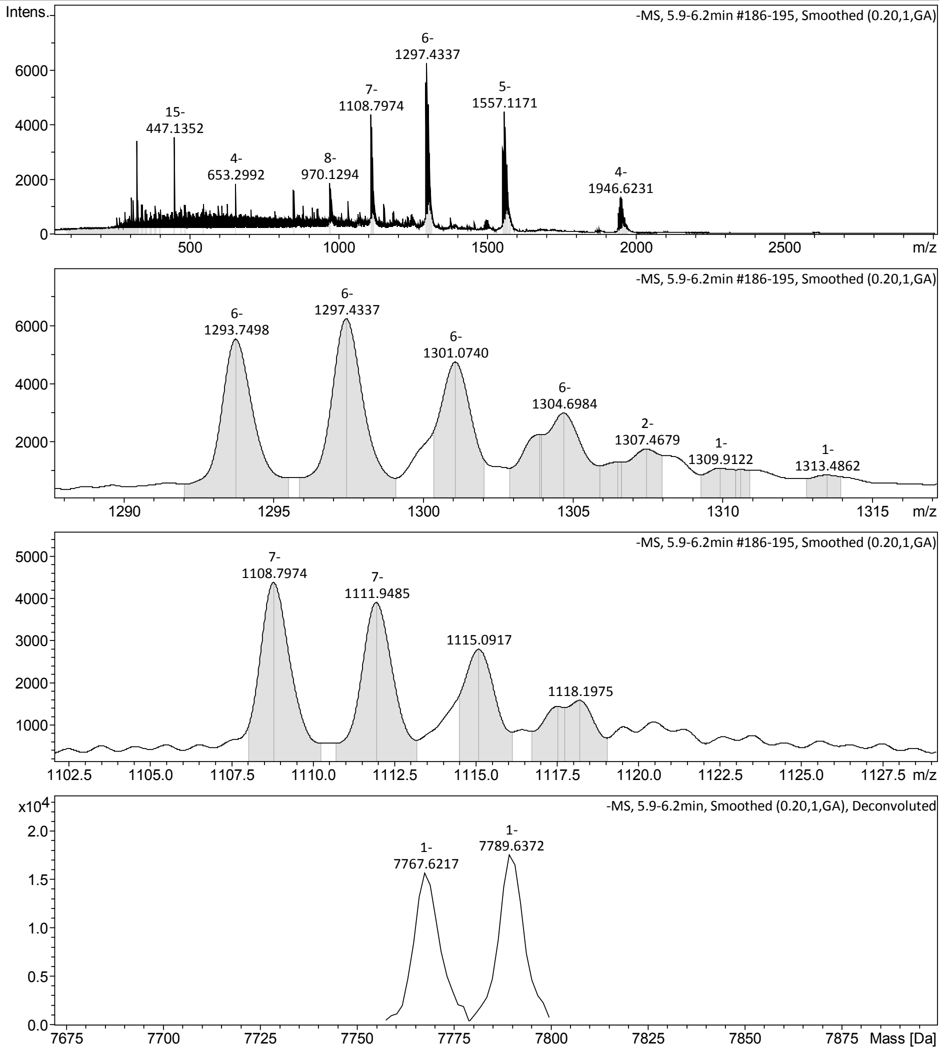

Fig. S36 ESI-TOF-MS spectra of **ON2[+11]**.

Display Report

Analysis Info

Analysis Name	D:\Data\yuasa_lab\kanamori\220302\ODN-NBP-13-000001.d	Acquisition Date	2022/03/01 9:33:16
Method	esi_nega_wide.m	Operator	BDAL@DE
Sample Name	ODN-NBP-13-	Instrument	micrOTOF 213750.10321
Comment			

Acquisition Parameter

Source Type	ESI	Ion Polarity	Negative	Set Nebulizer	0.3 Bar
Focus	Not active			Set Dry Heater	180 ‹C
Scan Begin	50 m/z	Set Capillary	1800 V	Set Dry Gas	4.0 l/min
Scan End	3000 m/z	Set End Plate Offset	-500 V	Set Divert Valve	Waste

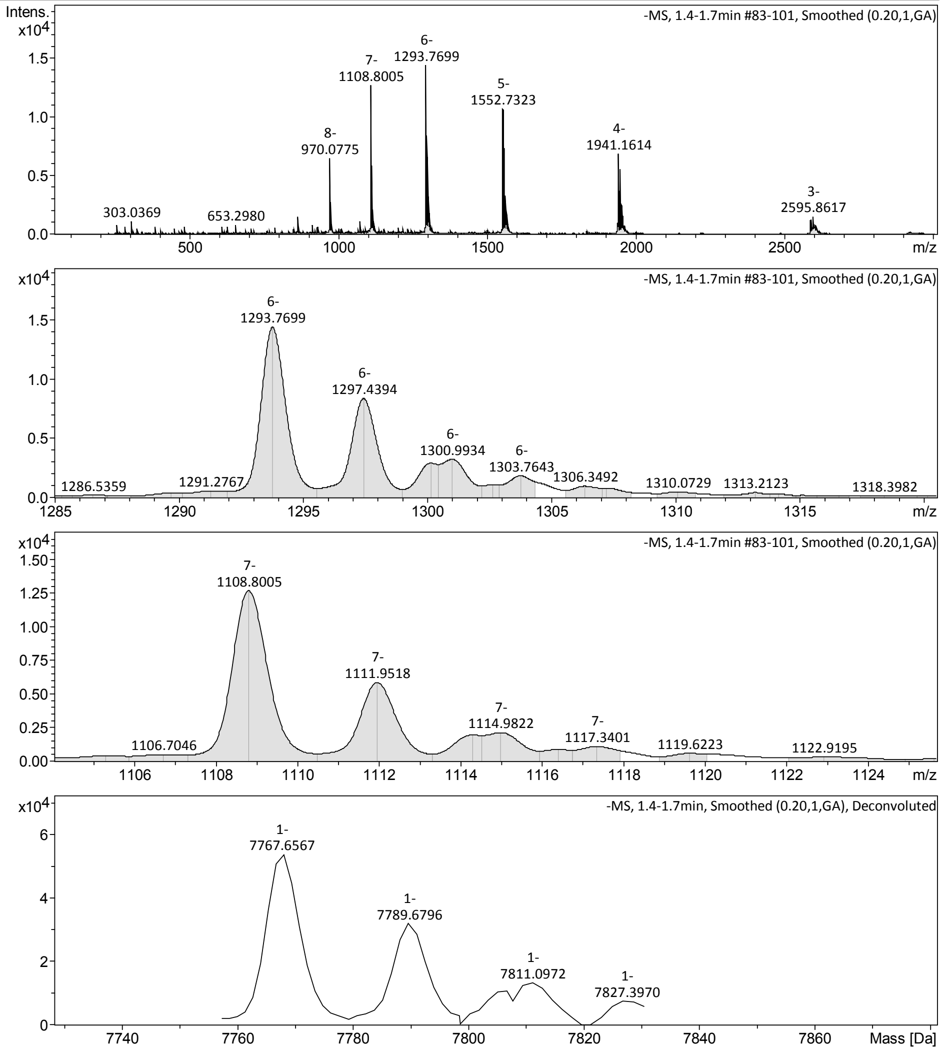

Bruker Compass DataAnalysis 4.2 printed: 2022/03/01 10:52:07 by: BDAL@DE 1 of 1

Fig. S37 ESI-TOF-MS spectra of **ON2[+13]**.

Display Report

Analysis Info

Analysis Name	D:\Data\yuasa_lab\kanamori\220302\ODN-NBP-15-000005.d	Acquisition Date	2022/03/01 10:43:38
Method	esi_nega_wide.m	Operator	BDAL@DE
Sample Name	ODN-NBP-15-	Instrument	micrOTOF 213750.10321
Comment			

Acquisition Parameter

Source Type	ESI	Ion Polarity	Negative	Set Nebulizer	0.3 Bar
Focus	Not active			Set Dry Heater	180 ‹C
Scan Begin	50 m/z	Set Capillary	1800 V	Set Dry Gas	4.0 l/min
Scan End	3000 m/z	Set End Plate Offset	-500 V	Set Divert Valve	Waste

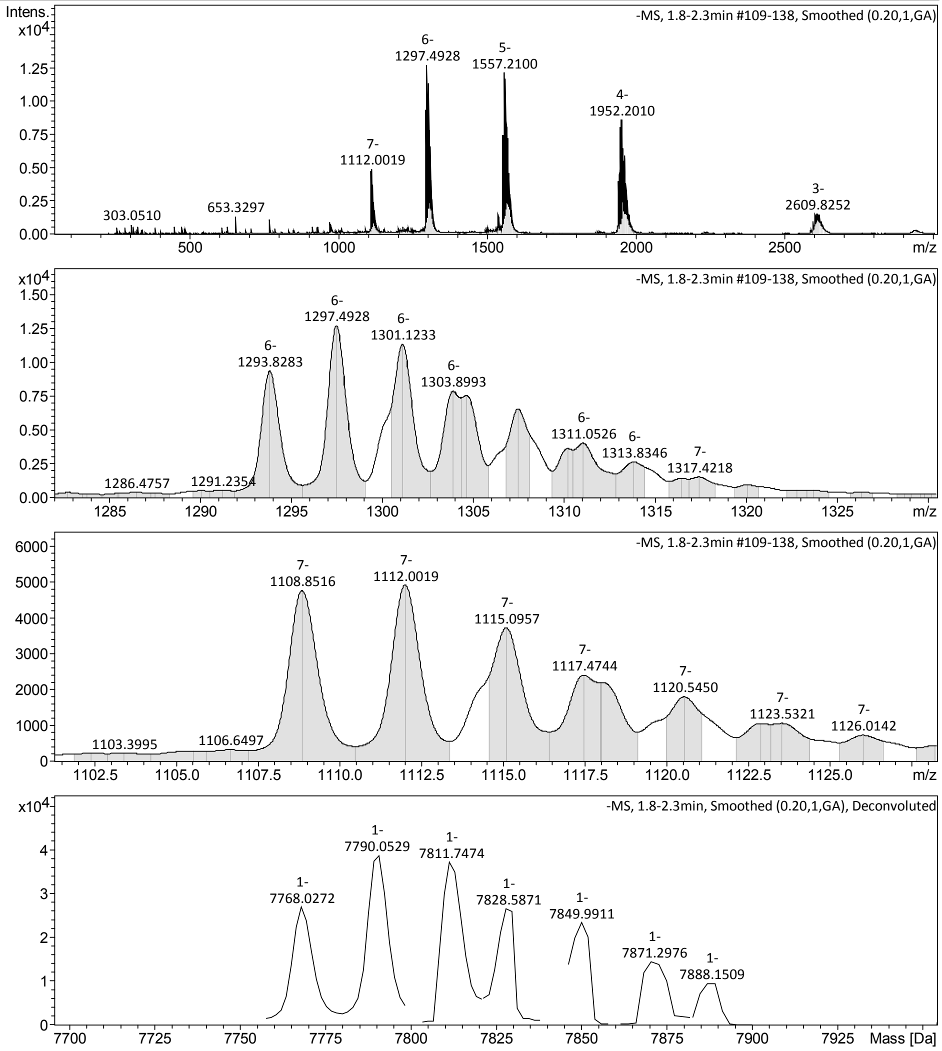

Bruker Compass DataAnalysis 4.2 printed: 2022/03/01 10:54:46 by: BDAL@DE 1 of 1

Fig. S38 ESI-TOF-MS spectra of **ON2[+15]**.

Display Report

Analysis Info

Analysis Name	D:\Data\yuasa_lab\kanamori\220302\ODN-NBP-17-000004.d	Acquisition Date	2022/03/01 10:34:57
Method	esi_nega_wide.m	Operator	BDAL@DE
Sample Name	ODN-NBP-17-	Instrument	micrOTOF 213750.10321
Comment			

Acquisition Parameter

Source Type	ESI	Ion Polarity	Negative	Set Nebulizer	0.3 Bar
Focus	Not active			Set Dry Heater	180 ‹C
Scan Begin	50 m/z	Set Capillary	1800 V	Set Dry Gas	4.0 l/min
Scan End	3000 m/z	Set End Plate Offset	-500 V	Set Divert Valve	Waste

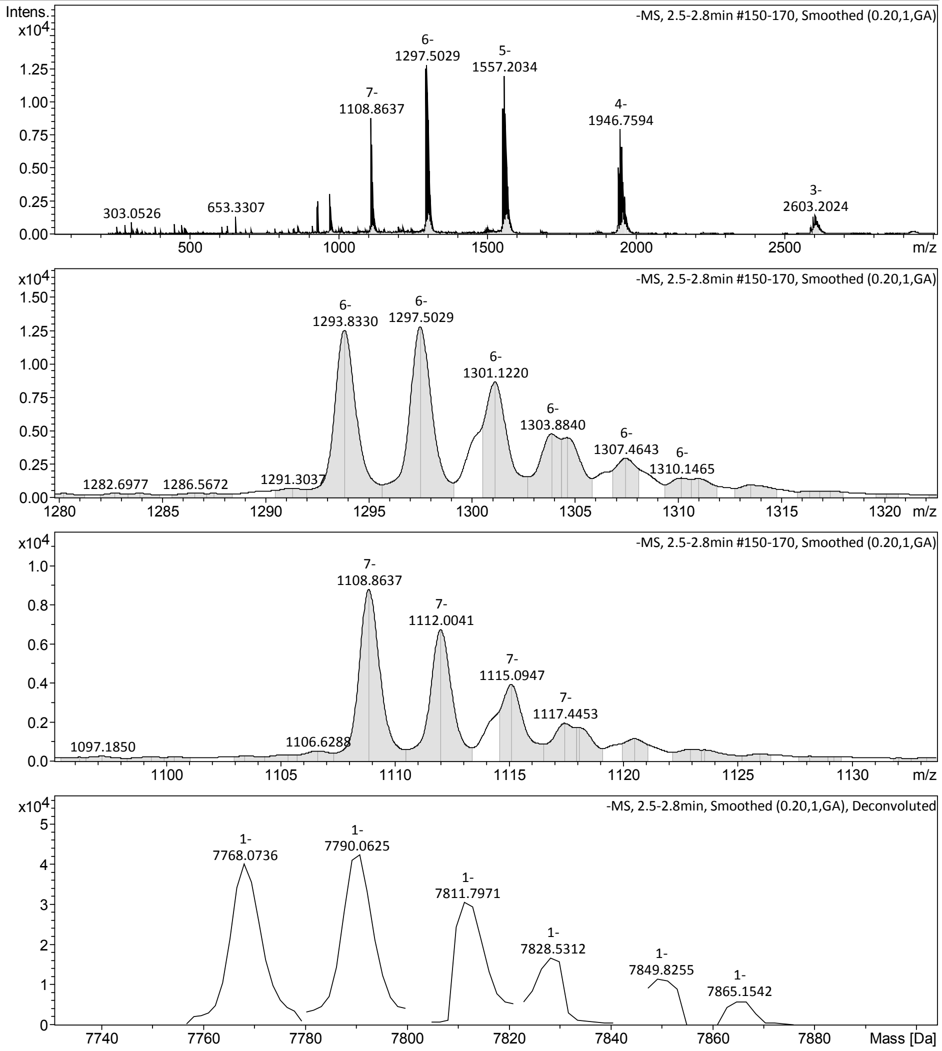

Fig. S39 ESI-TOF-MS spectra of **ON2[+17]**.

Display Report

Analysis Info

Acquisition Date 2022/03/01 10:17:31

Analysis Name D:\Data\yuasa_lab\kanamori\220302\ODN-NBP-19-000001.d

Method esi_nega_wide.m

Operator BDAL@DE

Sample Name ODN-NBP-19-

Instrument micrOTOF 213750.10321

Comment

Acquisition Parameter

Source Type	ESI	Ion Polarity	Negative	Set Nebulizer	0.3 Bar
Focus	Not active			Set Dry Heater	180 ‹C
Scan Begin	50 m/z	Set Capillary	1800 V	Set Dry Gas	4.0 l/min
Scan End	3000 m/z	Set End Plate Offset	-500 V	Set Divert Valve	Waste

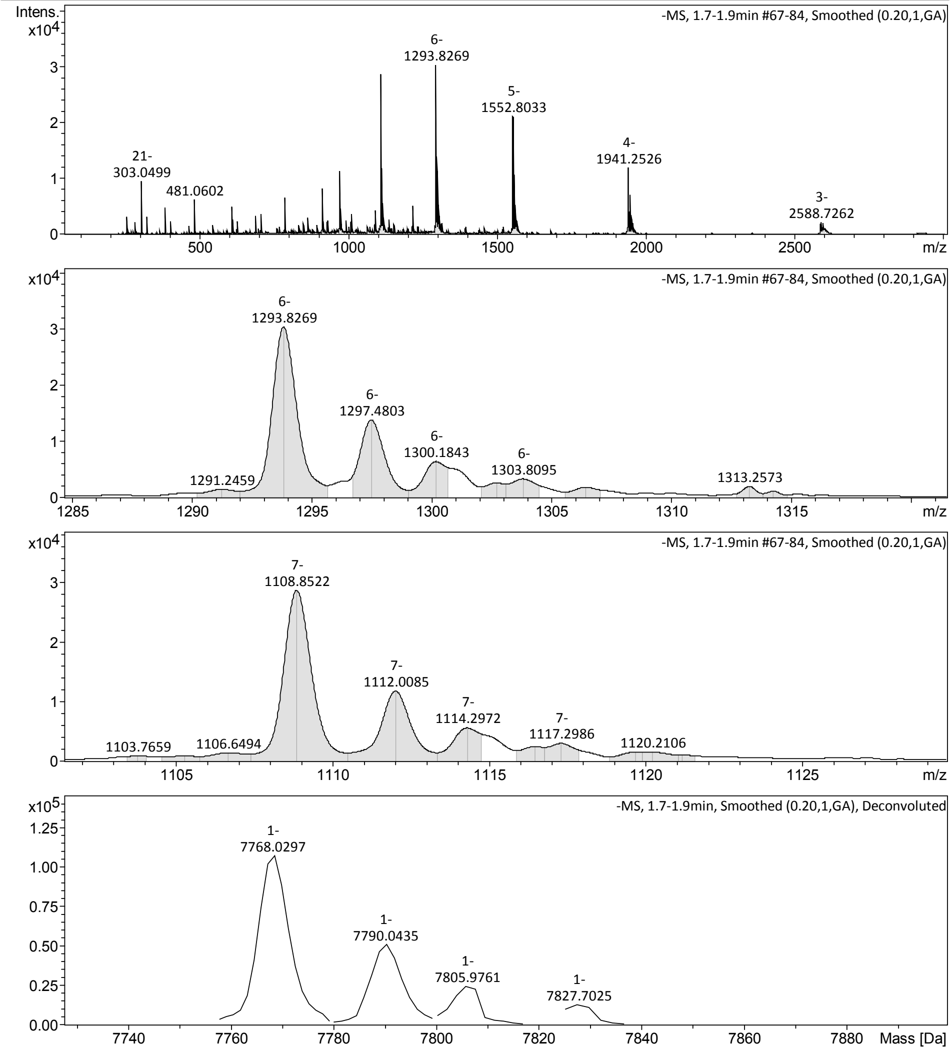

Fig. S40 ESI-TOF-MS spectra of **ON2[+19]**.

Display Report

Analysis Info

		Acquisition Date	2022/08/24 9:55:17
Analysis Name	D:\Data\ofcbunseki\irai\2022\yuasa_lab\kanamori\220825\ODN-NBP-21C-000002.d		
Method	220825.m	Operator	BDAL@DE
Sample Name	ODN-NBP-21C-	Instrument	micrOTOF 213750.10321
Comment			

Acquisition Parameter

Source Type	ESI	Ion Polarity	Negative	Set Nebulizer	0.3 Bar
Focus	Not active			Set Dry Heater	180 ‹C
Scan Begin	400 m/z	Set Capillary	3000 V	Set Dry Gas	4.0 l/min
Scan End	2000 m/z	Set End Plate Offset	-500 V	Set Divert Valve	Waste

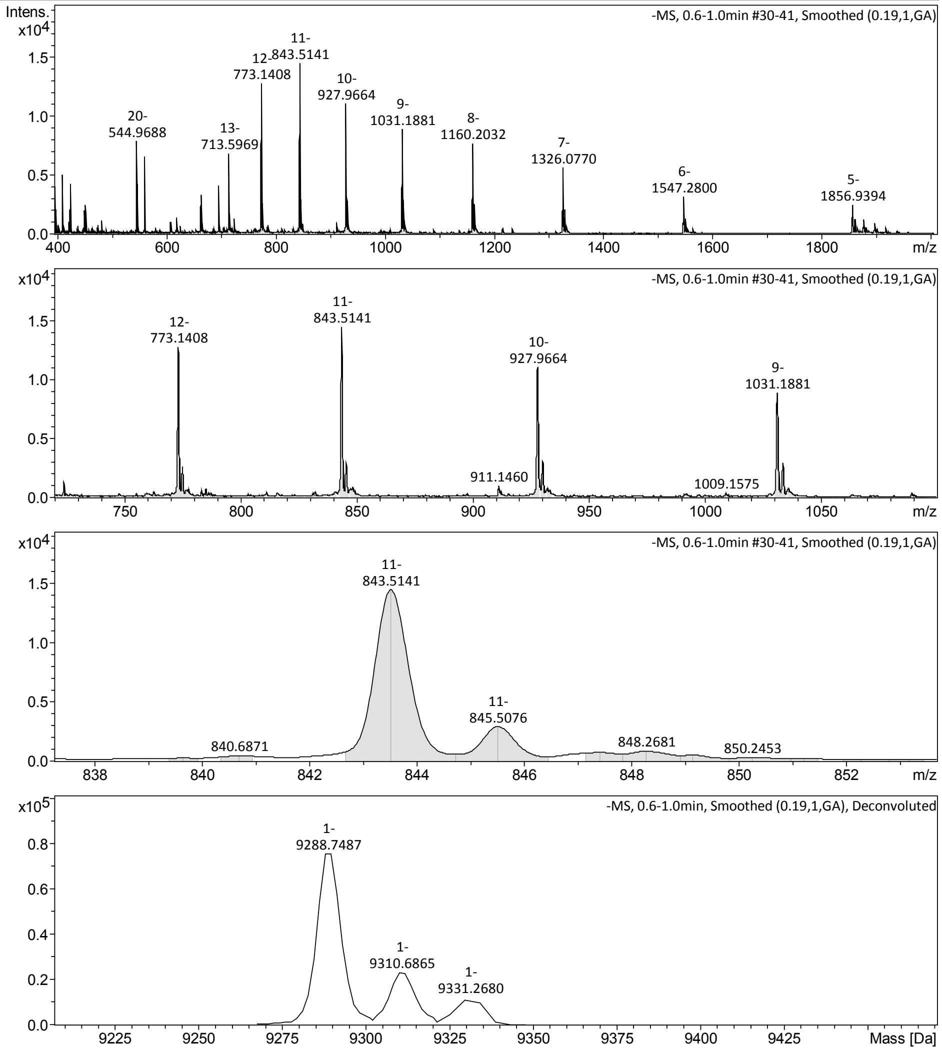

Bruker Compass DataAnalysis 4.2 printed: 2022/08/25 9:52:20 by: BDAL@DE 1 of 1

Fig. S41 ESI-TOF-MS spectra of **ON3[+21]**.

Display Report

Analysis Info

Analysis Name	D:\Data\ofcbunseki\irai\2022\yuasa_lab\kanamori\220825\ODN-NBP-23C-000001.d	Acquisition Date	2022/08/24 10:07:44
Method	220825.m	Operator	BDAL@DE
Sample Name	ODN-NBP-23C-	Instrument	micrOTOF 213750.10321
Comment			

Acquisition Parameter

Source Type	ESI	Ion Polarity	Negative	Set Nebulizer	0.3 Bar
Focus	Not active			Set Dry Heater	180 ‹C
Scan Begin	400 m/z	Set Capillary	3000 V	Set Dry Gas	4.0 l/min
Scan End	2000 m/z	Set End Plate Offset	-500 V	Set Divert Valve	Waste

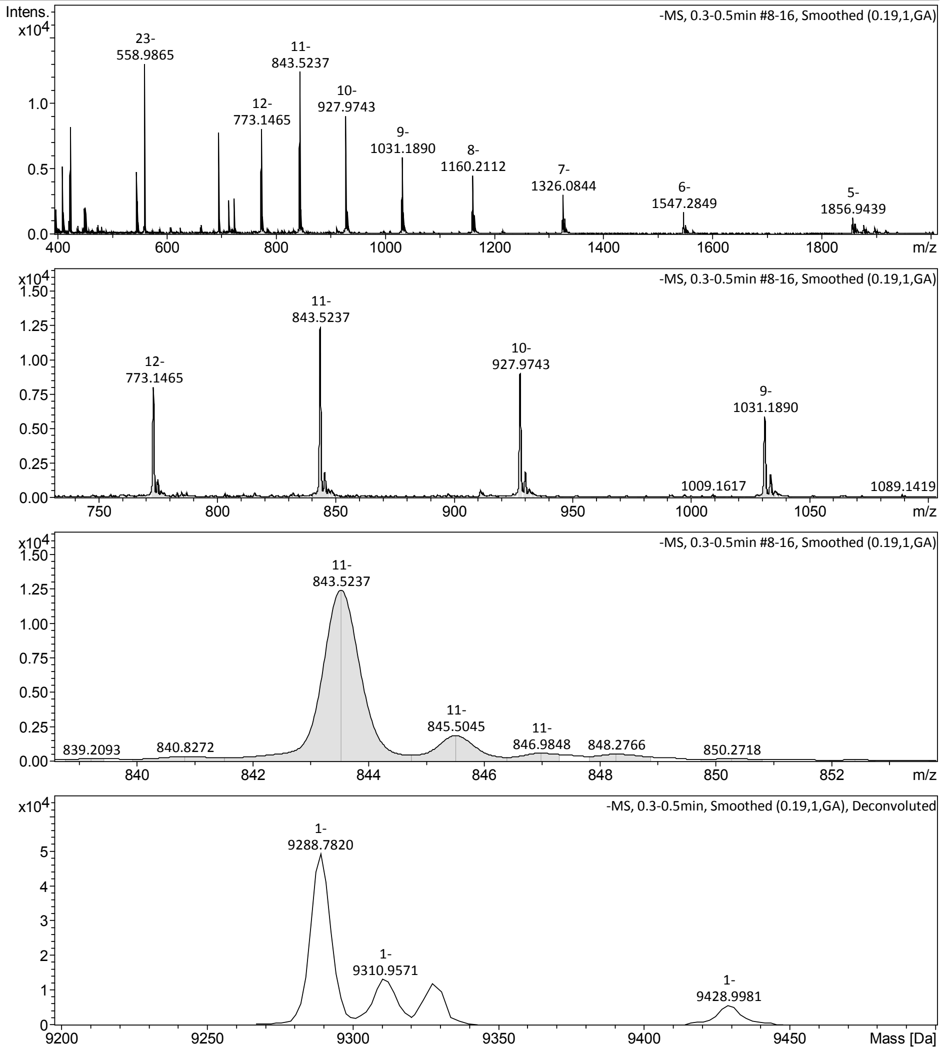

Fig. S42 ESI-TOF-MS spectra of **ON3[+23]**.

2. Measurement of singlet oxygen generation ability of BP-T

The singlet oxygen generation quantum yields (Φ_Δ)-measurement of **BP-T** followed our previous paper.[3] To quantify singlet oxygen, tetraphenylcyclopentadienone (TPCPD) was used as a singlet oxygen scavenger.[4] A 1-mL solution of 200 µM TPCPD in DMF was mixed with a 1-mL solution of biphenyl compound in DMF (the final concentration was set so that the absorbance at the irradiation wavelength was about 0.2) in a quartz cell with an optical path length of 1 cm. It was then irradiated at λ_{max} (346 nm). The excitation light was generated with a 150-W xenon lamp on a JASCO FP-8500 fluorescence spectrometer with 20 nm excitation bandwidth. The intensity of the light source at each wavelength was measured by placing a photodetector (THORLABS S120VC Φ = 9.5 mm) connected to a power meter (THORLABS PM100A) at the sample position. After irradiation, absorption spectra were measured by a spectrophotometer (SHIMAZU UV-2600). The Φ_Δ was calculated with the following equation:[5]

$$\Phi_\Delta = \Phi_\Delta^{Std} \times \frac{m \times F^{Std} \times n^{Std}}{m^{Std} \times F \times n}$$

where "*Std*" is superscripted on symbols for physical quantities of the standard photosensitizer, zinc phthalocyanine (Φ_Δ = 0.56),[6] "*m*" is the slope of the time-dependent absolute absorbance change at 495 nm, "*F*" is the absorption correction factor as deduced by $F = 1\text{-}10^{-\text{Abs}}$ (Abs: Absorbance at the irradiation wavelength), and "*n*" is the relative photon number estimated from measurement of the light power. We thus obtained Φ_Δ of **BP-T** in DMF as 0.29 ± 0.01.

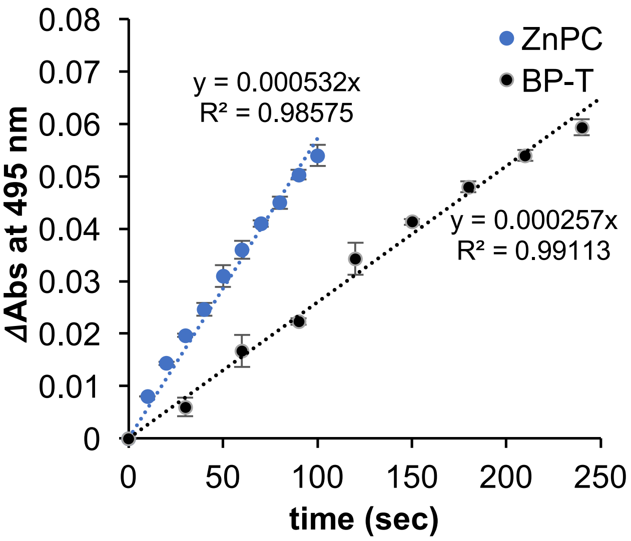

Fig. S43 Plots for time-dependent absolute absorbance changes of tetraphenylcyclopentadienone (TPCPD) in DMF under the photoirradiation in the presence of **BP-T (5)** (irrad at 346 nm) and zinc phthalocyanine (ZnPC) (irrad at 670 nm), the standard.

3. UV-melting temperatures measurements

A 2.0-μM solution of an ON (180 μL) in 10 mM sodium phosphate buffer (pH 7.2) containing 100 mM NaCl was put into a quartz cell and sealed with a silicone cap. First, the solution was kept at 90 °C for 10 min and then cooled to 5 °C at a rate of 0.5 °C/min. Subsequently, the temperature was raised to 90 °C at the same rate, during which the UV absorbances at 260 nm were measured at every 1 °C by UV-Vis spectrophotometer (UV-1900i, Shimadzu). The absorbance values were plotted against temperature to afford a melting curve, which was then differentiated to give a differential curve. The melting temperature (T_m) was determined as the temperature giving the maximum of the differential curve.

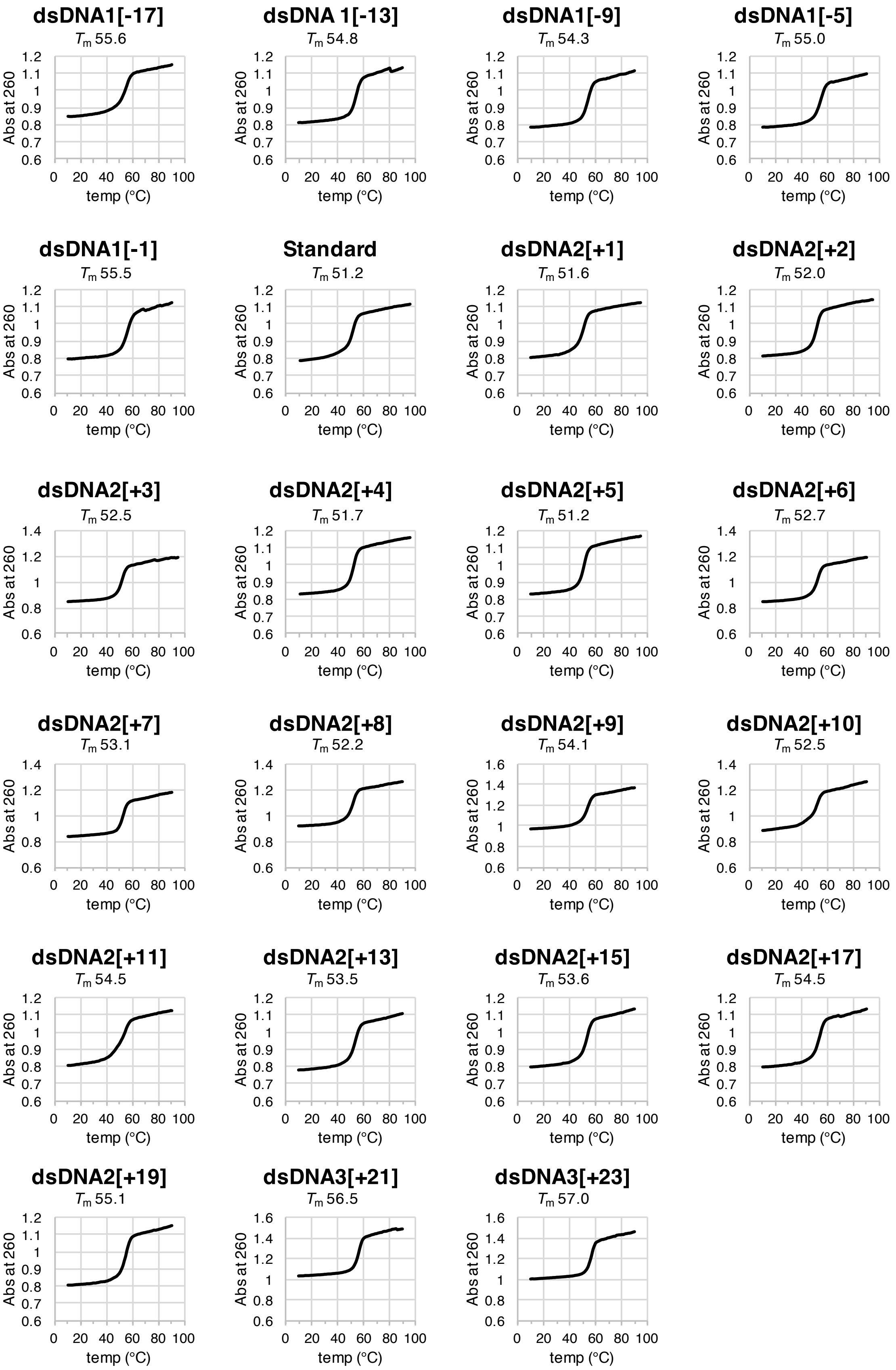

Fig. S44 UV-melting curves of ONs. 2.0 μM **ONs**, 10 mM sodium phosphate buffer (pH 7.2) 100 mM NaCl.

4. Circular dichroism (CD) spectra measurements

A 2.0-μM solution (2.0 mL) of a duplex in a buffer (10 mM sodium phosphate, 100 mM NaCl, pH 7.2) was annealed by heating at 95 °C for 1min and gradually cooling to room temperature. The solution was put into a 1 cm × 1 cm cuvette and measured on a circular dichroism spectrometer (J-1100, JASCO) with the following conditions; scan rage: 190-500 nm, scan speed: 500 nm/min, number of scan: 10 times, temp 25 °C.

5. Photooxidation, enzymatic digestion, and HPLC analysis of ONs

A 2.0-μM solution (150 μL) of a duplex in a buffer (10 mM sodium phosphate, 100 mM NaCl, pH 7.2) was placed into a 600-μL microtube and the duplex was annealed by heating at 95 °C for 1 min and gradually cooling to room temperature. The microtube was placed into a heat block (NDC-100, NISSIN) and photoirradiated with a 365-nm LED light (M365FP1, Thorlabs). The LED light was placed 1 mm above the microtube and irradiation was carried out with a light power of 42 mW/cm^2 at 25 °C for 0, 10, 15, 30 or 60 min.

To the resultant solution were added 12-μL of buffer (100 mM Tris-HCl (pH 8.0), 50 mM $MgCl_2$, 1 M KCl, 0.2% Triton X-100, and 1 mg/mL BSA), phosphodiesterase I (Worthington Biochemical Corporation, 0.5 U/μL, 3 μL), and alkaline phosphatase (Thermo Scientific, 1 U/μL, 5 μL). The mixture was incubated at 37 °C for 3 h on a heat block. Then, the reaction mixture was cooled on ice. To the mixture was added 0.5 M EDTA (2 μL) and the resulting mixture was heated at 90 °C for 1 min to inactivate the enzymes. Then, the mixture was cooled on ice and to it were added distilled water (13 μL) and riboflavin aqueous solution (as internal standard for the HPLC analysis) (15 μL). The mixture was centrifuged at 14000 rpm for 5 min at 4 °C. The solution was filtered (Millex-HV, 0.45 μm) and the filtrate was analyzed on a RP-HPLC system (Hitachi High-Tech, LaChrom Elite) equipped with a Waters X-bridge column (C18 5 μm, 4.6 × 150 mm) at 30 °C (column oven temp) at a flow rate of 1.0 mL/min with the following gradient system:[7] 50 mM NH_4HCOOH with inclusion of 0% CH_3CN/0–5 min, 0–9.5% CH_3CN/5–30 min, 9.5–24.5% CH_3CN/30–45 min, and 24.5–64.5% CH_3CN/45–60 min.

The peak areas of dG, dA, dI (deoxyinosine generated from dA by a deaminase, which may be contaminated in the phosphodiesterase[8]) traced at 260 nm and that of oxodG traced at 300 nm were used for quantification. Each peak area (dG, dA, dI for trace at 260 nm, oxodG for trace at 300 nm) was divided by each ε value (ε_{260} dG: 11500, ε_{260} dA: 1540, ε_{260} dI: 7500, ε_{294} oxodG: 5200) to estimate their concentrations. To precisely estimate the decrements of dG and increments of oxodG in the photooxidation, [dG] and [oxodG] were separately compared with the sum of [dA] and [dI]. The relative amounts (%) of dG and oxodG obtained by n-min photoirradiation were expressed as follows:

$$\mathrm{dG} = \{[\mathrm{dG}]_n/([\mathrm{dA}]_n + [\mathrm{dI}]_n)\}/\{[\mathrm{dG}]_0/([\mathrm{dA}]_0 + [\mathrm{dI}]_0)\} \times 100$$

$$\mathrm{oxodG} = \{[\mathrm{oxodG}]_n/([\mathrm{dA}]_n + [\mathrm{dI}]_n)\}/\{[\mathrm{dG}]_0/([\mathrm{dA}]_0 + [\mathrm{dI}]_0)\} \times 100$$

where [X]n is the concentration of X with n-min photoirradiation.

To study intermolecular crosstalk oxidation of dsDNAs by $^1O_2$, photooxidation of the 2.0 μM **dsDNA5[+10]** and 2.0 μM **dsDNA6** mixture was performed. First, a 4-μM solution of **dsDNA5[+10]** and **dsDNA6** were separately prepared by annealing the corresponding single strand ONs. 75-μL solutions of **dsDNA5[+10]** and **dsDNA6** were mixed just before photoirradiation. Then, the

photoirradiated sample was subjected to the analysis of products in the same manner as mentioned above.

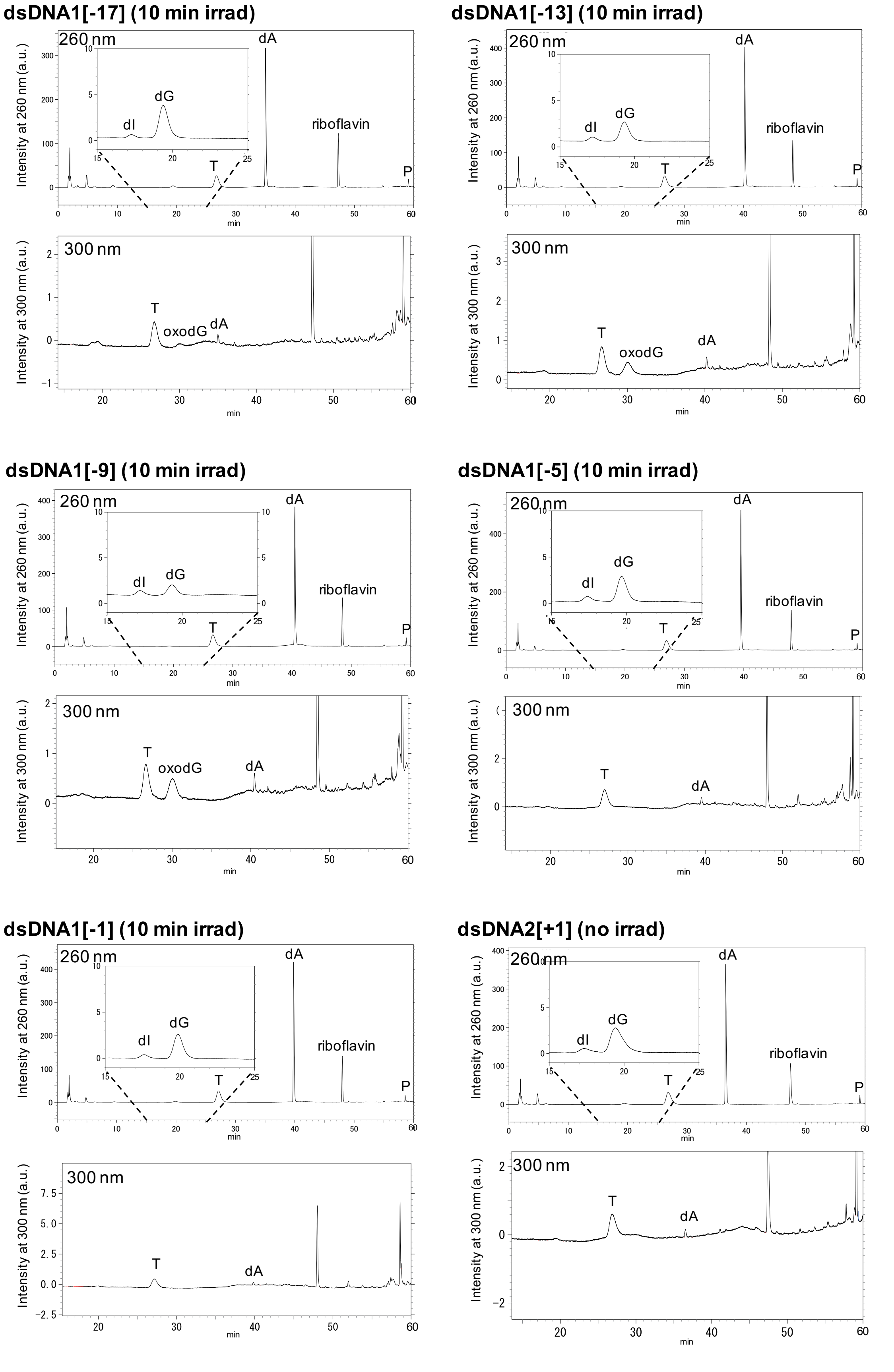

Fig. S45 RP-HPLC analysis of enzymatically digested **dsDNA1[n] (n = -17 ~ -1)**, **dsDNA2[n] (n = +1)**. The upper and lower chromatographs for each dsDNA were recorded respectively at 260 nm (dG, dA, and dI) and 300 nm (oxodG).

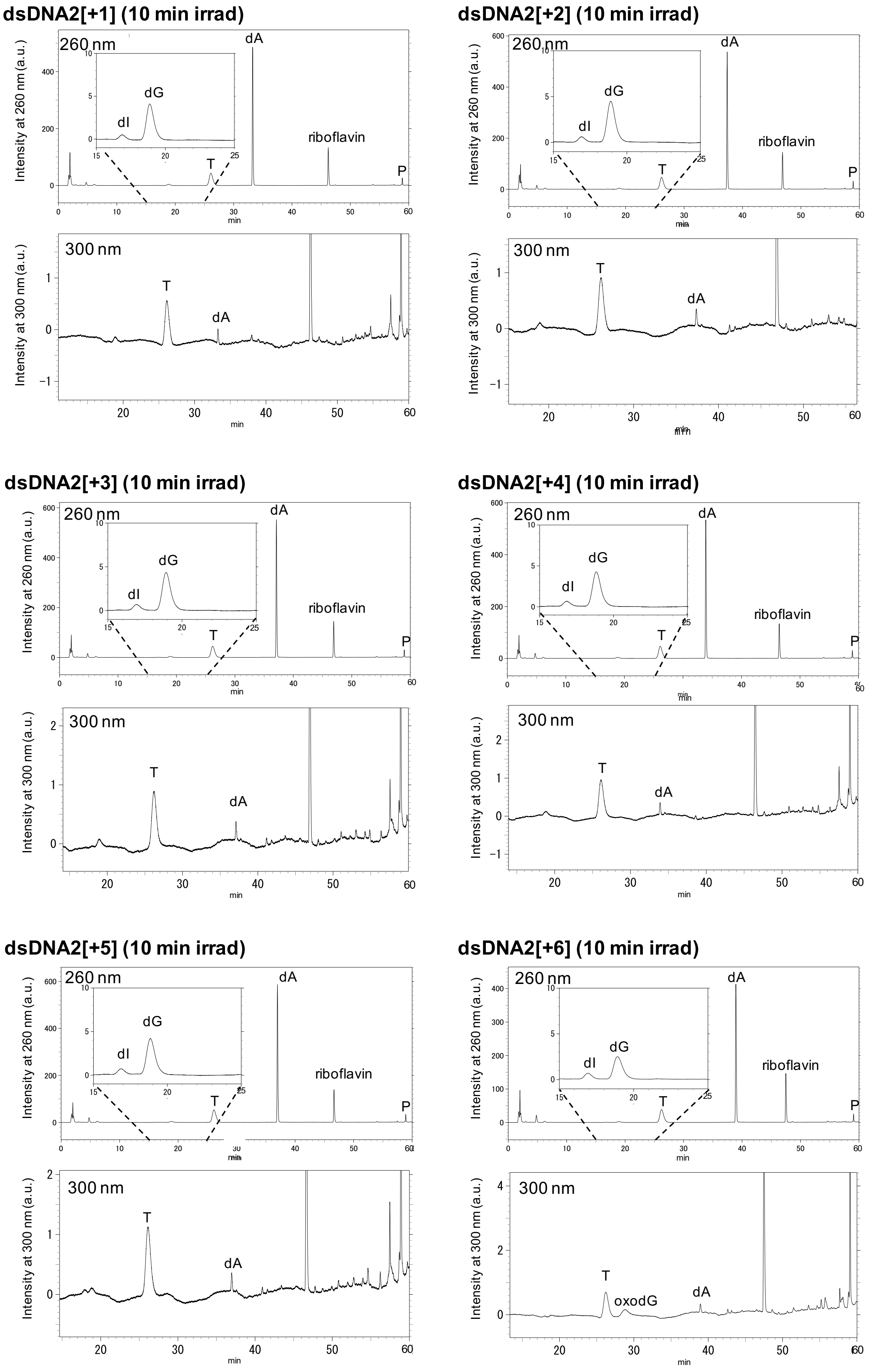

Fig. S46 RP-HPLC analysis of enzymatically digested **dsDNA2[n] (n = +1 ~ +6)**. The upper and lower chromatographs for each dsDNA were recorded respectively at 260 nm (dG, dA, and dI) and 300 nm (oxodG).

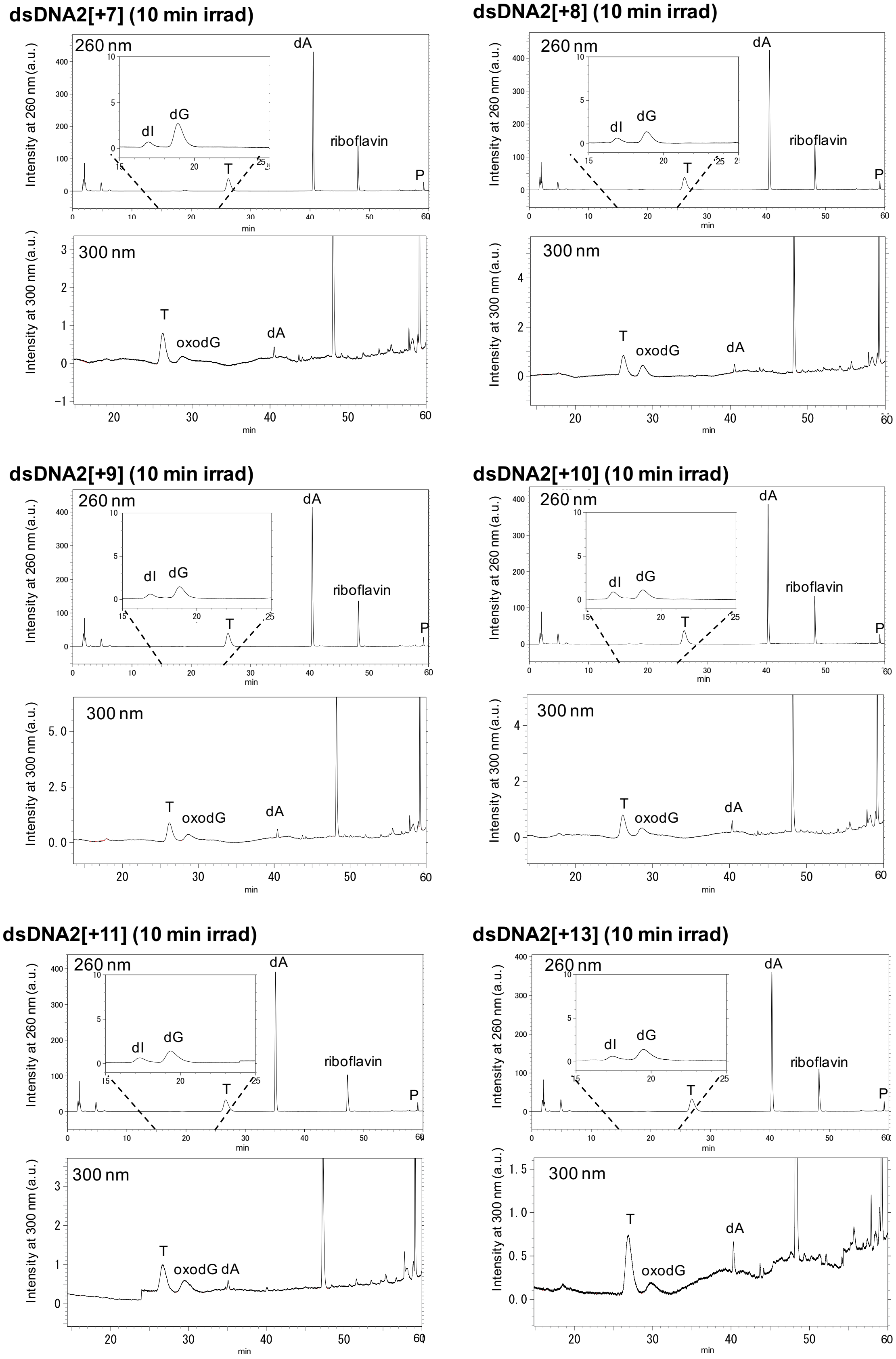

Fig. S47 RP-HPLC analysis of enzymatically digested **dsDNA2[n] (n = +7 ~ +13)**. The upper and lower chromatographs for each dsDNA were recorded respectively at 260 nm (dG, dA, and dI) and 300 nm (oxodG).

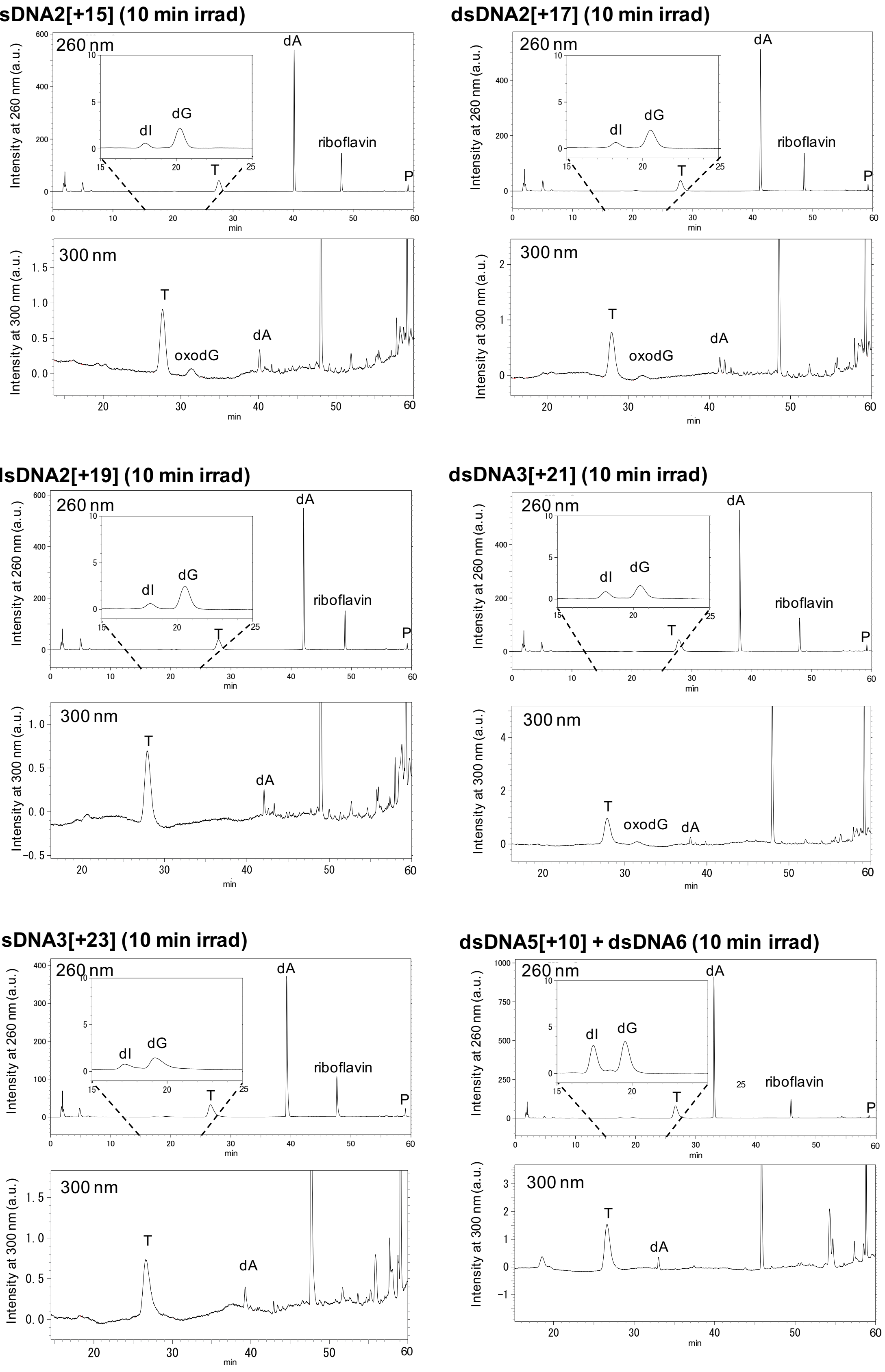

Fig. S48 RP-HPLC analysis of enzymatically digested **dsDNA2[n] (n = +15 ~ +19)**, **dsDNA3[n] (n = +21, +23)**, **dsDNA5[+10]** + **dsDNA6** mixture. The upper and lower chromatographs for each dsDNA were recorded respectively at 260 nm (dG, dA, and dI) and 300 nm (oxodG).

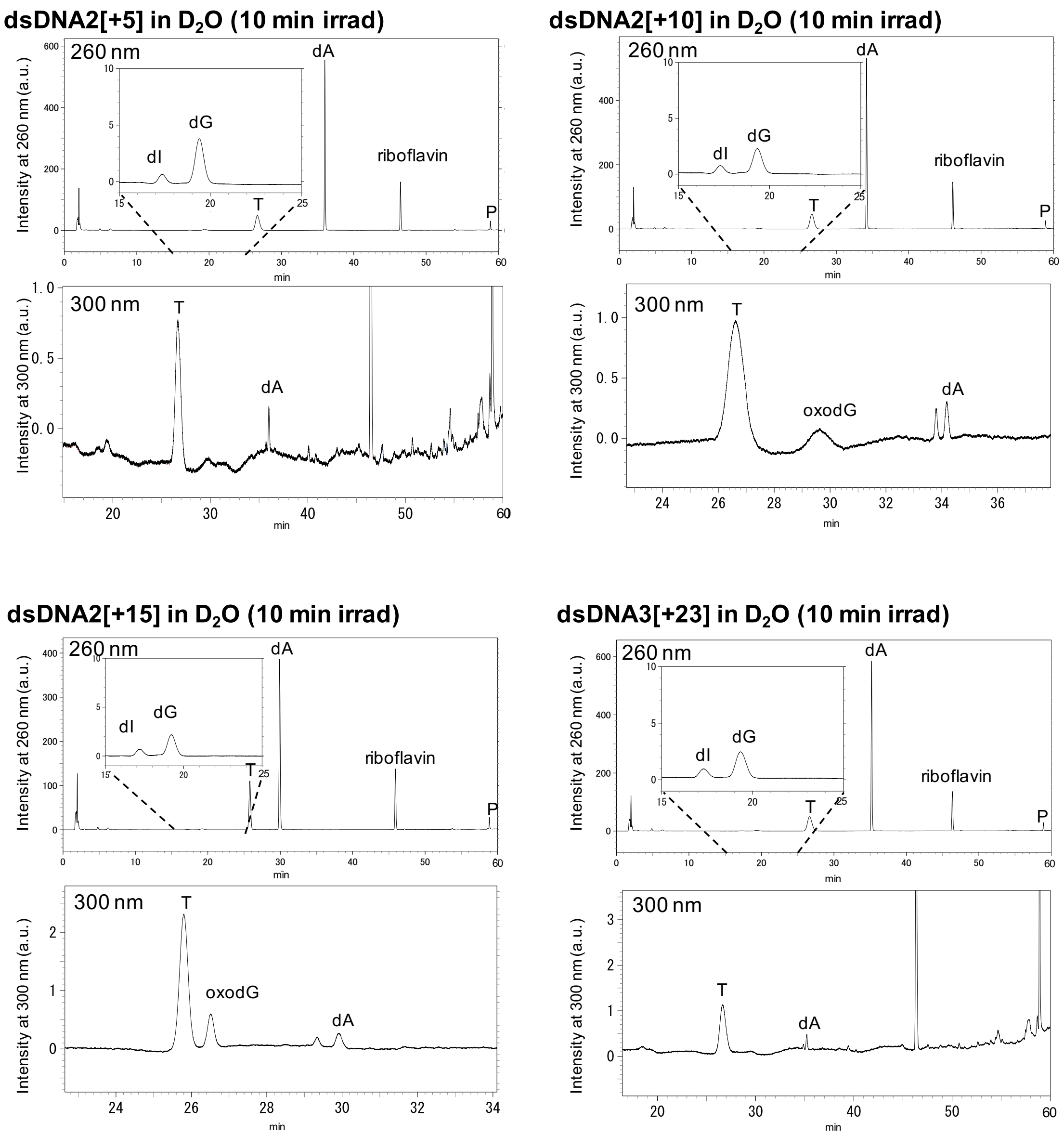

Fig. S49 RP-HPLC analysis of enzymatically digested **dsDNA2[n] (n = +5, +10, +15)**, **dsDNA3[+23]** in D_2O (10 mM phosphate buffer, 100 mM NaCl). The upper and lower chromatographs for each dsDNA were recorded respectively at 260 nm (dG, dA, and dI) and 300 nm (oxodG).

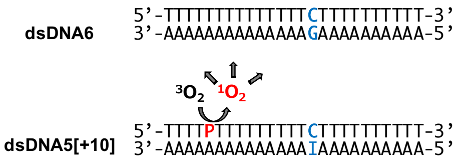

irrad time (min)	remaining dG (%)
0	100
10	93

Fig. S50 Photooxidation analysis of dG after the enzymatic digestion of 2.0 μM **dsDNA5[+10]** and 2.0 μM **dsDNA6** mixture for the study of intermolecular crosstalk oxidation by $^1O_2$.

6. Gel mobility shift analysis of photooxidized ODN

A 2.0-μM solution of dsDNA4[+5] (150 μL) was prepared and photoirradiated for 1 hr in the same manner as mentioned above. After the photoirradiation, a 15-μL portion of the solution was taken and mixed with 1 M aq. piperidine (150 μL) and the resulting mixture was incubated for 45 min at 95 °C. The samples were evaporated to dryness and dissolved in 6 μL of loading buffer. Then, a 4-μL portion of the mixture was loaded on a 20% denaturing PAGE (7 M urea) and electrophoresed at 300 V. After the electrophoresis, the gel was scanned with a fluorescent image scanner (GE Typhoon FLA 9500).

7. $^1O_2$ production analysis of BP-modified ONs by furfuryl alcohol

A 4.0-μM solution (50 μL) of a dsDNA in buffer was mixed with a 200-μM solution (50 μL)) of furfuryl alcohol. The mixture was photoirradiated with 365 nm LED (42 mW/cm^2) at 25 °C for 10 min. Then, the solution was analyzed on an RP-HPLC system (Waters alliance) and the furfuryl alcohol peak area monitored at 216 nm was evaluated. The peak area of furfuryl alcohol without photoirradiation was set as 100%.

Table S3 Analysis of the amount of leaked $^1O_2$ in the photooxidation of dsDNA2[n].

n[a]	furfuryl alcohol (%)[b]
+1	85
+2	95
+3	102
+4	84
+5	81
+6	80
+7	80
+8	79
+9	78
+10	77
+13	77
+15	81
+17	79
+19	80

a. n in dsDNA2[n], b. The HPLC peak area of furfuryl alcohol without photoirradiation was set as 100%.

8. Gel electrophoresis analysis of the photooxidation products

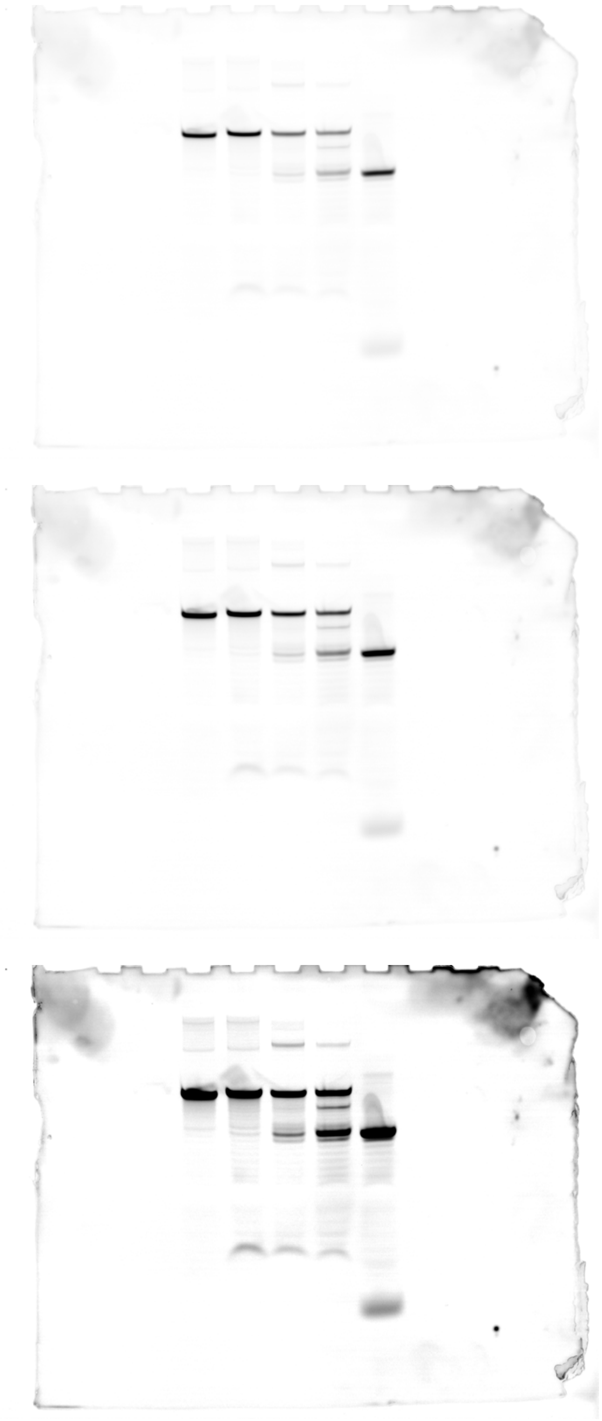

Fig. S51 Gel electrophoresis analysis of the photooxidation products of **dsDNA4[+5]**. Three different contrast images of an identical gel were shown to clarify the gel edges.

9. Photooxidation of ONs in the presence of NaN_3 or mannitol

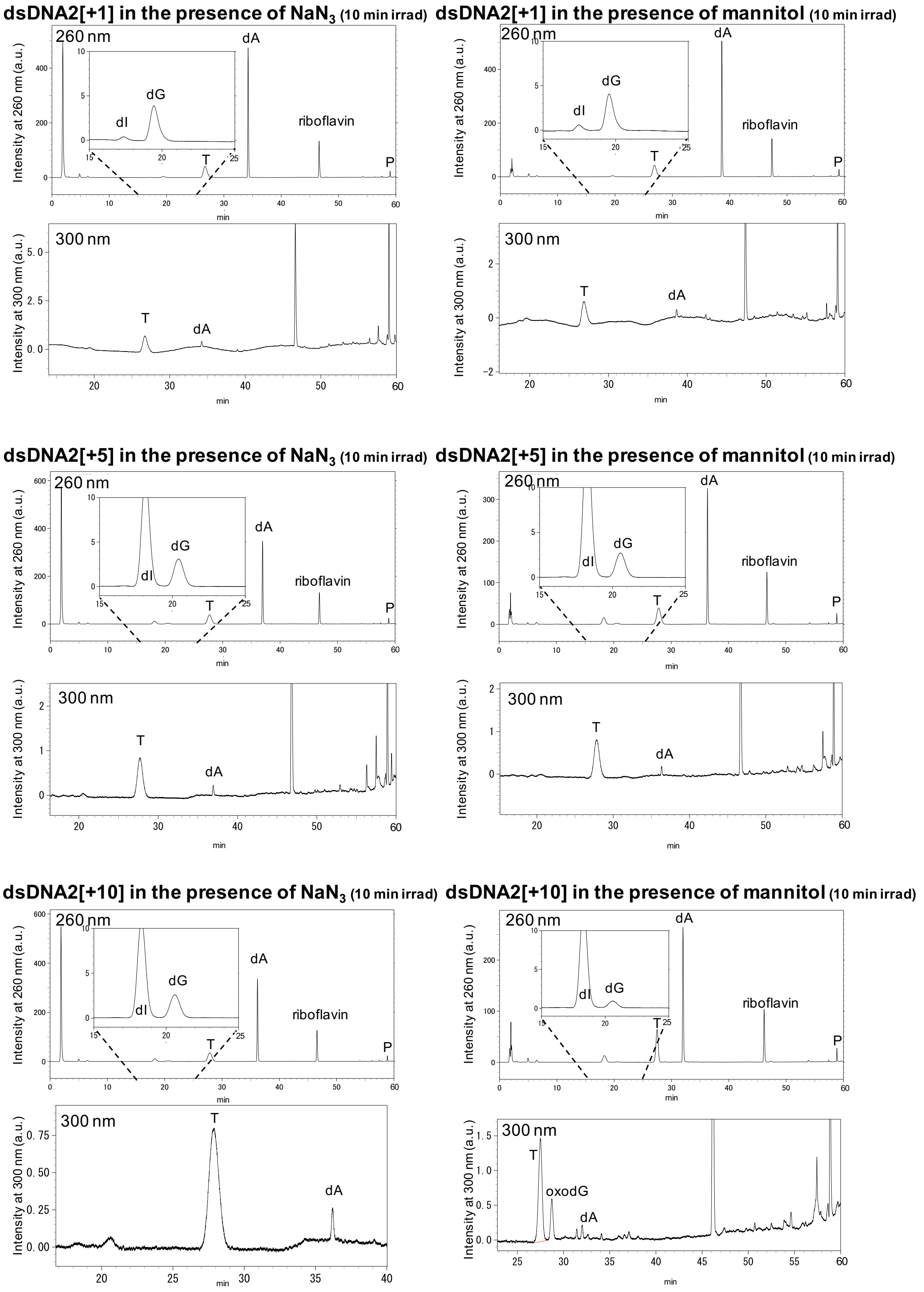

Fig. S52 RP-HPLC analysis of **dsDNA2[n] (n = +1, +5, +10)** in the presence of 50 mM mannitol or NaN_3 as inhibitor for hydroxyl radical and $^1O_2$, respectively. The upper and lower chromatographs for each experiment were recorded respectively at 260 nm (dG, dA, and dI) and 300 nm (oxodG).

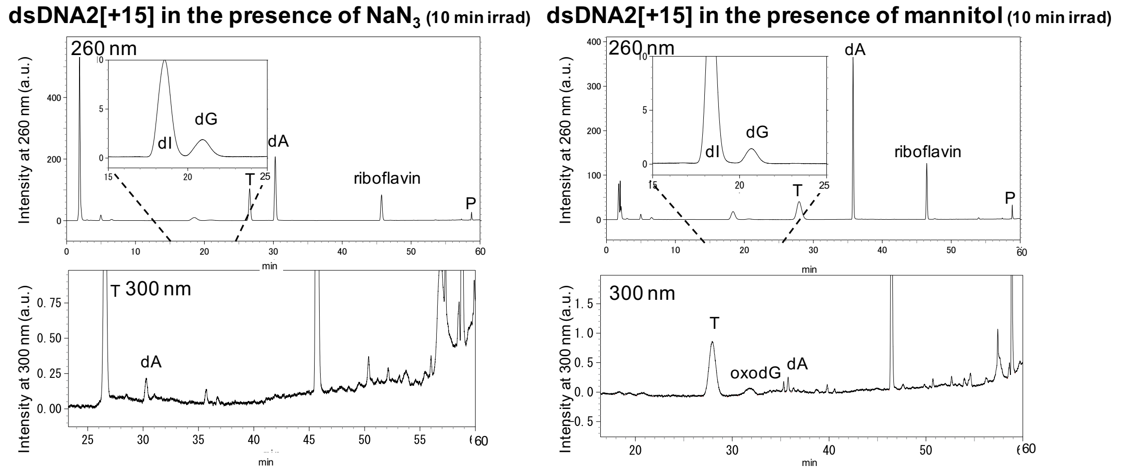

Fig. S53 RP-HPLC analysis of **dsDNA2[+15]** in the presence of 50 mM mannitol or NaN_3 as inhibitor for hydroxyl radical and $^1O_2$, respectively. The upper and lower chromatographs for each experiment were recorded respectively at 260 nm (dG, dA, and dI) and 300 nm (oxodG).

10. References

1. J. T. Goodwin and G. D. Click, *Tetrahedron Lett.*, 1993, **34**, 5549−5552.
2. P. N. Borer, Optical properties of nucleic acids, absorption and circular dichroism spectra. In Fasman,G.D. (ed.), Handbook of Biochemistry and Molecular Biology, 3rd Edn. CRC Press, Cleveland, OH, USA, 1975, Vol. **I**, 589−595.
3. T. Kanamori, Y. Miki, M. Katou, S.-I. Ogura H. Yuasa, *Bioorg. Med. Chem.*, 2022, **61**, 116737.
4. O. Varnavski, T. Goodson, III, *J. Am. Chem. Soc.*, 2020, **142**, 12966−12975.
5. (a) H. S. Jung, J. Han, H. Shi, S. Koo, H. Singh, H.-J. Kim,; J. L. Sessler, J. Y. Lee, J. H. Kim and J. S. Kim, *J. Am. Chem. Soc.*, 2017, **139**, 7595−7602. (b) N. Adarsh, R. R. Avirah and D. Ramaiah, *Org. Lett.*, 2010, **12**, 5720−5723.
6. R. W. Redmond and J. N. Gamlin, *Photochem. Photobiol.,* 1990, **70**, 391−475.
7. K. Kino, I. Saito and H. Sugiyama, *J. Am. Chem. Soc.,* 1998, **120***,* 7373−7374.
8. J. S. Eadie, L. J. McBride, J. W. Efcavitch, L. B. Hoff and R. Cathcart, *Ana. Biochem.,* 1987, **165**, 442−447.
